# Cell-Cycle Regulation of Dynamic Chromosome Association of the Condensin Complex

**DOI:** 10.1016/j.celrep.2018.04.082

**Published:** 2018-05-22

**Authors:** Rahul Thadani, Julia Kamenz, Sebastian Heeger, Sofía Muñoz, Frank Uhlmann

**Affiliations:** 1 Chromosome Segregation Laboratory, The Francis Crick Institute, London NW1 1AT, UK; 2 Laboratory of Biochemistry and Molecular Biology, National Cancer Institute, NIH, Bethesda, MD 20892, USA

**Keywords:** chromosome condensation, mitosis, cell cycle, phosphorylation, condensin, ABC ATPase, *Saccharomyces cerevisiae*

## Abstract

Eukaryotic cells inherit their genomes in the form of chromosomes, which are formed from the compaction of interphase chromatin by the condensin complex. Condensin is a member of the structural maintenance of chromosomes (SMC) family of ATPases, large ring-shaped protein assemblies that entrap DNA to establish chromosomal interactions. Here, we use the budding yeast *Saccharomyces cerevisiae* to dissect the role of the condensin ATPase and its relationship with cell-cycle-regulated chromosome binding dynamics. ATP hydrolysis-deficient condensin binds to chromosomes but is defective in chromosome condensation and segregation. By modulating the ATPase, we demonstrate that it controls condensin’s dynamic turnover on chromosomes. Mitosis-specific phosphorylation of condensin’s Smc4 subunit reduces the turnover rate. However, reducing turnover by itself is insufficient to compact chromosomes. We propose that condensation requires fine-tuned dynamic condensin interactions with more than one DNA. These results enhance our molecular understanding of condensin function during chromosome condensation.

## Highlights

• The budding yeast condensin ATPase promotes chromosome condensation
• The ATPase controls the dynamic chromosome binding cycle of the complex
• Smc4 phosphorylation by cyclin-dependent kinase adjusts the turnover
• Chromosome condensation requires fine-tuning of condensin behavior

## Graphical Abstract

**Figure:**
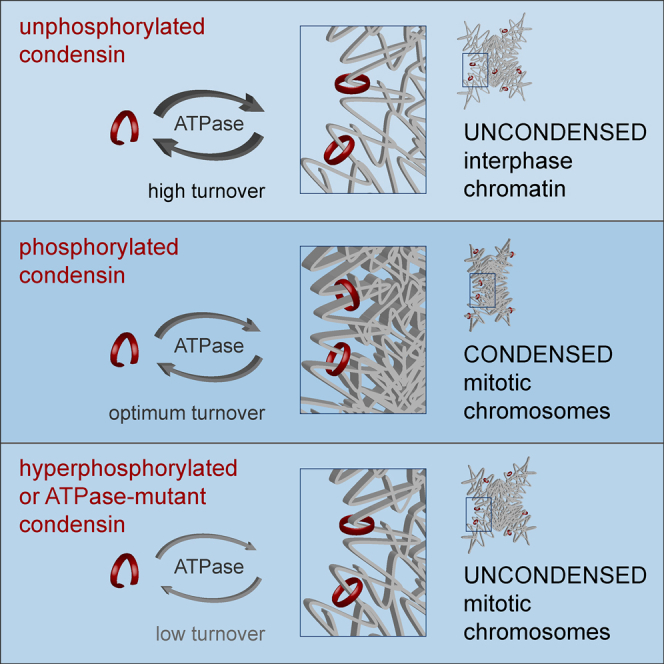

## Teaser

The condensin complex is a key determinant of mitotic chromosome formation. Thadani et al. study the dynamic binding of condensin to chromosomes. They reveal how condensin turnover is regulated by its ATPase and by cell-cycle phosphorylation. Chromosome condensation in mitosis requires fine-tuning of this dynamic behavior.

## Introduction

The condensin complex is a key structural component of mitotic chromosomes (Hirano, 2016, Uhlmann, 2016). It consists of two structural maintenance of chromosomes (SMC) subunits, Smc2 and Smc4, that constitute the circumference of the condensin ring. They dimerize via a hinge domain on one side of the ring and, on the other side, at a pair of ATPase head domains that form the catalytic core of the complex. A kleisin subunit, Brn1 in budding yeast, bridges both ATPase heads. The kleisin subunit also recruits two additional HEAT repeat subunits to the condensin complex (Ycg1 and Ycs4 in budding yeast) (Onn et al., 2007). The ATPase motifs of vertebrate condensin are essential for chromosome condensation, and continual ATP hydrolysis is required to maintain the condensed state (Hudson et al., 2008, Kinoshita et al., 2015). *In vitro*, ATP hydrolysis by condensin promotes DNA supercoiling, but how this relates to chromosome condensation is not yet understood (Kimura and Hirano, 1997). Human condensin binds to chromosomes dynamically. Of the two human condensin isoforms, condensin II appears to turn over rapidly on chromosomes in interphase and bind stably during mitosis. Condensin I in turn accesses chromosomes after nuclear envelope breakdown and maintains dynamic chromosome association throughout mitosis (Gerlich et al., 2006). Simulations of chromatin chain behavior suggest that stabilization of stochastic condensin-mediated DNA-DNA interactions provides a potent driving force for chromosome compaction. An alternatively proposed condensation mechanism, that of loop extrusion or loop expansion, could also be aided by an increased mitotic condensin residence time (Alipour and Marko, 2012, Cheng et al., 2015, Fudenberg et al., 2016, Thadani et al., 2012). How dynamic chromosome binding is linked to condensin’s ATPase activity and how cell-cycle-dependent condensin modifications regulate this dynamic binding cycle are incompletely understood.

## Results

### The Condensin ATPase Is Essential for Yeast Cell Proliferation

To study the condensin ATPase, we generated a series of mutations in conserved residues of budding yeast Smc2 and Smc4 that were designed to disrupt aspects of the ATPase cycle, based on previous studies of SMC ATPases (Arumugam et al., 2003, Hopfner et al., 2000, Lammens et al., 2004, Lengronne et al., 2006, Weitzer et al., 2003). These include mutations in the Walker A motif (Smc2 K38A and Smc4 K191A) to disrupt ATP binding, the signature motif to prevent SMC head engagement (Smc2 S1085R and Smc4 S1324R), the Walker B motif to slow or prevent ATP hydrolysis (Smc2 E1113Q or E1113D and Smc4 E1352Q or E1352D), and an arginine finger to reduce DNA stimulation of ATP hydrolysis (Smc2 R58A or R58K and Smc4 R210A or R210K). The mutant proteins were expressed with C-terminal 3HA epitope tags from an ectopic locus under control of their authentic promoters. In the same strains, the endogenously encoded Smc2 or Smc4 proteins could be conditionally depleted by taking advantage of auxin-inducible degrons (*aid*) (Kubota et al., 2013).

We first tested the ability of ATPase mutant Smc2 and Smc4 to support cell growth. Smc2 or Smc4 depletion leads to loss of viability, which is restored by ectopic expression of wild-type Smc2 or Smc4 (Figure S1). In contrast, most ATPase mutants did not support cell growth, except Smc2 R58K, Smc4 R210K, and Smc4 R210A. This confirmed that like in vertebrates, the ATPase is essential for the function of budding yeast condensin but that certain alterations to the arginine fingers, especially of the Smc4 ATPase, are tolerated. Our findings are consistent with a previous report that ATP binding or head engagement mutants of the budding yeast Smc2 and Smc4 subunits fail to support cell growth (Stray and Lindsley, 2003). Quantitative western blotting confirmed that all ATPase mutant variants were expressed as full-length proteins, albeit at levels somewhat reduced compared to the wild-type control (Figures S2C and S2F).

### Condensin Associates with Chromosomes Independently of Its ATPase

**Figure 1:**
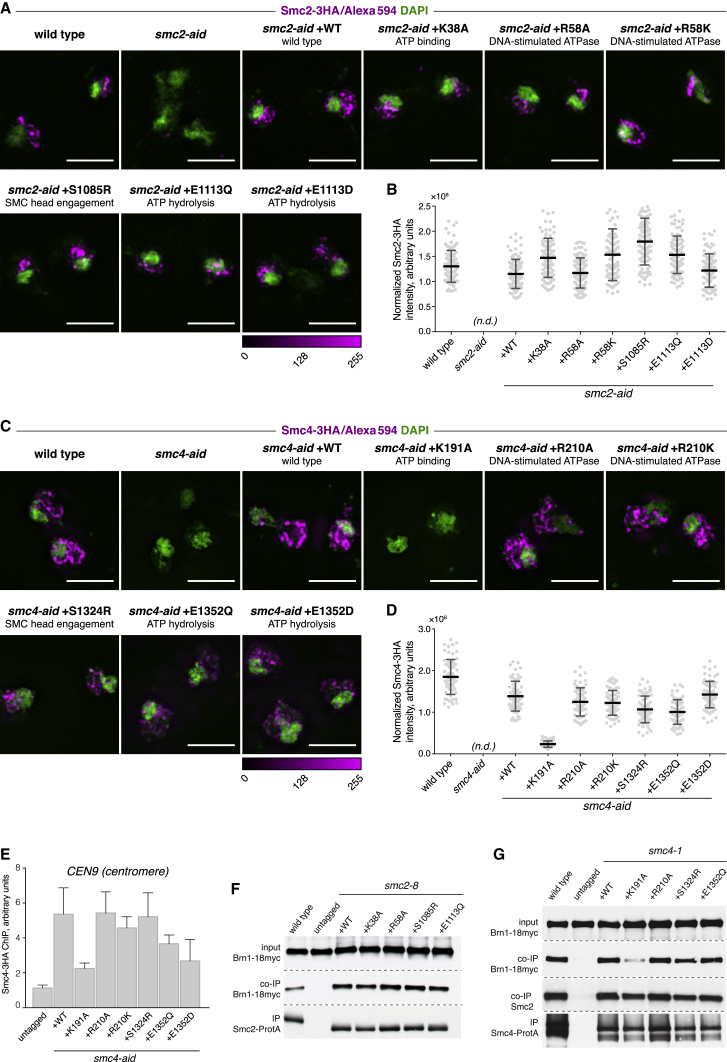
ATPase-Independent Chromosome Binding of Condensin (A) Chromosome spreads of wild-type or *smc2-aid* cells in metaphase expressing ectopic 3HA-tagged wild-type or ATPase mutant Smc2. Cells were synchronized in G1 with α factor and released into a nocodazole-induced metaphase arrest. Chromosome spreads were stained with DAPI and anti-HA/Alexa Fluor 594 antibodies. Scale bars represent 4 μm. (B) Quantification of the Smc2-3HA staining intensities in (A), normalized by cellular expression levels assessed by immunoblotting. Error bars represent mean ± SD (n ≥ 92). (C) Chromosome spreads of wild-type or *smc4-aid* cells in metaphase expressing ectopic 3HA-tagged wild-type or ATPase mutants of Smc4, as in (A). (D) Quantification of the Smc4-3HA staining intensities in (C), normalized by cellular expression levels assessed by immunoblotting. Error bars represent mean ± SD (n ≥ 63). (E) Chromatin immunoprecipitation (ChIP)-qPCR signal of Smc4-3HA at *CEN9*, normalized to a negative binding site, in *smc4-aid* cells in metaphase expressing ectopic wild-type or ATPase mutants of Smc4. Error bars represent mean ± SEM (n = 3). (F) Coimmunoprecipitation of Brn1-18myc with ATPase mutants of Smc2, as assessed following their immunoprecipitation by means of a Protein A tag in a temperature-sensitive *smc2-8* background. (G) Coimmunoprecipitation of Brn1-18myc with ATPase mutants of Smc4, as assessed following their immunoprecipitation by means of a Protein A tag in a temperature-sensitive *smc4-1* background. See also Figure S1 for a viability assay of cells harboring condensin SMC ATPase mutations; Figure S2 for confirmation of cell-cycle synchrony, raw staining intensities, and protein expression levels used for normalization; and Figure S3 for ChIP-qPCR analyses at additional loci.

We next examined the relationship between condensin’s ATPase and chromosome binding. Following endogenous Smc2 depletion and cell synchronization in mitosis (Figure S2A), we visualized chromosome association of ectopic Smc2 by spreading yeast chromosomes on glass slides, followed by antibody staining against its C-terminal 3HA epitope tag. Soluble nuclear content is lost during this procedure (Loidl et al., 1991), and only chromatin-bound Smc2 is expected to be detected. A strain in which endogenous Smc2 was tagged with an equivalent 3HA epitope tag (wild-type) and a strain in which Smc2 was depleted (*smc2-aid*) served as positive and negative controls, confirming staining specificity. The antibody signal was readily detected in wild-type condensin and appeared strongest in a crescent-shaped area of the chromatin mass that stained weakly with the DNA dye DAPI (Figure 1A). This is characteristic of the known enrichment of condensin at the budding yeast rDNA (Freeman et al., 2000). Quantification of the staining intensities, normalized to cellular expression levels, showed that not only ectopic wild-type Smc2 but also all ATPase mutant Smc2 variants associated with chromosomes. Their levels showed some variation but were largely comparable to endogenous Smc2 (Figures 1A and 1B). The same conclusion was reached when we analyzed the raw Smc2 staining intensities (Figure S2B). Thus, an active Smc2 ATPase appears to be dispensable for condensin binding to chromosomes.

We performed a similar analysis for the Smc4 ATPase (Figures S2D–S2F). This again revealed that most Smc4 ATPase mutant proteins bound to DNA at levels comparable to those of wild-type Smc4 (Figures 1C and 1D). As a striking exception, Smc4 K191A, carrying a Walker A motif mutation that is predicted to prevent ATP binding, was undetectable on chromosomes. Chromosome spreads report mainly on condensin that is bound to the rDNA. To extend the analysis to non-rDNA loci and to repeat it using a complementary technique, we examined the chromosomal binding of Smc4 ATPase mutants by chromatin immunoprecipitation, followed by qPCR. We chose the *CEN9* centromeric locus, which is known to be enriched for condensin (D’Ambrosio et al., 2008). This confirmed chromosome association of most Smc4 ATPase mutants, but not the Smc4 K191A mutant, which was detected at levels close to that of a negative control (Figure 1E). Binding of the Smc4 ATP hydrolysis mutants E1352Q and E1352D also appeared somewhat reduced, a possible consequence of their reduced expression levels (Figures S2E and S2F). We obtained similar results at three condensin binding sites along chromosome arms, a tRNA gene and two ribosomal protein gene promoters, although condensin was only weakly detected at these sites (Figure S3).

Loss of Smc4 K191A from chromosomes could result from either impaired chromosome binding or defective condensin complex assembly. To distinguish between these possibilities, we tested the integrity of condensin complexes containing ATPase mutant Smc2 and Smc4 subunits. We fused wild-type and ATPase mutant Smc subunits to a Protein A tag for pull-down and analyzed coprecipitation of the Brn1 subunit by western blotting. All ATPase mutant Smc2 subunits coprecipitated Brn1 at levels equal to those of wild-type Smc2 (Figure 1F). Most Smc4 ATPase mutants also efficiently coprecipitated Brn1 (Figure 1G), with the exception of Smc4 K191A, whose interaction with Brn1 was markedly reduced. This suggests that Smc4 ATP binding is required for stable condensin complex assembly. In the case of cohesin, a similar ATP requirement for Smc1 subunit interaction with the Scc1 kleisin was observed (Arumugam et al., 2003, Weitzer et al., 2003). While we do not yet know how the Smc4 (or Smc1) ATP binding site mutation weakens the kleisin interaction, the loss of Smc4 K191A from chromosomes is likely a consequence of its inability to form a stable condensin complex.

Our observation that budding yeast condensin associates with chromosomes independently of a functional ATPase is broadly consistent with results from vertebrate condensin (Hudson et al., 2008, Kinoshita et al., 2015). Our observations differ with respect to Walker A motif mutations. Vertebrate condensin containing both Smc4 and Smc2 Walker A motif mutations also fails to gain stable chromosome binding. However, in contrast to our observations, these mutant Smc subunits appear to be part of a stable condensin complex. The exact consequences of ATP binding on subunit interactions, complex stability, and chromosome recruitment thus remain to be explored. Condensin is thought to be targeted to chromosomes by interactions with transcription factors (Haeusler et al., 2008, Iwasaki et al., 2015). Physical interactions with such targeting components might provide ATPase-independent chromatin recruitment. Direct DNA contacts of condensin’s HEAT repeat subunits could also contribute to its recruitment (Piazza et al., 2014). We expect that subsequent topological loading of condensin onto DNA requires ATP hydrolysis.

### ATP Hydrolysis Controls rDNA Condensation and Segregation

We next assessed the effect of condensin ATPase mutations on chromosome condensation. The rDNA array on the right arm of budding yeast chromosome XII is a condensin-rich, approximately 1 Mb long, well-characterized model locus for chromosome condensation (Freeman et al., 2000, Lavoie et al., 2004, Sullivan et al., 2004). We visualized the locus by fusing the rDNA binding protein Net1 to the yellow fluorescent protein mCitrine (Griesbeck et al., 2001) and used three-dimensional structured illumination microscopy (SIM) to capture high-resolution images of a fluorescent-conjugated GFP nanobody bound to Net1-mCitrine (Ries et al., 2012). We adapted a previously reported method to extract the high-frequency, sub-diffraction information provided by SIM from these images and automatically determined an intensity threshold for each cell (Marbouty et al., 2015; see Supplemental Experimental Procedures for details). This allowed us to count the number of high-density volumetric pixels per cell as a high-resolution measure of chromosome condensation. An exploratory analysis revealed a measurable difference in condensation between G1 and mitotic cells, validating the approach (Figure S4A).

**Figure 2:**
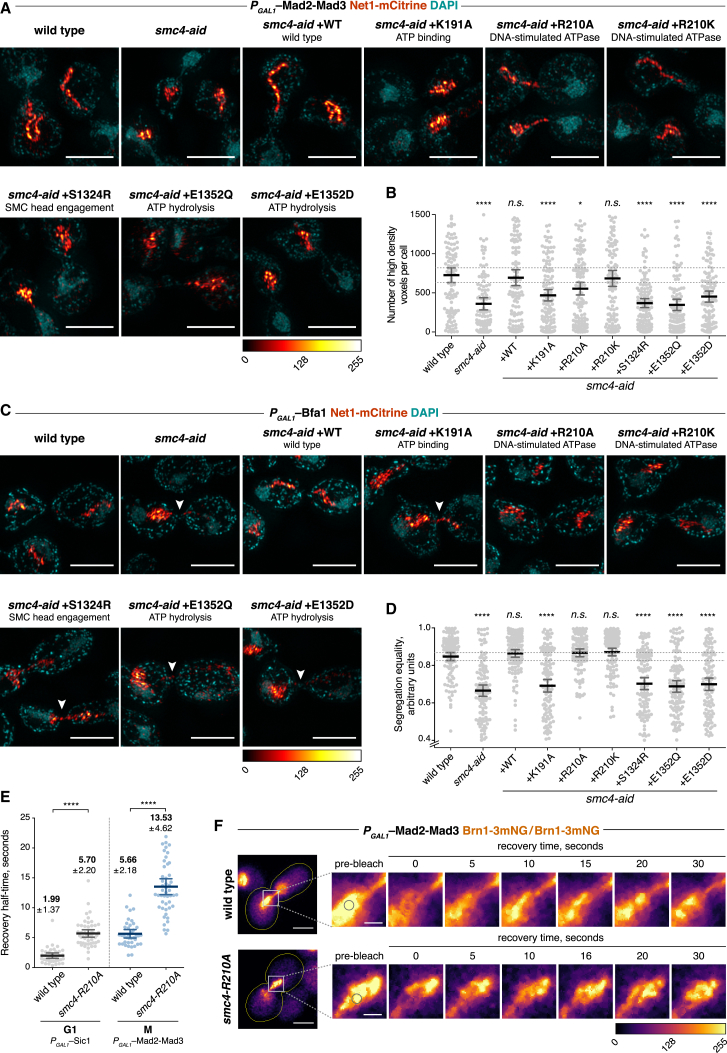
SMC ATPase Controls Chromosome Condensation, Segregation, and Condensin Turnover (A) Metaphase rDNA morphology of Smc4 ATPase mutants visualized by structured illumination microscopy of the rDNA binding protein Net1. Cells were synchronized in G1 with α factor and released into a metaphase arrest due to overexpression of a Mad2-Mad3 fusion protein. Scale bars represent 4 μm. (B) Quantification of rDNA condensation, measured by the number of high-density volumetric pixels in the high-frequency data of structured illumination images. Error bars represent mean ± 95% confidence interval (n ≥ 90); asterisks denote p values with respect to the first column (NS, not significant; ^∗^p < 0.05, ^∗∗^p < 0.01, ^∗∗∗^p < 0.001, ^∗∗∗∗^p < 0.0001; ordinary one-way ANOVA, Dunnett’s multiple comparison test). (C) rDNA segregation in Smc4 ATPase mutants visualized by structured illumination microscopy of the rDNA binding protein Net1. Cells were synchronized in G1 and released into a late anaphase arrest due to overexpression of Bfa1. Arrowheads indicate chromatin bridges. Scale bars represent 4 μm. (D) Quantification of rDNA segregation as the ratio of Net1 fluorescence intensity in the two cell halves. A ratio of 1 indicates equal segregation. Error bars represent mean ± 95% confidence interval (n ≥ 106); asterisks denote p values as in (B). (E) Fluorescence recovery half-times, following photobleaching, of the Brn1-3mNeonGreen signal in homozygous diploid wild-type and *smc4-R210A* cells, arrested in G1 by overexpression of Sic1 or metaphase by overexpression of Mad2-Mad3. Error bars represent mean ± SD (n ≥ 37) (ordinary one-way ANOVA, Sidak’s multiple comparison test). (F) Examples of fluorescence recovery of the Brn1-3mNeonGreen signal in wild-type and *smc4-R210A* cells (outlined) in metaphase, with the area around the bleach spot (indicated by circles) magnified for clarity. Scale bars for whole-cell images represent 4 μm; those for the inset represent 1 μm. See also Figure S4 for illustration of the high-density voxel chromosome condensation assay and confirmation of the cell-cycle arrests.

Following release from synchronization with pheromone α factor treatment in G1, we overexpressed Mad2-Mad3, a fusion protein of two mitotic checkpoint proteins, from the galactose-inducible *GAL1* promoter to achieve uniform mitotic arrest (Figure S4B) (Lau and Murray, 2012). Auxin was added at the time of G1 release to deplete endogenous Smc4. This allowed us to assess the ability of ectopic wild-type or ATPase mutant Smc4 to support chromosome condensation. Most Smc4 ATP binding and hydrolysis mutations were unable to support chromosome condensation over what is seen in the absence of Smc4, as evident by the failure to generate high-density Net1 signals in mitosis (Figures 2A and 2B). Only the mild arginine finger R210K mutation supported chromosome condensation and, to a smaller extent, the R210A variant. This confirms that ATP hydrolysis is instrumental for chromosome condensation. The intermediate condensation defect caused by a mild ATPase mutation suggests that an ATP hydrolysis-dependent step might be rate limiting for chromosome condensation.

Besides chromosome condensation, condensin is crucial for sister chromatid resolution. Anaphase bridges and consequent chromosome missegregation are characteristic features of mitosis with compromised condensin function. We therefore assessed the fidelity of rDNA segregation as a measure for condensin function. We synchronized cells in late anaphase by overexpression of the mitotic exit inhibitor Bfa1 (Figure S4C) (Li, 1999), and we recorded rDNA segregation equality as the ratio of Net1-mCitrine signals in the two cell halves (the lower signal divided by the higher). In wild-type cells, the rDNA equally segregated into the two daughter cells (Figures 2C and 2D). Following Smc4 depletion, rDNA bridges often remained visible and unequal segregation was evident. Segregation equality was re-established by expression of wild-type Smc4 and by the two arginine finger mutants R210A and R210K, but not by any other ATPase mutants. These results confirm that condensin’s ATPase is instrumental for its function in chromosome segregation. We note a subtle difference in the effect of individual ATPase mutations on chromosome condensation and sister chromatid segregation. Smc4 R210A was significantly impaired in promoting rDNA condensation but proficient in securing its equal segregation (compare Figures 2B and 2D), which may explain the viability of this ATPase mutant (Figure S1B). This difference is compatible with the idea that chromosome condensation and sister chromatid resolution are separable activities of the condensin complex (D’Amours et al., 2004, Sullivan et al., 2004). We cannot, however, exclude the possibility that condensation is merely delayed in the Smc4 R210A mutant and reaches wild-type levels later in anaphase, allowing equal rDNA segregation.

### Cell-Cycle-Regulated Condensin Dynamics, Controlled by Its ATPase

Having confirmed the importance of the budding yeast condensin ATPase, we asked whether ATP hydrolysis affects the dynamic chromosome binding behavior of condensin. We fused three tandem copies of mNeonGreen (Shaner et al., 2013) to the C terminus of the endogenous Brn1 subunit. This yielded a bright condensin signal in the yeast nucleus that allowed us to perform fluorescence recovery after photobleaching (FRAP) experiments. We used homozygous diploid strains for these experiments, whose larger nuclear area facilitated the fluorescence recordings.

To investigate whether the dynamic turnover of budding yeast condensin on chromosomes changes between interphase and mitosis, we arrested cells in late G1 by overexpression of the Cdk inhibitor Sic1 (Figure S4D) (Lopez-Serra et al., 2013) or in mitosis by overexpression of Mad2-Mad3. Fluorescence recovery of a bleached region in G1 was very fast, with a recovery half-time of 1.99 ± 1.37 s (mean ± SD). In mitosis, the recovery half-time almost tripled to 5.66 ± 2.18 s (Figures 2E and 2F), suggesting reduced turnover of chromosome-bound condensin in mitosis. A mitotic recovery half-time of 5.66 s in our experiments is compatible with a fluorescent decay constant derived from a fluorescence loss in photobleaching experiment of around 8 s (Robellet et al., 2015). Another study reported a markedly lower rate of condensin turnover in mitotic budding yeast cells (Lawrimore et al., 2015). We do not know the reason for this difference. While our measurements revealed an overall faster turnover of yeast condensin compared to human condensin (Gerlich et al., 2006), the relative stabilization of chromosome binding in mitosis appears to be a conserved feature that characterizes condensin function in both organisms.

We next addressed whether chromosome binding dynamics of the condensin complex are controlled by its ATPase. If this was the case, we would expect slower condensin turnover if the ATPase is compromised. We chose to investigate this using Smc4 R210A, which supports cell viability with reduced rDNA condensation. We created a yeast strain in which endogenous Smc4 was replaced with Smc4 R210A and repeated the FRAP experiments to determine condensin turnover. The condensin residence time on chromosomes increased roughly 3-fold, both in interphase and in mitosis (Figures 2E and 2F). This suggests that ATP hydrolysis regulates condensin’s residence time on chromosomes and that an ATP hydrolysis-dependent step, possibly DNA entry or exit from the condensin ring, correlates with its turnover.

### Cell-Cycle Regulation of Condensin Dynamics by Smc4 Phosphorylation

**Figure 3:**
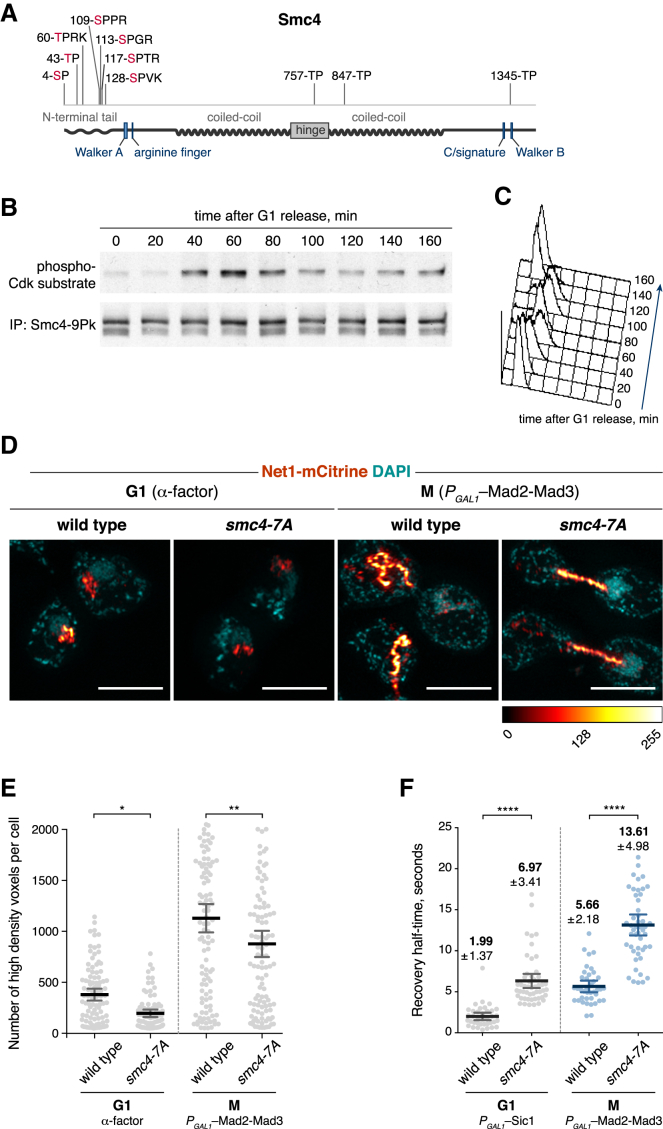
Phosphosite Mutant Smc4 Affects Condensin Dynamics and Chromosome Condensation (A) Schematic representation of Smc4, showing the seven N-terminal Cdk phosphorylation sites. (B) Time course of Smc4 phosphorylation after release from an α factor-induced G1 arrest, assessed by Smc4 immunoprecipitation and probing with an anti-phospho-Cdk substrate antibody. (C) Flow cytometry profiles of DNA content in cells assessed in (B). (D) rDNA morphology of wild-type and *smc4-7A* cells in G1 or metaphase, visualized by structured illumination microscopy of the rDNA binding protein Net1. Cells were synchronized in G1 with α factor and released into a metaphase arrest. Scale bars represent 4 μm. (E) Quantification of rDNA condensation in *smc4-7A* cells compared to wild-type in both G1 and metaphase. The means and 95% confidence intervals are presented (n ≥ 80) (ordinary one-way ANOVA, Sidak’s multiple comparison test). (F) Fluorescence recovery half-times, following photobleaching, of the Brn1-3mNeonGreen signal in homozygous diploid wild-type and *smc4-7A* cells arrested in G1 by overexpression of Sic1 or metaphase by overexpression of Mad2-Mad3. Error bars represent mean ± SD (n ≥ 34) (ordinary one-way ANOVA, Sidak’s multiple comparison test). See also Figure S5 for the identification of Smc4 phosphorylation sites.

The budding yeast Smc4 subunit is a target for Cdk phosphorylation (Robellet et al., 2015). Mass spectrometric analysis of Smc4, immunopurified from mitotically arrested cells, confirmed phosphorylation of a cluster of Cdk consensus sites close to the Smc4 N terminus. These lie within an N-terminal extension that precedes the Smc4 ATPase head domain (Figures 3A and S5). We could not detect phosphorylation of additional Cdk consensus sites within Smc4 that have previously been invoked in condensin regulation (Robellet et al., 2015). Smc4 shows little electrophoretic mobility change during cell-cycle progression. To analyze the timing of Smc4 phosphorylation during the cell cycle, we therefore immunoprecipitated Smc4 from aliquots of a culture that passed through a synchronous cell cycle and probed Smc4 using a phospho-Cdk substrate antibody (Figures 3B and 3C). While Smc4 levels were constant throughout the cell cycle, reactivity of the phospho-Cdk substrate antibody increased following S phase and peaked during mitosis. This suggests that Smc4 undergoes mitosis-specific Cdk phosphorylation in budding yeast.

To test whether Cdk phosphorylation controls the stabilization of condensin on mitotic chromosomes, we initially employed a *smc4-7A* allele in which the 7 Cdk consensus recognition sites in the Smc4 N terminus have been replaced by alanines. As reported (Robellet et al., 2015), *smc4-7A* cells show defective chromosome condensation (Figure 3D). However, rDNA compaction was reduced in *smc4-7A* cells not only in mitosis but also in G1 arrested cells, when Smc4 is not expected to be phosphorylated (Figure 3E). This suggests that the Smc4-7A protein is defective in ways additional to being refractory to Cdk phosphorylation. This conclusion was corroborated when we measured chromosome binding dynamics of condensin. Non-phosphorylatable Smc4-7A would be expected to maintain fast interphase turnover kinetics even in mitosis. In contrast to this expectation, we found that Smc4-7A showed slower recovery times than wild-type condensin, both in interphase and in mitosis (Figure 3F). This suggests that the Smc4 N-terminal extension plays a role in condensin function but that the Smc4-7A phenotype goes beyond being non-phosphorylatable. In addition, Smc4-7A condensin turnover retained aspects of cell-cycle regulation, being more stable on chromosomes in mitosis than in interphase. Thus, levels of mitotic condensin regulation exist that go beyond Cdk phosphorylation of Smc4.

**Figure 4:**
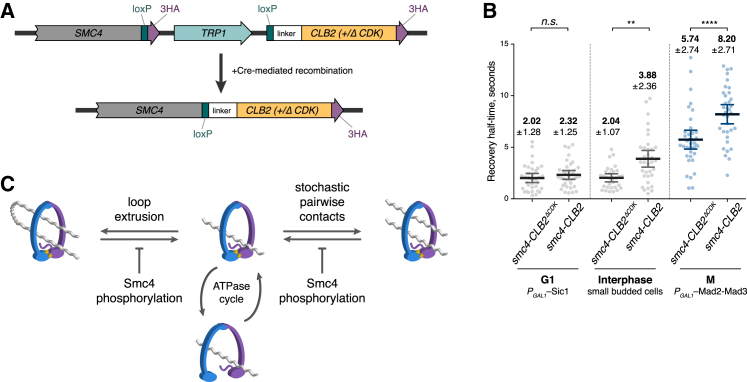
Smc4 Phosphorylation Regulates Condensin Dynamics and Chromosome Condensation (A) Schematic representation of the gene loci expressing Smc4-Clb2 and Smc4-Clb2^ΔCdk^ fusion proteins. (B) Fluorescence recovery half-times, following photobleaching, of the Brn1-3mNeonGreen signal in homozygous diploid *SMC4-CLB2*^Δ*CDK*^ and *SMC4-CLB2* cells in interphase and metaphase. Error bars represent mean ± SD (n ≥ 31) (ordinary one-way ANOVA, Sidak’s multiple comparison test). (C) Model of chromosome condensation driven by cell-cycle-regulated Smc4 phosphorylation, resulting in stabilization of condensin-DNA binding. This promotes chromosome compaction if the condensation reaction proceeds by either diffusion capture or loop extrusion. See also Figure S6 for additional condensin dynamics, phosphorylation, and chromosome condensation assays.

Given the difficulty with the interpretation of results obtained with the *smc4-7A* allele, we aimed to create a gain-of-function *SMC4* allele that is phosphorylated prematurely. We fused Smc4 to the mitotic cyclin Clb2 (Smc4-Clb2), with the expectation that this recruits Cdk activity to Smc4 and leads to its phosphorylation even in interphase. Clb2 was stripped of localization and degradation signals to avoid unwanted interference with Smc4 function. This approach was previously successful with achieving constitutive phosphorylation of several other mitotic regulators (Kuilman et al., 2015). As a control, we generated a fusion of Smc4 with a Clb2 variant that is defective in recruiting Cdk (Smc4-Clb2^ΔCdk^) (Figure 4A). We arrested cells in G1 with the expectation that condensin turnover should be reduced in Smc4-Clb2 cells if Smc4 phosphorylation regulates condensin turnover. The recovery half-life of the Smc4-Clb2^ΔCdk^ condensin fusion was close to that of wild-type condensin, confirming that the fusion construct did not interfere with normal condensin function. However, the increase in recovery half-time of the Smc4-Clb2 condensin fusion was only marginal and not significant (Figure 4B). We surmised that overexpression of the Cdk inhibitor Sic1, which we used to produce a G1 arrest in our diploid cells, might compromise the ability of the Clb2 fusion to phosphorylate Smc4. Alternatively, the G1 arrest state might be unconducive to chromosome condensation for additional reasons.

To overcome the need for a G1 arrest, we analyzed condensin dynamics in an asynchronously growing cell population. We selected interphase cells for experiments based on their small bud size (in a post-experiment analysis, the ratio of daughter-to-mother cell area was 0.15 ± 0.04, mean ± SD). This includes cells in late G1, S, and early G2. The recovery half-time of the Smc4-Clb2^ΔCdk^ condensin fusion remained close to what we previously observed in G1 arrested cells, suggesting that little change to condensin dynamics occurs while cells progress through interphase. In contrast, the recovery half-time of the Smc4-Clb2 condensin fusion almost doubled, suggesting that Cdk phosphorylation contributes to a slowdown of condensin turnover on chromosomes (Figure 4B). The recovery half-time of Smc4-Clb2 in interphase was less than that observed in mitosis, consistent with the possibility that regulation, in addition to Smc4 phosphorylation, affects condensin in mitosis. This might, for example, include phosphorylation by Polo kinase (St-Pierre et al., 2009), which is not fully active in interphase. In mitotically arrested cells, Smc4-Clb2 also turned over more slowly than Smc4-Clb2^ΔCdk^, which could be explained if Smc4 is incompletely phosphorylated during normal mitosis and additional phosphorylation due to the Smc4-Clb2 fusion is able to further dampen condensin turnover. As a control so that the effect of Clb2 fusion is mediated by Smc4 phosphorylation, we also fused Clb2 to Smc4-7A. While Smc4-7A condensin turns over more slowly, its behavior remained unchanged in response to Clb2 fusion (Figure S6A). This supports the idea that the Clb2 fusion exerts its effect by N-terminal Smc4 phosphorylation and therefore that Smc4 phosphorylation contributes to regulating condensin turnover on chromosomes.

We confirmed that Smc4-Clb2 fusion led to increased Smc4 phosphorylation by probing immunoprecipitated Smc4 and Smc4-Clb2 with a phospho-Cdk substrate antibody (Figure S6B). We observed increased reactivity with the antibody following Clb2 fusion, suggestive of increased phosphorylation. Clb2 fusion also led to a greater phosphorylation signal in G1 arrested cells and of the Smc4-7A protein, albeit to a lesser extent compared to wild-type Smc4 in mitosis. This suggests that Clb2 fusion leads to phosphorylation of additional sites on Smc4-7A, while functionally important sites lie within the Smc4 N terminus.

### Chromosome Condensation Requires Tuned Condensin Turnover

Finally, we addressed whether reduced condensin turnover following Smc4-Clb2 fusion leads to premature chromosome condensation. To analyze this, we arrested haploid cells in G1 by α factor treatment. Smc4-Clb2 caused a marginal, but not statistically significant, increase in rDNA compaction (Figures S6C and S6D). When we arrested cells in mitosis, we found that Clb2 fusion compromised rDNA condensation. As a control, Clb2^ΔCdk^ fusion did not compromise condensation, excluding a non-specific effect of the fusions. While somewhat counter-intuitive at first glance, this observation is reminiscent of Smc4 R210A cells in which reduced condensin turnover led to compromised chromosome condensation rather than hypercompaction. Thus, slowing condensin turnover beyond its normally observed rates impairs rather than increases chromosome condensation.

## Discussion

We have shown that dynamic condensin binding to chromosomes is controlled by the condensin ATPase and by cell-cycle-dependent phosphorylation. Mitotic chromosome condensation correlates with a slowdown of condensin turnover, mediated by mitotic phosphorylation. However, reduction of turnover by hyperphosphorylation or an ATPase mutation does not increase condensation. Rather, our observations suggest that chromosome compaction requires an optimum rate of condensin turnover. We imagine that at this rate, intra-chromosomal contacts are established at a sufficient rate and maintained for an adequate length of time.

The idea that chromosome condensation requires an optimum condensin turnover rate can apply to either of two proposed chromosome compaction models: diffusion capture or loop extrusion (Alipour and Marko, 2012, Cheng et al., 2015, Fudenberg et al., 2016). The first model proposes that condensin stabilizes stochastic encounters between its binding sites, either by trapping more than one DNA within one condensin ring or by interactions between more than one condensin. Here, the establishment of productive DNA-DNA interactions likely requires multiple rounds of ATP hydrolysis to entrap more than one DNA duplex in the condensin ring, followed by a sufficiently long retention period. If both DNA entry and DNA exit are ATP hydrolysis-dependent reactions, as is the case for the cohesin ring (Murayama and Uhlmann, 2015), a fine balance between DNA entry and retention must be achieved. The alternative model of loop extrusion proposes that compaction proceeds by threading a DNA loop through condensin and enlarging it. In this case, ATP hydrolysis may drive both DNA entry into the condensin ring and subsequent DNA translocation (Ganji et al., 2018). A balance between loop initiation and the processivity of extrusion must be found to reach compaction.

Cdk phosphorylation in both budding and fission yeast targets an N-terminal Smc4 extension. In fission yeast, this increases the nuclear import of condensin (Sutani et al., 1999). Whether it also changes the dynamic behavior of fission yeast condensin is not yet known. In vertebrates, mitotic Cdk phosphorylation of condensin I and II occurs on C-terminal parts of the CAP-D2 and CAP-D3 subunits, respectively (Abe et al., 2011, Kimura et al., 1998). If HEAT repeat subunit topology is similar between cohesin and condensin (Lee et al., 2016), these regions might lie close to the SMC4 N terminus. In this way, Cdk phosphorylation could in all cases add negative charge to a related part of the condensin complex, paving the way for a conserved mechanism of control. Phosphorylation could lead to electrostatic repulsion of DNA, potentially altering how DNA engages with the complex. Alternatively, phosphorylation could induce conformational changes that alter condensin behavior. Further biochemical investigations will be required to understand how condensin promotes DNA condensation and how control of its dynamic behavior by posttranslational modifications regulates this essential cell biological process.

## Experimental Procedures

Additional details are available in the Supplemental Experimental Procedures.

### Chromosome Spreads

We prepared chromosome spreads as previously described (Loidl et al., 1991) but adapted the procedure for multiwell slides. We resuspended 3 × 10^7^ cells in 1 mL of S1 (100 mM potassium phosphate buffer [pH 7.4], 0.5 mM MgCl_2_, 1.2 M sorbitol). Cells were spheroplasted by incubation at 37°C for 20 min in S1 containing 20 mM DTT and 140 μg/mL of zymolase T-100. We halted cell wall digestion by addition of 1 mL of ice-cold S3 (0.1 M 2-(N-morpholino)ethanesulfonic acid, 1 mM EDTA, 0.5 mM MgCl_2_, 1 M sorbitol [pH 6.4]) and resuspended washed spheroplasts in 200 μL of S3. For spreading, we rapidly pipetted onto each chamber of a multiwell slide 2 μL of fixative (4% paraformaldehyde, 3.4% sucrose, 0.2 mM NaOH), 2 μL of spheroplast suspension, 4 μL of 1% lipsol in water, and 4 μL of fixative. Slides were dried overnight before immunostaining (rat anti-hemagglutinin [anti-HA] 3F10, 1:500; Alexa Fluor 594 anti-rat, 1:1,000), followed by DAPI staining and SIM.

### Cell Fixation and Nanobody Staining

We fixed ∼3 × 10^8^ cells arrested in G1 (α factor), metaphase (*P*_*GAL1*_-Mad2-Mad3), or anaphase (*P*_*GAL1*_-Bfa1) by addition of 3.6% methanol-free, electron microscopy (EM)-grade formaldehyde to the culture medium. After incubation at room temperature for 15 min, we halted fixation by washing cells in TBS (150 mM NaCl, 50 mM Tris-HCl [pH 8.0]) containing 50 mM NH_4_Cl and then 3× with TBS. Cells were stained with Atto 594-conjugated GFP booster nanobody at 4°C overnight and DAPI and then imaged by SIM using an API OMX v3 microscope.

### FRAP

Brn1-3mNeonGreen diploid cells were grown in rich yeast peptone (YP) medium supplemented with 2% raffinose. Cell-cycle arrests were performed for 3–4.5 hr by addition of 2% galactose directly to asynchronous cultures. Cells were then harvested and resuspended in synthetic yeast nitrogen base (YNB) medium supplemented with 2% raffinose + 2% galactose. Cells were then applied to a 1.2% agarose-medium pad for imaging. FRAP experiments were performed on a Zeiss LSM 880 confocal microscope with 488 nm laser excitation and >505 nm longpass emission settings. We typically acquired 3 pre-bleach frames at 0.5%–1% power, bleached a circular spot of 9 pixel/0.59 μm diameter with 5 iterations at 50% power, and monitored recovery every 1–3 s at 0.5%–1% power. We used fluorescent regions from adjoining cells in the same field to correct for general photobleaching and used Zen software (Zeiss) to fit a single exponential recovery curve. Double exponential curves did not improve the fit.

### Statistical Methods

Statistical analyses were performed in GraphPad Prism. We used an ordinary one-way ANOVA with Dunnett’s test to compare multiple samples to a single control or the Sidak method to compare selected sets of means. For single comparisons, we used Student’s unpaired t test with equal SD.

## Supplemental Information

Document S1. Supplemental Experimental Procedures, Figures S1–S6, and Table S1

Document S2. Article plus Supplemental Information

## References

[bib1] Abe S., Nagasaka K., Hirayama Y., Kozuka-Hata H., Oyama M., Aoyagi Y., Obuse C., Hirota T. (2011). The initial phase of chromosome condensation requires Cdk1-mediated phosphorylation of the CAP-D3 subunit of condensin II. Genes Dev..

[bib2] Alipour E., Marko J.F. (2012). Self-organization of domain structures by DNA-loop-extruding enzymes. Nucleic Acids Res..

[bib3] Arumugam P., Gruber S., Tanaka K., Haering C.H., Mechtler K., Nasmyth K. (2003). ATP hydrolysis is required for cohesin’s association with chromosomes. Curr. Biol..

[bib4] Cheng T.M.K., Heeger S., Chaleil R.A.G., Matthews N., Stewart A., Wright J., Lim C., Bates P.A., Uhlmann F. (2015). A simple biophysical model emulates budding yeast chromosome condensation. eLife.

[bib5] D’Ambrosio C., Schmidt C.K., Katou Y., Kelly G., Itoh T., Shirahige K., Uhlmann F. (2008). Identification of cis-acting sites for condensin loading onto budding yeast chromosomes. Genes Dev..

[bib6] D’Amours D., Stegmeier F., Amon A. (2004). Cdc14 and condensin control the dissolution of cohesin-independent chromosome linkages at repeated DNA. Cell.

[bib7] Freeman L., Aragon-Alcaide L., Strunnikov A. (2000). The condensin complex governs chromosome condensation and mitotic transmission of rDNA. J. Cell Biol..

[bib8] Fudenberg G., Imakaev M., Lu C., Goloborodko A., Abdennur N., Mirny L.A. (2016). Formation of chromosomal domains by loop extrusion. Cell Rep..

[bib9] Ganji M., Shaltiel I.A., Bisht S., Kim E., Kalichava A., Haering C.H., Dekker C. (2018). Real-time imaging of DNA loop extrusion by condensin. Science.

[bib10] Gerlich D., Hirota T., Koch B., Peters J.-M., Ellenberg J. (2006). Condensin I stabilizes chromosomes mechanically through a dynamic interaction in live cells. Curr. Biol..

[bib11] Griesbeck O., Baird G.S., Campbell R.E., Zacharias D.A., Tsien R.Y. (2001). Reducing the environmental sensitivity of yellow fluorescent protein. Mechanism and applications. J. Biol. Chem..

[bib12] Haeusler R.A., Pratt-Hyatt M., Good P.D., Gipson T.A., Engelke D.R. (2008). Clustering of yeast tRNA genes is mediated by specific association of condensin with tRNA gene transcription complexes. Genes Dev..

[bib13] Hirano T. (2016). Condensin-based chromosome organization from bacteria to vertebrates. Cell.

[bib14] Hopfner K.-P., Karcher A., Shin D.S., Craig L., Arthur L.M., Carney J.P., Tainer J.A. (2000). Structural biology of Rad50 ATPase: ATP-driven conformational control in DNA double-strand break repair and the ABC-ATPase superfamily. Cell.

[bib15] Hudson D.F., Ohta S., Freisinger T., Macisaac F., Sennels L., Alves F., Lai F., Kerr A., Rappsilber J., Earnshaw W.C. (2008). Molecular and genetic analysis of condensin function in vertebrate cells. Mol. Biol. Cell.

[bib16] Iwasaki O., Tanizawa H., Kim K.-D., Yokoyama Y., Corcoran C.J., Tanaka A., Skordalakes E., Showe L.C., Noma K. (2015). Interaction between TBP and condensin drives the organization and faithful segregation of mitotic chromosomes. Mol. Cell.

[bib17] Kimura K., Hirano T. (1997). ATP-dependent positive supercoiling of DNA by 13S condensin: a biochemical implication for chromosome condensation. Cell.

[bib18] Kimura K., Hirano M., Kobayashi R., Hirano T. (1998). Phosphorylation and activation of 13S condensin by Cdc2 *in vitro*. Science.

[bib19] Kinoshita K., Kobayashi T.J., Hirano T. (2015). Balancing acts of two HEAT subunits of condensin I support dynamic assembly of chromosome axes. Dev. Cell.

[bib20] Kubota T., Nishimura K., Kanemaki M.T., Donaldson A.D. (2013). The Elg1 replication factor C-like complex functions in PCNA unloading during DNA replication. Mol. Cell.

[bib21] Kuilman T., Maiolica A., Godfrey M., Scheidel N., Aebersold R., Uhlmann F. (2015). Identification of Cdk targets that control cytokinesis. EMBO J..

[bib22] Lammens A., Schele A., Hopfner K.-P. (2004). Structural biochemistry of ATP-driven dimerization and DNA-stimulated activation of SMC ATPases. Curr. Biol..

[bib23] Lau D.T., Murray A.W. (2012). Mad2 and Mad3 cooperate to arrest budding yeast in mitosis. Curr. Biol..

[bib24] Lavoie B.D., Hogan E., Koshland D. (2004). *In vivo* requirements for rDNA chromosome condensation reveal two cell-cycle-regulated pathways for mitotic chromosome folding. Genes Dev..

[bib25] Lawrimore J., Vasquez P.A., Falvo M.R., Taylor R.M., Vicci L., Yeh E., Forest M.G., Bloom K. (2015). DNA loops generate intracentromere tension in mitosis. J. Cell Biol..

[bib26] Lee B.-G., Roig M.B., Jansma M., Petela N., Metson J., Nasmyth K., Löwe J. (2016). Crystal structure of the cohesin gatekeeper Pds5 and in complex with kleisin Scc1. Cell Rep..

[bib27] Lengronne A., McIntyre J., Katou Y., Kanoh Y., Hopfner K.-P., Shirahige K., Uhlmann F. (2006). Establishment of sister chromatid cohesion at the *S. cerevisiae* replication fork. Mol. Cell.

[bib28] Li R. (1999). Bifurcation of the mitotic checkpoint pathway in budding yeast. Proc. Natl. Acad. Sci. USA.

[bib29] Loidl J., Nairz K., Klein F. (1991). Meiotic chromosome synapsis in a haploid yeast. Chromosoma.

[bib30] Lopez-Serra L., Lengronne A., Borges V., Kelly G., Uhlmann F. (2013). Budding yeast Wapl controls sister chromatid cohesion maintenance and chromosome condensation. Curr. Biol..

[bib31] Marbouty M., Le Gall A., Cattoni D.I., Cournac A., Koh A., Fiche J.B., Mozziconacci J., Murray H., Koszul R., Nollmann M. (2015). Condensin- and replication-mediated bacterial chromosome folding and origin condensation revealed by Hi-C and super-resolution imaging. Mol. Cell.

[bib32] Murayama Y., Uhlmann F. (2015). DNA entry into and exit out of the cohesin ring by an interlocking gate mechanism. Cell.

[bib33] Onn I., Aono N., Hirano M., Hirano T. (2007). Reconstitution and subunit geometry of human condensin complexes. EMBO J..

[bib34] Piazza I., Rutkowska A., Ori A., Walczak M., Metz J., Pelechano V., Beck M., Haering C.H. (2014). Association of condensin with chromosomes depends on DNA binding by its HEAT-repeat subunits. Nat. Struct. Mol. Biol..

[bib35] Ries J., Kaplan C., Platonova E., Eghlidi H., Ewers H. (2012). A simple, versatile method for GFP-based super-resolution microscopy via nanobodies. Nat. Methods.

[bib36] Robellet X., Thattikota Y., Wang F., Wee T.-L., Pascariu M., Shankar S., Bonneil É., Brown C.M., D’Amours D. (2015). A high-sensitivity phospho-switch triggered by Cdk1 governs chromosome morphogenesis during cell division. Genes Dev..

[bib37] Shaner N.C., Lambert G.G., Chammas A., Ni Y., Cranfill P.J., Baird M.A., Sell B.R., Allen J.R., Day R.N., Israelsson M. (2013). A bright monomeric green fluorescent protein derived from *Branchiostoma lanceolatum*. Nat. Methods.

[bib38] St-Pierre J., Douziech M., Bazile F., Pascariu M., Bonneil E., Sauvé V., Ratsima H., D’Amours D. (2009). Polo kinase regulates mitotic chromosome condensation by hyperactivation of condensin DNA supercoiling activity. Mol. Cell.

[bib39] Stray J.E., Lindsley J.E. (2003). Biochemical analysis of the yeast condensin Smc2/4 complex: an ATPase that promotes knotting of circular DNA. J. Biol. Chem..

[bib40] Sullivan M., Higuchi T., Katis V.L., Uhlmann F. (2004). Cdc14 phosphatase induces rDNA condensation and resolves cohesin-independent cohesion during budding yeast anaphase. Cell.

[bib41] Sutani T., Yuasa T., Tomonaga T., Dohmae N., Takio K., Yanagida M. (1999). Fission yeast condensin complex: essential roles of non-SMC subunits for condensation and Cdc2 phosphorylation of Cut3/SMC4. Genes Dev..

[bib42] Thadani R., Uhlmann F., Heeger S. (2012). Condensin, chromatin crossbarring and chromosome condensation. Curr. Biol..

[bib43] Uhlmann F. (2016). SMC complexes: from DNA to chromosomes. Nat. Rev. Mol. Cell Biol..

[bib44] Weitzer S., Lehane C., Uhlmann F. (2003). A model for ATP hydrolysis-dependent binding of cohesin to DNA. Curr. Biol..

