# Supplementary material for: Cell-Cycle Regulation of Dynamic Chromosome Association of the Condensin Complex
Source: Cell Rep. 2018 May 22;23(8):2308–17. doi: 10.1016/j.celrep.2018.04.082 (PMC5986713; doi:10.1016/j.celrep.2018.04.082)
Supplement: Document S1. Supplemental Experimental Procedures, Figures S1–S6, and Table S1 [file mmc1.pdf]

Cell Reports, Volume *23*

## Supplemental Information

# Cell-Cycle Regulation of Dynamic Chromosome Association of the Condensin Complex

**Rahul Thadani, Julia Kamenz, Sebastian Heeger, Sofía Muñoz, and Frank Uhlmann**

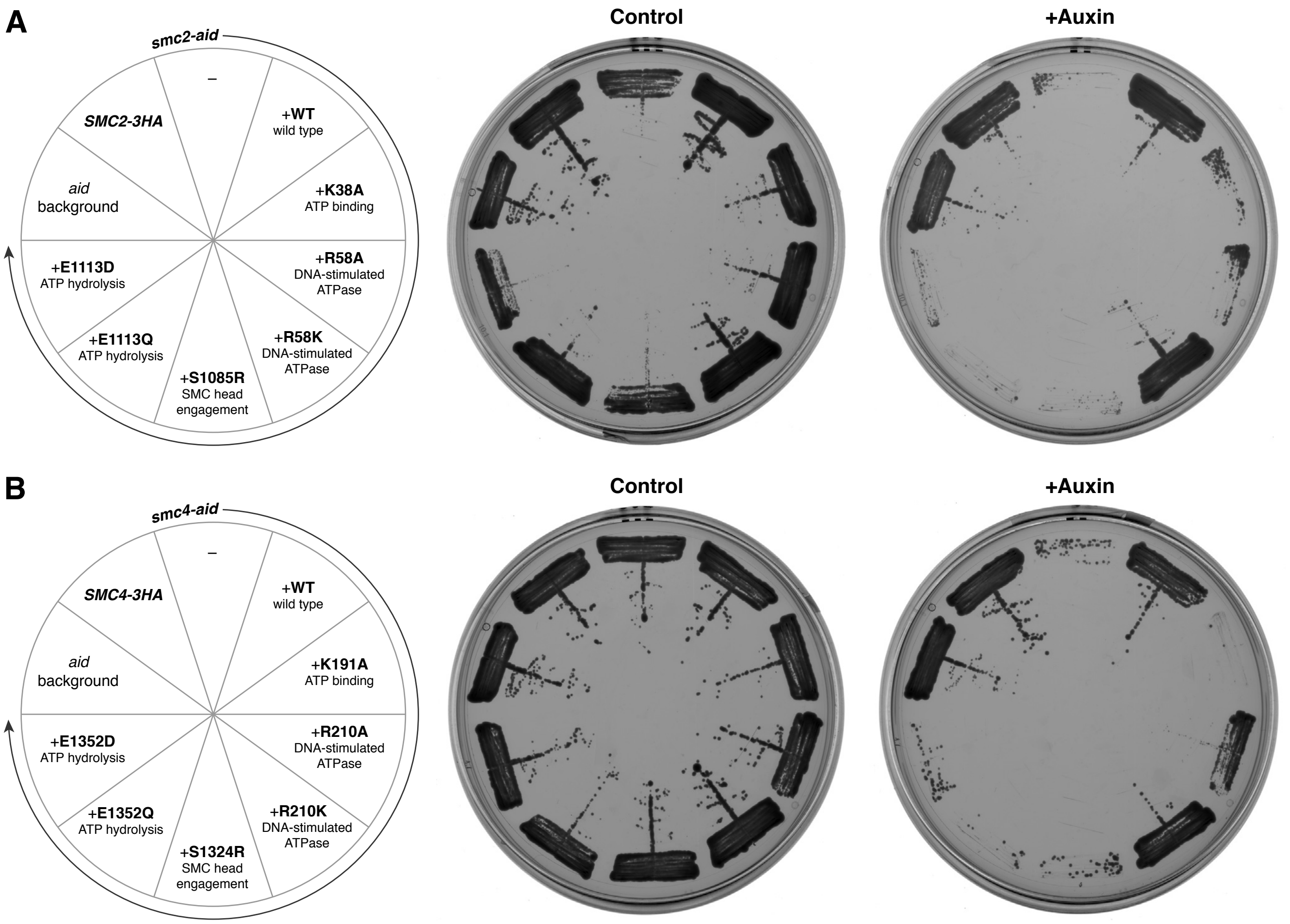


**Figure S1. Viability of Condensin SMC ATPase Mutants, Related to Figure 1**

(A) Strains expressing epitope-tagged ATPase mutants of Smc2 from an ectopic locus, in an *smc2-aid* background, were streaked onto rich medium containing either methanol as control, or auxin (1 mM IAA). A wild type strain expressing epitope-tagged endogenous Smc2 and the Tir1-expressing *aid* background strain without the *smc2-aid* fusion served as controls. Only wild type Smc2 and Smc2 R58K rescue the conditional lethality of *smc2-aid*.

(B) Strains expressing epitope-tagged ATPase mutants of Smc4 from an ectopic locus, in an *smc4-aid* background, were streaked onto rich medium containing either methanol as control, or auxin (1 mM IAA). A wild type strain expressing epitope-tagged endogenous Smc4 and the Tir1-expressing *aid* background strain without the *smc4-aid* fusion served as controls. Wild type Smc4, Smc4 R210A and Smc4 R210K rescue the conditional lethality of *smc4-aid*.

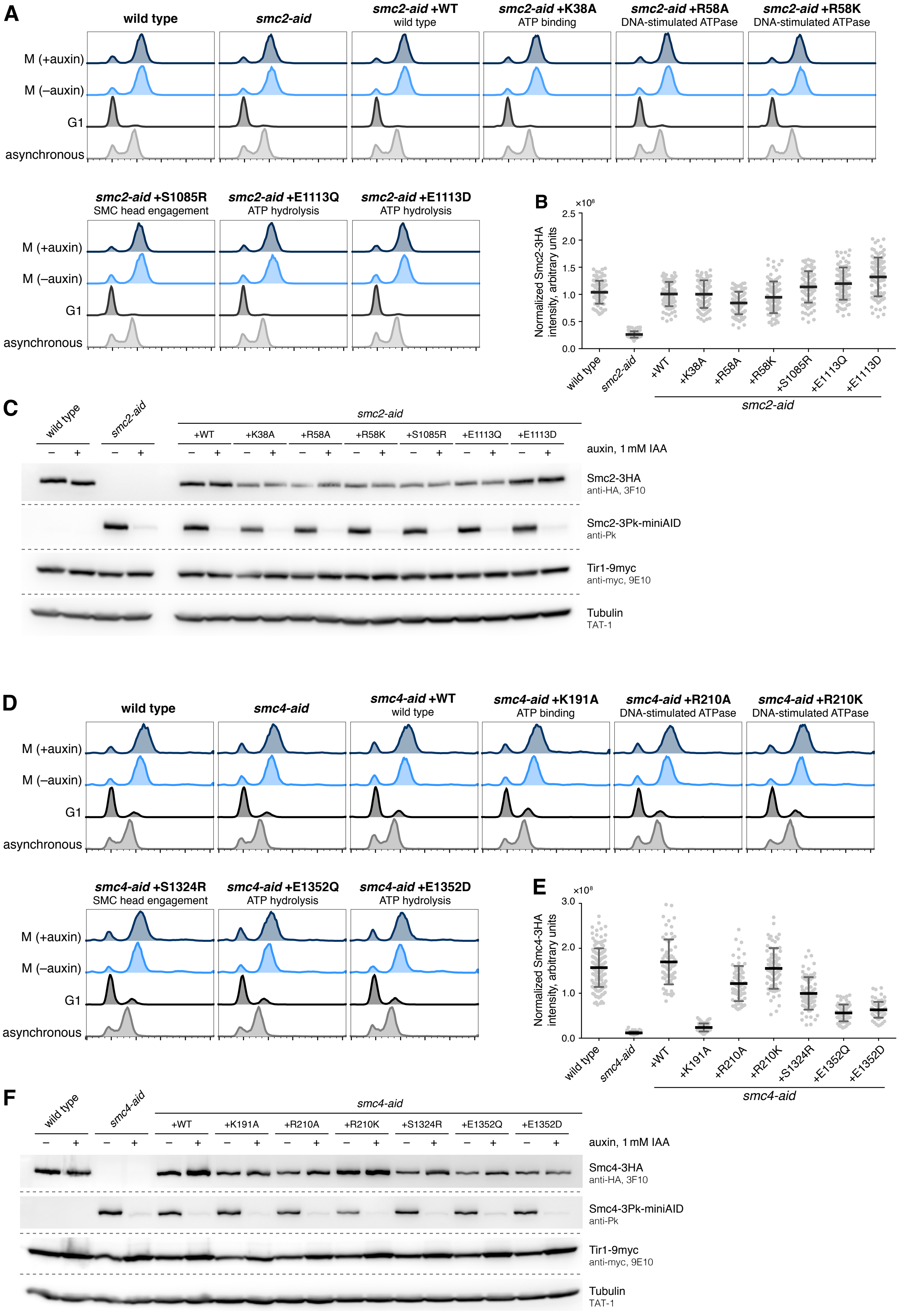
A
wild type
smc2-aid
smc2-aid +WT
wild type
smc2-aid +K38A
ATP binding
smc2-aid +R58A
DNA-stimulated ATPase
smc2-aid +R58K
DNA-stimulated ATPase
M (+auxin)
M (–auxin)
G1
asynchronous
smc2-aid +S1085R
SMC head engagement
smc2-aid +E1113Q
ATP hydrolysis
smc2-aid +E1113D
ATP hydrolysis
B
Normalized Smc2-3HA intensity, arbitrary units
×10⁸
wild type
smc2-aid
+WT
+K38A
+R58A
+R58K
+S1085R
+E1113Q
+E1113D
C
auxin, 1 mM IAA
Smc2-3HA
anti-HA, 3F10
Smc2-3Pk-miniAID
anti-Pk
Tir1-9myc
anti-myc, 9E10
Tubulin
TAT-1
D
wild type
smc4-aid
smc4-aid +WT
wild type
smc4-aid +K191A
ATP binding
smc4-aid +R210A
DNA-stimulated ATPase
smc4-aid +R210K
DNA-stimulated ATPase
smc4-aid +S1324R
SMC head engagement
smc4-aid +E1352Q
ATP hydrolysis
smc4-aid +E1352D
ATP hydrolysis
E
Normalized Smc4-3HA intensity, arbitrary units
+K191A
+R210A
+R210K
+S1324R
+E1352Q
+E1352D
F
Smc4-3HA
anti-HA, 3F10
Smc4-3Pk-miniAID
anti-Pk
Tir1-9myc
anti-myc, 9E10
Tubulin
TAT-1

**Figure S2. Cell Cycle Synchronization and Expression Levels of SMC ATPase Mutants, Related to Figure 1**

(A) Flow cytometry profiles of control and Smc2 ATPase mutant strains synchronized in G1 with pheromone α-factor and released into a mitotic arrest (4 µg/ml nocodazole) in the absence (methanol) or presence (1 mM IAA) of auxin.

(B) Raw Smc2-3HA staining intensities from chromosome spreads shown in Figure 1A. Compare to normalized intensities in Figure 1B. Error bars represent mean ± s.d. ($n \geq 92$).

(C) Western blots from the experiment described in (A), showing auxin-induced depletion of Smc2-3Pk-miniAID and expression levels of the 3HA-tagged Smc2 ATPase mutants. Tir1 and tubulin served as loading controls.

(D) Flow cytometry profiles of control and Smc4 ATPase mutant strains treated as in (A).

(E) Raw Smc4-3HA staining intensities from chromosome spreads shown in Figure 1C. Compare to normalized intensities in Figure 1D. Error bars represent mean ± s.d. ($n \geq 63$).

(F) Western blots from the experiment in (D), showing auxin-induced depletion of Smc4-3Pk-miniAID and expression levels of the 3HA-tagged Smc4 ATPase mutants.

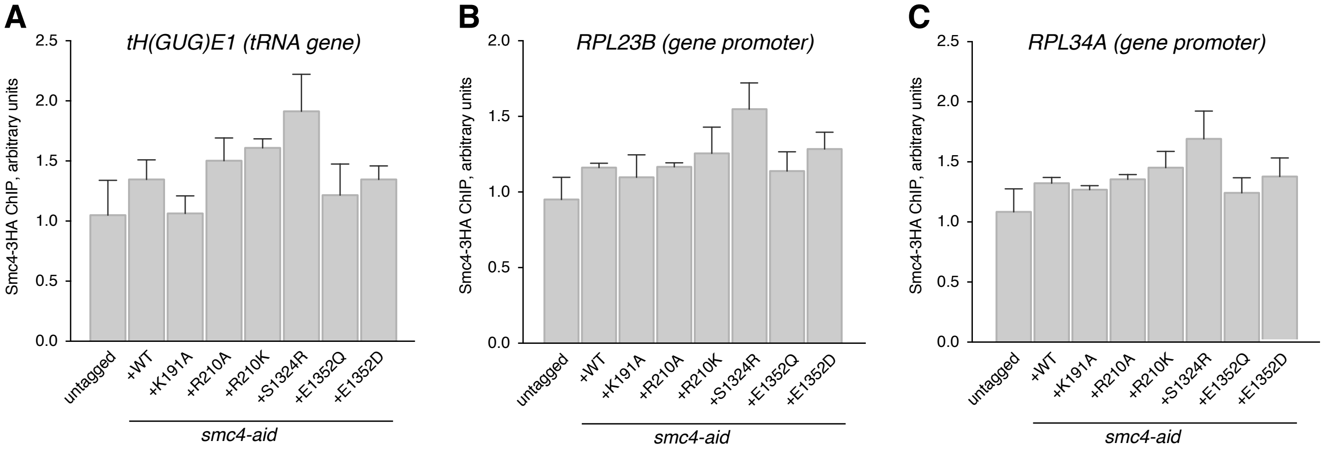


**Figure S3. ChIP-qPCR of Chromosome Arm Loci, Related to Figure 1**

(A) ChIP-qPCR signal of Smc4-3HA at the *tH(GUG)E1* tRNA gene locus, normalized to a negative binding site, in *smc4-aid* cells in metaphase expressing ectopic wild type or ATPase mutants of Smc4. Error bars represent mean ± s.e.m. (n = 3).

(B) ChIP-qPCR signal of Smc4-3HA at the *RPL23B* ribosomal protein gene promoter, as in (A).

(C) ChIP-qPCR signal of Smc4-3HA at the *RPL34A* ribosomal protein gene promoter, as in (A).

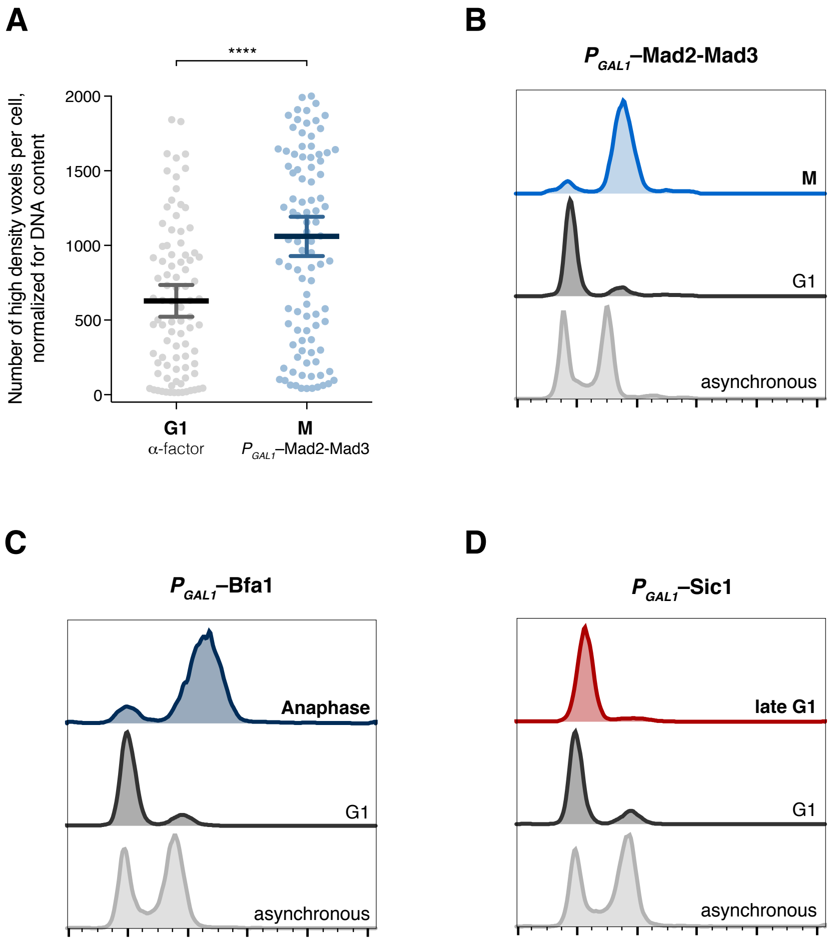


**Figure S4. Chromosome Condensation Assay and Illustrative Cell Cycle Arrests, Related to Figure 2**

(A) Quantification of the number of high density volumetric pixels (derived as described in the Supplemental Experimental Procedures) in cells arrested in G1 or metaphase (M). The significantly increased number in metaphase illustrates the measurable difference in chromosome compaction between the two cell cycle phases, even when normalized for DNA content. Error bars show the mean and 95% confidence interval ($n \geq 88$, **** $p < 0.0001$; unpaired t-test with equal s.d.).

(B) Overexpression of a Mad2-Mad3 fusion protein under control of the *GAL1* promoter results in a mitotic cell cycle arrest with 2C DNA content.

(C) Overexpression of Bfa1 under control of the *GAL1* promoter results in a late mitotic cell cycle arrest with 2C DNA content.

(D) Overexpression of Sic1 under the control of the *GAL1* promoter results in a cell cycle arrest in late G1 with 1C DNA content.

MSDSPLSKRQKR**KAAQEPELSLDQGDAEEDSQVENRVNLSENTPEPDLPALEASYSKSYTPR**K**LVLSSGENRYAFSQPTN**
**STTTSLHVPNLQPPK**TSSRGRDHK**SYSQSPPRSPGR**SPTR**RLELLQLSPVK**NSRVELQKIYDSHQSSSKQQSR**LFINELV**
**LENFK**SYAGK**QVVGPFHTSFSAVVGPNGSGKSNVIDSMLFVFGFR**ANKMRQDR**LSDLIHKSEAFPSLQSCSVAVHFQYVI**
**DESSGTSRIDEEKPGLIITR**KAFKNNSSKYYINEK**ESSYTEVTKLLKNEGIDLDHKRFLILQGEVENIAQMKPK**AEKESF
**DDGLLEYLEDIIGTANYKPLIEERMGQIENLNEVCLEKENREIVDR**EK**NSLESGKETALEFLEK**EKQLTLLRSKLFQFKL
LQSNSK**LASTLEKISSSNKDLEDERMKFQ**ESLKKVDEIKAQRKEIKDRISSCSSKEK**TLVLER**RELEGTRVSLEERTK**NL**
**VSKM**EKAEKTLKSTK**HSISEAENMLEELRGQQTEHETEIKDLTQLLEK**ER**SILDDIKLSLK**DK**TKDISAEIIR**HEK**ELEP**
**WDLQLQEKESQIQLAESELSLLEETQAK**LKK**NVETLEEK**ILAKKTHK**QELQDLILDLKKK**LNSLKDERSQGEKNFTSAHL
KLKEMQKVLNAHRQRAMEARSSLSKAQNKSKVLTALSRLQKSGRINGFHGRLGDLGAIDDSF**DIAISTACPRLDDVVVDT**
**VECAQHCIDYLR**KNKLGYAR**FILLDR**LR**QFNLQPISTPENVPRLFDLVKPK**NPK**FSNAFYSVLRDTLVAQNLKQANNVAY**
**GK**KR**FRVVTVDGKLIDISGTMSGGGNHVAKGLMKLGTNQSDKVDDYTPEEVDKIER**ELSERENNFR**VASDTVHEMEEELK**
**KLRDHEPDLESQISKAEMEADSLASELTLAEQQVKEAEMAYVKAVSDKAQLNVVMK**NLER**LRGEYNDLQSETK**TKKEKIK
**GLQDEIMK**IGGIKLQMQNSKVESVCQK**LDILVAK**LKKVKSASKKSGGDVVKFQK**LLQNSERDVELSSNELK**VIEEQLKHT
K**LALAENDTNMNETLNLK**VELK**EQSEQLKEQMEDMEESINEFK**SIEIEMKNKLEK**LNSLLTYIKSEITQQEKGLNELSIR**
**DVTHTLGMLDDNKMDSVKEDVKNNQELDQ**EYRSCETQDESEIKDDETSCDNYHPM**NVDETSDEVSR**GIPRLSEDELR**ELD**
**VELIESKIN**ELSYYVEETNVDIGVLEEYARRLAEFKRR**KLDLNNAVQKRDEVKEQLGILKKKRFDEFMAGFNIISMTLKE**
**MYQMITMGGNAELELVDSLDPFSEGVTFSVMPPK**KSWR**NITNLSGGEKTLSSLALVFALHKYKPTPLYVMDEIDAALDFR**
**NVSIVANYIK**ERTK**NAQFIVISLRNNMFELAQQLVGVYK**R**DNRTKSTTIKNIDILNR**T

**Figure S5. Mass Spectrometric Identification of Mitotic Smc4 Phosphorylation Sites, Related to Figure 3**

Pk epitope-tagged Smc4 was immunoprecipitated from cells synchronized in mitosis by nocodazole treatment. The immunoprecipitate was resolved by SDS-PAGE and the portion of the gel corresponding to Coomassie-stained Smc4 was excised and processed for mass spectrometry. The experiment was repeated and gel slices were digested with trypsin and AspN proteases, respectively. Shown is the combined peptide coverage (bold) of Smc4, encompassing 986 of its 1418 amino acids (70%). Identified phosphorylation sites are highlighted in red. Cdk consensus recognition motifs (SP or TP) are underlined.

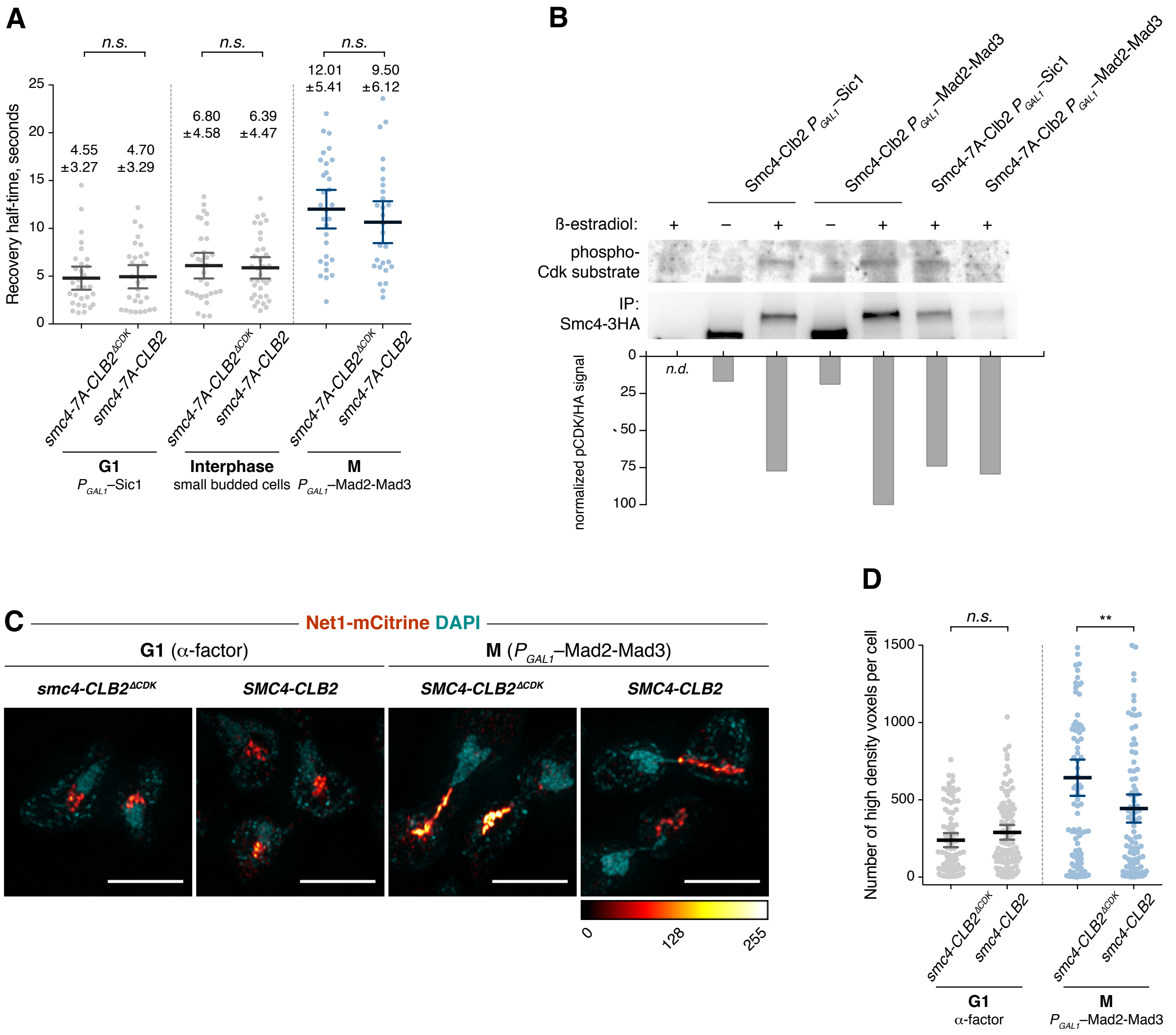


**Figure S6. Additional Condensin Dynamics and Chromosome Condensation Assays, Related to Figure 4**

(A) Fluorescence recovery half-times, following photobleaching, of the Brn1-3mNeonGreen signal in homozygous diploid *smc4*$^{7A}$*-CLB2*$^{\Delta CDK}$ and *smc4*$^{7A}$*-CLB2* cells in G1, interphase (asynchronous small budded cells) and metaphase. Error bars represent mean ± s.d. ($n \geq 30$, n.s. = not significant; ordinary one-way ANOVA, Sidak's multiple comparison test). The absence of statistically significant differences between the two strains indicates that Smc4$^{7A}$ is refractory to Cdk regulation.

(B) Phosphorylation of Smc4-Clb2 fusion, under the indicated conditions and strain backgrounds, assessed by immunoprecipitation by means of the HA tag, followed by Western blotting with an anti phospho–Cdk substrate antibody. ß-estradiol was used to produce the Cre-recombinase mediated Smc4-Clb2 fusion.

(C) Examples of rDNA morphology and Net1-mCitrine intensities during the experiment described in (D).

(D) Chromosome condensation of *smc4-CLB2*$^{\Delta CDK}$ and *smc4-CLB2* strains in a G1 arrest produced with α-factor or a metaphase arrest due to overexpression of Mad2-Mad3. Shown are the means with 95% confidence intervals ($n \geq 86$, ** $p < 0.01$; ordinary one-way ANOVA, Sidak's multiple comparison test). There is no significant difference between the two strains in G1, but Smc4-Clb2 causes impaired chromosome condensation in metaphase.

## SUPPLEMENTAL EXPERIMENTAL PROCEDURES

### Resource Table

| Reagent or Resource | Source | Identifier |
|---|---|---|
| **Antibodies** | | |
| anti-HA, mouse | in-house | clone 12CA5 |
| anti-HA, rat | Roche | clone 3F10 |
| anti-HA, mouse | Santa Cruz Biotech | clone F-7, cat. sc-7392 |
| anti-Pk, mouse | Bio-Rad/AbD Serotec | clone SV5-Pk1 |
| anti-myc, mouse | in-house | clone 9E10 |
| anti-tubulin, mouse | in-house | clone TAT-1 |
| anti-Smc2 polyclonal serum | A. Strunnikov | |
| anti-phosphoCDK | Cell Signaling | cat. #9477 |
| Atto 594 coupled GFP booster nanobody | Chromotek | cat. gba594 |
| AlexaFluor 594 goat anti-rat secondary | Molecular Probes | cat. A-11007 |
| **Chemicals and Peptides** | | |
| α-factor | in-house | |
| DAPI | Sigma-Aldrich | D9542 |
| Nocodazole | Sigma-Aldrich | M1404 |
| Trypsin Gold, Mass Spectrometry Grade | Promega | V5280 |
| Indole acetic acid (IAA) | Sigma-Aldrich | I3750 |
| **Critical Commercial Assays** | | |
| – | | |
| **Experimental Models: Organisms/Strains** | | |
| A list of the budding yeast strains used in this study, together with their genotypes, can be found in Table S1. | | |
| **Software and Algorithms** | | |
| ImageJ/Fiji | Open-source | http://fiji.sc |
| Zen (Black edition) | Zeiss | https://www.zeiss.com |
| Prism | Prism | https://www.graphpad.com |

### Yeast Strains and Techniques

Genes were tagged at their endogenous loci (*NET1, BRN1, SMC2 and SMC4*) by gene targeting using polymerase chain reaction (PCR) products (Knop et al., 1999; Sheff and Thorn, 2004). All vectors were constructed using sequence-independent cloning using InFusion HD reagents (Clontech/Takara Bio). *SMC2* and *SMC4*, along with their promoters and a 3HA epitope tag, were amplified from a wild type *W303* strain and cloned into the yeast integration vectors YIplac211 and YIplac204 respectively (Gietz and Sugino, 1988). ATPase mutants were constructed from the wild type vectors using the Q5 site-directed mutagenesis kit (New England Biolabs) following the manufacturer's instructions. Overexpression of Sic1, Mad2-Mad3 and Bfa1 was achieved by cloning the genes under the control of the *GAL1* promoter into YIplac128. Unless otherwise stated, yeast cultures were grown in rich YP medium supplemented with 2% glucose, with 2% raffinose, or with 2% raffinose + 2% galactose (Rose et al., 1990). Cell synchronization using α-factor block and release was performed as described (O'Reilly et al., 2012). For depletion of endogenous condensin SMC subunits, we used C-terminal mini auxin-induced degron tags with an additional epitope tag to aid detection (3Pk-miniAID; Kubota et al., 2013), and treated the cultures with 1 mM indole-3-acetic acid. For induction of Smc4-Clb2 fusions, we activated Cre recombinase fused to an estrogen binding domain EBD78 as previously described (Kuilman et al., 2014) by addition 1 μM β-estradiol to the growth medium overnight.

### Western Blotting and Immunoprecipitation

Protein extracts for western blotting were prepared following cell fixation using trichloroacetic acid, as previously described (Foiani et al., 1994), and analyzed by SDS-polyacrylamide gel electrophoresis. Antibodies used for detection are listed in the Resource Table. Chemiluminescent detection was performed using ECL Prime reagents

(GE Life Sciences); membranes were scanned as a time series using an ImageQuant LAS4000 imager in the linear detection range, and unsaturated images were selected for quantification.

For immunoprecipitation of Protein A-tagged condensin complexes, $3\times10^8$ cells were harvested, washed with distilled water and resuspended in 400 µl extraction buffer (50 mM HEPES/KOH pH 8.0, 0.1 M KCl, 2.5 mM $MgCl_2$, 10 % glycerol, 0.1 % SDS, 0.25 % Triton, 1 mM DTT, 1 mM PMSF, protease inhibitors). Cells were broken with glass beads, clarified by centrifugation and pre-cleared by incubation with protein A sepharose beads (GE Healthcare) at 4 °C for 1-2 hours. Protein A fusion proteins were immunoprecipitated from the pre-cleared extracts using IgG sepharose beads (GE Healthcare) at 4 °C for 1-2 hours. The beads were washed extensively with extraction buffer and the proteins eluted either with elution buffer (50 mM Tris-HCl pH 8.0, 10 mM EDTA, 1 % SDS) at 65 °C or by directly boiling the beads in SDS sample buffer. Elutions were analyzed for their protein content by SDS-polyacrylamide gel electrophoresis and immunoblotting.

For immunoprecipitation of HA-tagged Smc4-Clb2 fusion proteins, $3\times10^8$ cells were harvested, washed with distilled water and resuspended in 400 µl cold extraction buffer (50 mM HEPES/KOH pH 8.0, 0.1 M KCl, 2.5 mM $MgCl_2$, 10 % glycerol, 0.1 % SDS, 0.25 % Triton, 1 mM DTT, 1 mM PMSF, protease inhibitors). Cells were broken with glass beads, clarified by centrifugation and pre-cleared by incubation with Protein G magnetic beads (GE Healthcare) at 4 °C for 30-60 mins. Pre-cleared extracts were incubated with ~3 µg of anti-HA antibody (clone 12CA5) at 4 °C overnight. HA-tagged proteins were then immunoprecipitated with Protein G magnetic beads at 4 °C for 1 hour. The beads were washed extensively, and proteins were eluted by boiling in SDS sample buffer and analyzed by SDS-PAGE and immunoblotting. Due to low signal and high background, blots probed with phospho-CDK substrate antibodies were exposed to chemiluminescent film overnight, scanned and subjected to 100-pixel rolling ball background subtraction in ImageJ/Fiji prior to quantification.

**Flow Cytometry**

For preparation of samples for flow cytometry, cells from 1 ml of culture ($OD_{600} > 0.15$) were spun down, fixed in 1 ml ice-cold 70% ethanol, and incubated at 4 °C overnight. Cells were pelleted and resuspended in 1 ml of 50 mM Tris-HCl, pH 7.5 containing 0.1 mg/ml RNase A (1:100 from 10 mg/ml stock, DNase free after boiling). The cells were incubated at 37 °C for 2–16 hours, pelleted and resuspended in 0.4 ml of FACS buffer (200 mM Tris-HCl, pH 7.5; 211 mM NaCl; 78 mM $MgCl_2$) containing 50 µg/ml propidium iodide (1:20 from 1 mg/ml propidium iodide stock in water). Samples were sonicated for 5 seconds and stored in the dark at 4 °C until analysis. For analysis, 50–100 µl of stained cells were diluted in 600 µl of 50 mM Tris-HCl, pH 7.5. Immediately after dilution, 10000 events per sample were counted on a Beckton-Dickinson FACScan with settings in linear mode (FSC threshold: 52; FSC: detector: E01, amplifier: 1.4; SSC: detector: 400, amplifier: 1; FL1, FL3 off; FL2: detector: 750, amplifier: 7). The resulting cell counts were analyzed using FlowJo.

**Chromosome Spreads**

Cells from 2 ml of culture were harvested, resuspended in 1 ml S1 (100 mM potassium phosphate buffer, pH 7.4; 0.5 mM $MgCl_2$; 1.2 M sorbitol), and kept on ice until all samples from the course of the experiment were harvested. For spheroplastation, cells were resuspended in 200 µl S2 (S1 + per ml, 20 µl 1 M DTT and 14 µl 10 mg/ml zymolase T-100). The suspension was incubated in a 37 °C water bath for 20 min. 1 ml of ice-cold S3 (0.1 M 2-(N-morpholino)ethanesulfonic acid; 1 mM EDTA; 0.5 mM $MgCl_2$; 1 M sorbitol, pH 6.4) was added. Spheroplasts were washed and resuspended in 200 µl S3 and kept on ice until spreading. We used acid-washed 12 well multitest slides (MP Biomedicals, #6041205E) for spreading. The following were pipetted in rapid succession onto each well used: 1 µl cell suspension; 2 µl fixative (4% paraformaldehyde, dissolved in water at 60–80 °C; 3.4% sucrose; per ml, 1 µl 0.2 M NaOH); 2 µl spheroplast suspension; 4 µl 1% lipsol in water; 4 µl fixative. The drop was gently distributed onto the well using a pipette tip, avoiding contact with the glass. Slides were dried overnight at room temperature in a fume hood. For immunostaining, slides were placed in phosphate-buffered saline (PBS) for 10 min at room temperature. 50 µl of blocking buffer (1% BSA in PBS) was pipetted onto each well, and placed in a humid chamber for 1 hour at 25 °C. The blocking buffer was aspirated and 50 µl of primary antibody in blocking buffer was added to each well (e.g. rat anti-HA 3F10, 1:500), and incubated in a humid chamber for 2 hours at 25 °C. The wells were rinsed 3 times with PBS before adding 50 µl secondary antibody diluted in blocking buffer (e.g. AlexaFluor 594 anti-rat, 1:1000) to each well. The slide was incubated in a darkened humid chamber for 2 hours at 25 °C. Wells were again rinsed three times with PBS and then three times with water. DNA was then stained with 50 µl of a 100 µg/ml solution of DAPI in water pipetted onto each well, and incubated in the darkened humid chamber, 30 min, 25 °C. Wells were rinsed 3 times with water and aspirated. 5 µl of antifade (SlowFade Diamond, Life Technologies)

was pipetted onto each well. An acid washed coverslip (24 x 60 mm high precision, no. 1.5H, CellPath Ltd, SAN-2460-03A) was lowered onto the antifade mounting medium, and sealed with Valap (equal parts vaseline, lanolin & paraffin). The slide was stored in the dark at –20 °C until imaging.

For quantification of Smc staining intensity, images were typically acquired as detailed in 'Structured Illumination Microscopy' below. We created sum-intensity projections of the acquired image stacks, and used circles of 4.5 µm diameter drawn with the aid of DAPI-stained masses in the 405 nm channel, to measure the absolute staining intensity in the 592 nm channel. To exclude effects of variable ectopic protein expression, the absolute staining intensities were normalized to cellular expression levels of the HA-tagged Smc proteins as determined by loading-controlled Western blots in the same experiment. To minimize the effect of noise from dust and cellular debris, we excluded the extreme 5$^{th}$ percentile at either end from the analysis.

### ChIP-qPCR

Cells from the indicated strains growing in YPD were arrested in G1 by adding α-factor. After 2 hours, cells were released from the G1 block into YPD containing 88 µg/ml IAA and 5 µg/ml nocodazole. 2 hours after release, when the cultures were uniformly arrested in mitosis, cells were fixed with formaldehyde and harvested. Protein extracts were prepared and disrupted by sonication. The DNA fragments cross-linked to HA-tagged Smc4 were enriched by immuno-precipitation with anti HA-probe F7 antibody (Santa Cruz Biotechnology). After reversal of the cross-links, DNA both from immunoprecipitates and from total cell extracts was cleaned up and quantified using PowerUP SYBR® Green Master Mix (Applied Biosystems, Life Technologies) and a Quant Studio 12 Real-Time PCR System (Thermo Fisher Scientific). The following primers were used (CEN9 – centromere 9, tH(GUG)E1 – tRNA gene, RPL23B – ribosomal protein gene promoter, RPL34A – ribosomal protein gene promoter, N3 (CIN8) – negative binding site for normalization):

| | |
|---|---|
| CEN9 F | 5' TGTCACCTGGCTGTTTTGAG |
| CEN9 R | 5' TGGGTAATGTCAGCTGTGGA |
| tH(GUG)E1 F | 5' GAAACCCTGGTTCGATTCTAGGAG |
| tH(GUG)E1 R | 5' GCTCTCATGATCACCACATCTGAC |
| RPL23B F | 5' CCGTCAAGCTAAGTCTTGGAGAAG |
| RPL23B R | 5' CCTTAGGATTAGCGATGACACCAG |
| RPL34A F | 5' GTCTTGTGGGTCTTGGAAACAG |
| RPL34A R | 5' CAAAGTGTGGTGACTGTGGTAG |
| N3 (CIN8) F | 5' AGGGCACAACTAGATAAACAGCA |
| N3 (CIN8) R | 5' GGGCCATTTGCATTACCTCAGTCA |

### Cell Fixation and Nanobody Staining

Cells were fixed and stained using an adapted published method (Ries et al., 2012). Briefly, we fixed cells by addition of 3.6% formaldehyde directly to the liquid culture (1:10 by volume from 36% stock solution of EM grade formaldehyde, TAAB, #F003). After incubation on a roller for 15 min at room temperature, the cells were washed once with TBS (150 mM NaCl, 50 mM Tris-Cl pH 8.0) containing 50 mM $NH_4Cl$ and then 3x with TBS. Cells were permeabilized in blocking buffer (TBS + 5% BSA, 0.25% TritonX-100, 0.005% $NaN_3$) for 1 hour at room temperature. Nanobody staining was then performed at 4 °C overnight in staining buffer (TBS + 1% BSA, 0.05% TritonX-100, 0.001% $NaN_3$) with 1/100 Atto 594–conjugated GFP booster nanobody (Chromotek, gba594). Cells were then washed 3x in TBS, stained with 0.1 µg/ml DAPI in TBS, washed 3x, mounted on acid-washed high precision (170 µm ± 5 µm thick) coverslips, and sealed with Valap.

### Structured Illumination Microscopy

Structured illumination microscopy was performed on an API OMX v3 microscope with a 100x objective and a 'live' filter set: DAPI – excitation 405 nm, emission 465-500 nm; YFP – excitation 514 nm, emission 525-575 nm; mCherry/Alexa 594 – excitation 592.5 nm, emission 602-656 nm. We typically acquired images of 512 x 512 pixels at 1 x 1 binning and, per channel, 33 slices at 0.125 µm spacing for a total sample thickness of 4 µm. For images not acquired in structured illumination mode (YFP – 60 ms exposure, 1%T), deconvolution was applied in softWoRx using the conservative ratio method with medium noise filtering and an experimentally determined point spread function. For images acquired in structured illumination mode (DAPI – 60 ms exposure, 50%T and Alexa 594 – 100

ms exposure, 33.3%T), default reconstruction parameters were used. For multichannel images, registration parameters for aligning the different channels were determined empirically using a 10 nm–spacing grid slide and Tetraspeck beads.

**Chromosome Condensation Assay**

In order to extract the high frequency sub-diffraction information provided by SIM over conventional imaging, we adapted a published method (Marbouty et al., 2015) that applied a 3D Butterworth bandpass filter with spatial cutoffs at 100 nm and 400 nm. Using ImageJ, we applied an analogous Fourier bandpass frequency filter with cutoffs at 3 and 10 pixels (5% tolerance with no stripe suppression), which corresponded to structures between 118.5 and 395 pixels (39.5 nm/pixel). We then automatically selected the brightest pixels in each cell volume by using ImageJ MaxEntropy thresholding with a stack histogram for each cell (Kapur et al., 1985). Finally, we counted the number of outlined pixels per slice (voxels); their sum served as a high resolution measure of chromosome condensation. Since SIM reconstructions of interphase cells were noisier due to lower signal intensity, we excluded the extreme $5^{th}$ percentile of values in experiments that included G1 cells.

**Table S1. Yeast Strains Used in this Study**

| Strain | Genotype | Background | Source | Fig |
|---|---|---|---|---|
| Y4267 | *MAT*a *ade2-1::OsTIR1-9myc:ADE2* | W303 | Lab collection | S1A, S1B, S6B |
| Y4279/ RT157 | *MAT*a *ade2-1::OsTIR1-9myc:ADE2 SMC2-3HA:URA3* | W303 | This study | 1A-B, S1A, S2A-C |
| Y4276/ RT146 | *MAT*a *ade2-1::OsTIR1-9myc:ADE2 SMC2-3Pk-miniAID:kanR* | W303 | This study | 1A-B, S1A, S2A-C |
| Y4973/ RT427 | *MAT*a *ade2-1::OsTIR1-9myc:ADE2 SMC2-3Pk-miniAID:kanR ura3-1::*$P_{SMC2}$*–SMC2*$^{WT}$*-3HA:URA3* | W303 | This study | 1A-B, S1A, S2A-C |
| Y4974/ RT429 | *MAT*a *ade2-1::OsTIR1-9myc:ADE2 SMC2-3Pk-miniAID:kanR ura3-1::*$P_{SMC2}$*–SMC2*$^{K38A}$*-3HA:URA3* | W303 | This study | 1A-B, S1A, S2A-C |
| Y4975/ RT431 | *MAT*a *ade2-1::OsTIR1-9myc:ADE2 SMC2-3Pk-miniAID:kanR ura3-1::*$P_{SMC2}$*–SMC2*$^{R58A}$*-3HA:URA3* | W303 | This study | 1A-B, S1A, S2A-C |
| Y4976/ RT433 | *MAT*a *ade2-1::OsTIR1-9myc:ADE2 SMC2-3Pk-miniAID:kanR ura3-1::*$P_{SMC2}$*–SMC2*$^{R58K}$*-3HA:URA3* | W303 | This study | 1A-B, S1A, S2A-C |
| Y4977/ RT435 | *MAT*a *ade2-1::OsTIR1-9myc:ADE2 SMC2-3Pk-miniAID:kanR ura3-1::*$P_{SMC2}$*–SMC2*$^{S1085R}$*-3HA:URA3* | W303 | This study | 1A-B, S1A, S2A-C |
| Y4978 RT437 | *MAT*a *ade2-1::OsTIR1-9myc:ADE2 SMC2-3Pk-miniAID:kanR ura3-1::*$P_{SMC2}$*–SMC2*$^{E1113Q}$*-3HA:URA3* | W303 | This study | 1A-B, S1A, S2A-C |
| Y4979/ RT439 | *MAT*a *ade2-1::OsTIR1-9myc:ADE2 SMC2-3Pk-miniAID:kanR ura3-1::*$P_{SMC2}$*–SMC2*$^{E1113D}$*-3HA:URA3* | W303 | This study | 1A-B, S1A, S2A-C |
| Y4280/ RT159 | *MAT*a *ade2-1::OsTIR1-9myc:ADE2 SMC4-3HA:TRP1* | W303 | This study | 1C-D, S1B, S2D-F |
| Y4278/ RT149 | *MAT*a *ade2-1::OsTIR1-9myc:ADE2 SMC4-3Pk-miniAID:kanR* | W303 | This study | 1C-E, S1B, S2D-F |
| Y4966/ RT242 | *MAT*a *ade2-1::OsTIR1-9myc:ADE2 SMC4-3Pk-miniAID:kanR trp1-1::*$P_{SMC4}$*–SMC4*$^{WT}$*-3HA:TRP1* | W303 | This study | 1C-E, S1B, S2D-F |
| Y4967/ RT244 | *MAT*a *ade2-1::OsTIR1-9myc:ADE2 SMC4-3Pk-miniAID:kanR trp1-1::*$P_{SMC4}$*–SMC4*$^{K191A}$*-3HA:TRP1* | W303 | This study | 1C-E, S1B, S2D-F |
| Y4968/ RT246 | *MAT*a *ade2-1::OsTIR1-9myc:ADE2 SMC4-3Pk-miniAID:kanR trp1-1::*$P_{SMC4}$*–SMC4*$^{R210A}$*-3HA:TRP1* | W303 | This study | 1C-E, S1B, S2D-F |
| Y4969/ RT248 | *MAT*a *ade2-1::OsTIR1-9myc:ADE2 SMC4-3Pk-miniAID:kanR trp1-1::*$P_{SMC4}$*–SMC4*$^{R210K}$*-3HA:TRP1* | W303 | This study | 1C-E, S1B, S2D-F |
| Y4970/ RT250 | *MAT*a *ade2-1::OsTIR1-9myc:ADE2 SMC4-3Pk-miniAID:kanR trp1-1::*$P_{SMC4}$*–SMC4*$^{S1324R}$*-3HA:TRP1* | W303 | This study | 1C-E, S1B, S2D-F |
| Y4971/ RT252 | *MAT*a *ade2-1::OsTIR1-9myc:ADE2 SMC4-3Pk-miniAID:kanR trp1-1::*$P_{SMC4}$*–SMC4*$^{E1352Q}$*-3HA:TRP1* | W303 | This study | 1C-E, S1B, S2D-F |
| Y4972/ RT254 | *MAT*a *ade2-1::OsTIR1-9myc:ADE2 SMC4-3Pk-miniAID:kanR trp1-1::*$P_{SMC4}$*–SMC4*$^{E1352D}$*-3HA:TRP1* | W303 | This study | 1C-E, S1B, S2D-F |
| Y3654 | *MAT*a *BRN1-18myc::TRP1 SMC2-2ProtA:SpHIS5* | S288C | This study | 1F |
| Y3733 | *MAT*a *BRN1-18myc:TRP1* | S288C | This study | 1F-G |
| Y3600 | *MAT*a *smc2-8 BRN1-18myc::LEU2 pRS306-SMC2-6His-HA-2ProtA:URA3* | S288C | This study | 1F |
| Y3736 | *MAT*a *smc2-8 BRN1-18myc:LEU2 pRS306-smc2K38A-6His-HA-2ProtA:URA3* | S288C | This study | 1F |
| Y3735 | *MAT*a *smc2-8 BRN1-18myc:LEU2 pRS306-smc2R58A-6His-HA-2ProtA:URA3* | S288C | This study | 1F |
| Y3657 | *MAT*a *smc2-8 BRN1-18myc:LEU2 pRS306-smc2S1085R-6His-HA-2ProtA:URA3* | S288C | This study | 1F |
| Y3656 | *MAT*a *smc2-8 BRN1-18myc:LEU2 pRS306-smc2E1131Q-6His-HA-2ProtA:URA3* | S288C | This study | 1F |
| Y3652 | *MAT*a *BRN1-18myc::KlTrp1 SMC4-2protA::SpHIS5* | S288C | This study | 1G |
| Y3601 | *MAT*a *smc4-1 BRN1-18myc::SpHIS5 pRS306-SMC4-6His-HA-2ProtA:URA3* | S288C | This study | 1G |
| Y3738 | *MAT*a *smc4-1 BRN1-18myc::SpHIS5 pRS306-smcK191A-6His-HA-2ProtA:URA3* | S288C | This study | 1G |
| Y3739 | *MAT*a *smc4-1 BRN1-18myc::SpHIS5 pRS306-smc4R210A-6His-HA-2ProtA:URA3* | S288C | This study | 1G |

| | | | | |
|---|---|---|---|---|
| Y3644 | *MAT*a *smc4-1 BRN1-18myc::SpHIS5 pRS306-smc4S1324R-6His-HA-2ProtA:URA3* | S288C | This study | 1G |
| Y3658 | *MAT*a *smc4-1 BRN1-18myc::SpHIS5 pRS306-smc4E1352Q-6His-HA-2ProtA:URA3* | S288C | This study | 1G |
| Y4998/ RT346a | *MAT*a *ade2-1::OsTIR1-9myc:ADE2 SMC4-3HA:TRP1 NET1-yEmCitrine:HIS3 leu2-2,112::*$P_{GAL1}$*–MAD2-MAD3:LEU2* | W303 | This study | 2A-B, S4A-B, 3D-E |
| Y4997/ RT315 | *MAT*a *ade2-1::OsTIR1-9myc:ADE2 SMC4-3Pk-miniAID:kanR NET1-yEmCitrine:HIS3 leu2-2,112::*$P_{GAL1}$*–MAD2-MAD3:LEU2* | W303 | This study | 2A-B |
| Y4980/ RT401 | *MAT*a *ade2-1::OsTIR1-9myc:ADE2 SMC4-3Pk-miniAID:kanR trp1-1::*$P_{SMC4}$*–*$SMC4^{WT}$*-3HA:TRP1 NET1-yEmCitrine:HIS3 leu2-2,112::*$P_{GAL1}$*–MAD2-MAD3:LEU2* | W303 | This study | 2A-B |
| Y4981/ RT402 | *MAT*a *ade2-1::OsTIR1-9myc:ADE2 SMC4-3Pk-miniAID:kanR trp1-1::*$P_{SMC4}$*–*$SMC4^{K191A}$*-3HA:TRP1 NET1-yEmCitrine:HIS3 leu2-2,112::*$P_{GAL1}$*–MAD2-MAD3:LEU2* | W303 | This study | 2A-B |
| Y4982/ RT403 | *MAT*a *ade2-1::OsTIR1-9myc:ADE2 SMC4-3Pk-miniAID:kanR trp1-1::*$P_{SMC4}$*–*$SMC4^{R210A}$*-3HA:TRP1 NET1-yEmCitrine:HIS3 leu2-2,112::*$P_{GAL1}$*–MAD2-MAD3:LEU2* | W303 | This study | 2A-B |
| Y4983/ RT404 | *MAT*a *ade2-1::OsTIR1-9myc:ADE2 SMC4-3Pk-miniAID:kanR trp1-1::*$P_{SMC4}$*–*$SMC4^{R210K}$*-3HA:TRP1 NET1-yEmCitrine:HIS3 leu2-2,112::*$P_{GAL1}$*–MAD2-MAD3:LEU2* | W303 | This study | 2A-B |
| Y4984/ RT405 | *MAT*a *ade2-1::OsTIR1-9myc:ADE2 SMC4-3Pk-miniAID:kanR trp1-1::*$P_{SMC4}$*–*$SMC4^{S1324R}$*-3HA:TRP1 NET1-yEmCitrine:HIS3 leu2-2,112::*$P_{GAL1}$*–MAD2-MAD3:LEU2* | W303 | This study | 2A-B |
| Y4985/ RT406a | *MAT*a *ade2-1::OsTIR1-9myc:ADE2 SMC4-3Pk-miniAID:kanR trp1-1::*$P_{SMC4}$*–*$SMC4^{E1352Q}$*-3HA:TRP1 NET1-yEmCitrine:HIS3 leu2-2,112::*$P_{GAL1}$*–MAD2-MAD3:LEU2* | W303 | This study | 2A-B |
| Y4986/ RT407a | *MAT*a *ade2-1::OsTIR1-9myc:ADE2 SMC4-3Pk-miniAID:kanR trp1-1::*$P_{SMC4}$*–*$SMC4^{E1352D}$*-3HA:TRP1 NET1-yEmCitrine:HIS3 leu2-2,112::*$P_{GAL1}$*–MAD2-MAD3:LEU2* | W303 | This study | 2A-B |
| Y5001/ RT347 | *MAT*a *ade2-1::OsTIR1-9myc:ADE2 SMC4-3HA:TRP1 NET1-yEmCitrine:HIS3 leu2-2,112::*$P_{GAL1}$*–BFA1:LEU2* | W303 | This study | 2C-D, S4C |
| Y5000/ RT276 | *MAT*a *ade2-1::OsTIR1-9myc:ADE2 SMC4-3Pk-miniAID:kanR NET1-yEmCitrine:HIS3 leu2-2,112::*$P_{GAL1}$*–BFA1:LEU2* | W303 | This study | 2C-D |
| Y4987/ RT408 | *MAT*a *ade2-1::OsTIR1-9myc:ADE2 SMC4-3Pk-miniAID:kanR trp1-1::*$P_{SMC4}$*–*$SMC4^{WT}$*-3HA:TRP1 NET1-yEmCitrine:HIS3 leu2-2,112::*$P_{GAL1}$*–BFA1:LEU2* | W303 | This study | 2C-D |
| Y4988/ RT409 | *MAT*a *ade2-1::OsTIR1-9myc:ADE2 SMC4-3Pk-miniAID:kanR trp1-1::*$P_{SMC4}$*–*$SMC4^{K191A}$*-3HA:TRP1 NET1-yEmCitrine:HIS3 leu2-2,112::*$P_{GAL1}$*–BFA1:LEU2* | W303 | This study | 2C-D |
| Y4989/ RT410 | *MAT*a *ade2-1::OsTIR1-9myc:ADE2 SMC4-3Pk-miniAID:kanR trp1-1::*$P_{SMC4}$*–*$SMC4^{R210A}$*-3HA:TRP1 NET1-yEmCitrine:HIS3 leu2-2,112::*$P_{GAL1}$*–BFA1:LEU2* | W303 | This study | 2C-D |
| Y4990/ RT411 | *MAT*a *ade2-1::OsTIR1-9myc:ADE2 SMC4-3Pk-miniAID:kanR trp1-1::*$P_{SMC4}$*–*$SMC4^{R210K}$*-3HA:TRP1 NET1-yEmCitrine:HIS3 leu2-2,112::*$P_{GAL1}$*–BFA1:LEU2* | W303 | This study | 2C-D |
| Y4991/ RT412 | *MAT*a *ade2-1::OsTIR1-9myc:ADE2 SMC4-3Pk-miniAID:kanR trp1-1::*$P_{SMC4}$*–*$SMC4^{S1324R}$*-3HA:TRP1 NET1-yEmCitrine:HIS3 leu2-2,112::*$P_{GAL1}$*–BFA1:LEU2* | W303 | This study | 2C-D |
| Y4992/ RT413a | *MAT*a *ade2-1::OsTIR1-9myc:ADE2 SMC4-3Pk-miniAID:kanR trp1-1::*$P_{SMC4}$*–*$SMC4^{E1352Q}$*-3HA:TRP1 NET1-yEmCitrine:HIS3 leu2-2,112::*$P_{GAL1}$*–BFA1:LEU2* | W303 | This study | 2C-D |
| Y4993/ RT414a | *MAT*a *ade2-1::OsTIR1-9myc:ADE2 SMC4-3Pk-miniAID:kanR trp1-1::*$P_{SMC4}$*–*$SMC4^{E1352D}$*-3HA:TRP1 NET1-yEmCitrine:HIS3 leu2-2,112::*$P_{GAL1}$*–BFA1:LEU2* | W303 | This study | 2C-D |
| Y5004/ RT379a | *MAT*a/*MAT*α *ade2-1/ade2-1::OsTIR1-9myc:ADE2 BRN1-3mNeonGreen:HIS3/BRN1-3mNeonGreen:NAT trp1-1/trp1-1::*$P_{GAL1}$*–*$SIC1^{V5.V33.A76}$*-HA:TRP1* | W303 | This study | 2E, 3F |
| Y5005/ RT380a | *MAT*a/*MAT*α *ade2-1/ade2-1::OsTIR1-9myc:ADE2 BRN1-3mNeonGreen:HIS3/BRN1-3mNeonGreen:NAT leu2-2,112/leu2-2,112::*$P_{GAL1}$*–MAD2-MAD3:LEU2* | W303 | This study | 2E-F, 3F |

| | | | | |
|---|---|---|---|---|
| Y5007/ RT394 | *MAT*a/*MAT*α *ade2-1/ade2-1::OsTIR1-9myc:ADE2 BRN1-3mNeonGreen:HIS3/BRN1-3mNeonGreen:NAT SMC4*$^{R210A}$*::TRP/SMC4*$^{R210A}$*::TRP ura3-1/ura3-1::*$P_{GAL1}$*–SIC1*$^{V5.V33.A76}$*-HA:URA3* | W303 | This study | 2E |
| Y5008/ RT395 | *MAT*a/*MAT*α *ade2-1/ade2-1::OsTIR1-9myc:ADE2 BRN1-3mNeonGreen:HIS3/BRN1-3mNeonGreen:NAT SMC4*$^{R210A}$*::TRP/SMC4*$^{R210A}$*::TRP leu2-2,112/leu2-2,112::*$P_{GAL1}$*–MAD2-MAD3:LEU2* | W303 | This study | 2E-F |
| Y4426 | *SMC4-9Pk:URA3* | W303 | This study | 3B-C |
| Y4999/ RT351 | *MAT*a *ade2-1::OsTIR1-9myc:ADE2 SMC4*$^{7A}$*-3HA:URA3:TRP1 NET1-yEmCitrine:HIS3 leu2-2,112::*$P_{GAL1}$*–MAD2-MAD3:LEU2* | W303 | This study | 3D-E |
| Y5010/ RT399 | *MAT*a/*MAT*α *ade2-1/ade2-1::OsTIR1-9myc:ADE2 BRN1-3mNeonGreen:HIS3/BRN1-3mNeonGreen:NAT SMC4*$^{7A}$*:URA3/SMC4*$^{7A}$*:URA3 trp1-1/trp1-1::*$P_{GAL1}$*–SIC1*$^{V5.V33.A76}$*-HA:TRP1* | W303 | This study | 3F |
| Y5011/ RT400 | *MAT*a/*MAT*α *ade2-1/ade2-1::OsTIR1-9myc:ADE2 BRN1-3mNeonGreen:HIS3/BRN1-3mNeonGreen:NAT SMC4*$^{7A}$*:URA3/SMC4*$^{7A}$*:URA3 leu2-2,112/leu2-2,112::*$P_{GAL1}$*–MAD2-MAD3:LEU2* | W303 | This study | 3F |
| Y5016/ RT441 | *MAT*a/*MAT*α *ade2-1/ade2-1::OsTIR1-9myc:ADE2 BRN1-3mNeonGreen::HIS3/BRN1-3mNeonGreen::NAT SMC4-CLB2-3HA:kanR/SMC4-CLB2-3HA:TRP1 leu2-2,112/leu2-2,112::*$P_{GAL1}$*–SIC1*$^{V5.V33.A76}$*–Cre-EBD78* | W303 | This study | 4B, S6B |
| Y5017/ RT443 | *MAT*a/*MAT*α *ade2-1/ade2-1::OsTIR1-9myc:ADE2 BRN1-3mNeonGreen::HIS3/BRN1-3mNeonGreen::NAT SMC4-CLB2-3HA:kanR/SMC4-CLB2-3HA:TRP1 leu2-2,112/leu2-2,112::*$P_{GAL1}$*–MAD2-MAD3–Cre-EBD78* | W303 | This study | 4B, S6B |
| Y5018/ RT445 | *MAT*a/*MAT*α *ade2-1/ade2-1::OsTIR1-9myc:ADE2 BRN1-3mNeonGreen::HIS3/BRN1-3mNeonGreen::NAT SMC4-CLB2*$^{\Delta CDK}$*-3HA:kanR/SMC4-CLB2*$^{\Delta CDK}$*-3HA:TRP1 leu2-2,112/leu2-2,112::*$P_{GAL1}$*–SIC1*$^{V5.V33.A76}$*–Cre-EBD78* | W303 | This study | 4B |
| Y5019/ RT447 | *MAT*a/*MAT*α *ade2-1/ade2-1::OsTIR1-9myc:ADE2 BRN1-3mNeonGreen::HIS3/BRN1-3mNeonGreen::NAT SMC4-CLB2*$^{\Delta CDK}$*-3HA:kanR/SMC4-CLB2*$^{\Delta CDK}$*-3HA:TRP1 leu2-2,112/leu2-2,112::*$P_{GAL1}$*–MAD2-MAD3–Cre-EBD78* | W303 | This study | 4B |
| Y5020/ RT449 | *MAT*a/*MAT*α *ade2-1/ade2-1::OsTIR1-9myc:ADE2 BRN1-3mNeonGreen::HIS3/BRN1-3mNeonGreen::NAT SMC4*$^{7A}$*-CLB2-3HA:kanR:URA3/SMC4*$^{7A}$*-CLB2-3HA:TRP1:URA3 leu2-2,112/leu2-2,112::*$P_{GAL1}$*–SIC1*$^{V5.V33.A76}$*–Cre-EBD78* | W303 | This study | S6A, S6B |
| Y5021/ RT451 | *MAT*a/*MAT*α *ade2-1/ade2-1::OsTIR1-9myc:ADE2 BRN1-3mNeonGreen::HIS3/BRN1-3mNeonGreen::NAT SMC4*$^{7A}$*-CLB2-3HA:kanR:URA3/SMC4*$^{7A}$*-CLB2-3HA:TRP1:URA3 leu2-2,112/leu2-2,112::*$P_{GAL1}$*–MAD2-MAD3–Cre-EBD78* | W303 | This study | S6A, S6B |
| Y5022/ RT453 | *MAT*a/*MAT*α *ade2-1/ade2-1::OsTIR1-9myc:ADE2 BRN1-3mNeonGreen::HIS3/BRN1-3mNeonGreen::NAT SMC4*$^{7A}$*-CLB2*$^{\Delta CDK}$*-3HA:kanR:URA3/SMC4*$^{7A}$*-CLB2*$^{\Delta CDK}$*-3HA:TRP1:URA3 leu2-2,112/leu2-2,112::*$P_{GAL1}$*–SIC1*$^{V5.V33.A76}$*–Cre-EBD78* | W303 | This study | S6A |
| Y5023/ RT455 | *MAT*a/*MAT*α *ade2-1/ade2-1::OsTIR1-9myc:ADE2 BRN1-3mNeonGreen::HIS3/BRN1-3mNeonGreen::NAT SMC4*$^{7A}$*-CLB2*$^{\Delta CDK}$*-3HA:kanR:URA3/SMC4*$^{7A}$*-CLB2*$^{\Delta CDK}$*-3HA:TRP1:URA3 leu2-2,112/leu2-2,112::*$P_{GAL1}$*–MAD2-MAD3–Cre-EBD78* | W303 | This study | S6A |
| Y5027/ RT463 | *MAT*a *ade2-1::OsTIR1-9myc:ADE2 NET1-yEmCitrine::HIS3 SMC4-CLB2:TRP1 leu2-3,112::*$P_{GAL1}$*–MAD2-MAD3–Cre-EBD78* | W303 | This study | S6C-D |
| Y5029/ RT467 | *MAT*a *ade2-1::OsTIR1-9myc:ADE2 NET1-yEmCitrine::HIS3 SMC4-CLB2*$^{\Delta CDK}$*:TRP1 leu2-3,112::*$P_{GAL1}$*–MAD2-MAD3–Cre-EBD78* | W303 | This study | S6C-D |

**SUPPLEMENTAL REFERENCES**

Foiani, M., Marini, F., Gamba, D., Lucchini, G., and Plevani, P. (1994). The B subunit of the DNA polymerase a-primase complex in *Saccharomyces cerevisiae* executes an essential function at the initial stage of DNA replication. Mol Cell Biol *14*, 923-933.

Gietz, R.D., and Sugino, A. (1988). New yeast-*Escherichia coli* shuttle vectors constructed with in vitro mutagenized yeast genes lacking six-base restriction sites. Gene *74*, 527-534.

Kapur, J.N., Sahoo, P.K., and Wong, A.C.K. (1985). A new method for gray-level picture thresholding using the entropy of the histogram. Graph Models Image Process *29*, 273-285.

Knop, M., Siegers, K., Pereira, G., Zachariae, W., Winsor, B., Nasmyth, K., and Schiebel, E. (1999). Epitope tagging of yeast genes using a PCR-based strategy: more tags and improved practical routines. Yeast *15*, 963-972.

Kubota, T., Nishimura, K., Kanemaki, M.T., and Donaldson, A.D. (2013). The Elg1 replication factor C-like complex functions in PCNA unloading during DNA replication. Mol Cell *50*, 273-280.

Kuilman, T., Maiolica, A., Godfrey, M., Scheidel, N., Aebersold, R., and Uhlmann, F. (2014). Identification of Cdk targets that control cytokinesis. EMBO J *34*, 81-96.

Marbouty, M., Le Gall, A., Cattoni, D.I., Cournac, A., Koh, A., Fiche, J.B., Mozziconacci, J., Murray, H., Koszul, R., and Nollmann, M. (2015). Condensin- and replication-mediated bacterial chromosome folding and origin condensation revealed by Hi-C and super-resolution imaging. Mol Cell *59*, 588-602.

O'Reilly, N., Charbin, A., Lopez-Serra, L., and Uhlmann, F. (2012). Facile synthesis of budding yeast **a**-factor and its use to synchronize cells of a mating type. Yeast *29*, 233-240.

Ries, J., Kaplan, C., Platonova, E., Eghlidi, H., and Ewers, H. (2012). A simple, versatile method for GFP-based super-resolution microscopy via nanobodies. Nat Methods *9*, 582-584.

Rose, M.D., Winston, F., and Hieter, P. (1990). Laboratory course manual for methods in yeast genetics (Cold Spring Harbor, NY: Cold Spring Harbor Laboratory Press).

Sheff, M.A., and Thorn, K.S. (2004). Optimized cassettes for fluorescent protein tagging in *Saccharomyces cerevisiae*. Yeast *21*, 661-670.
