# Supplementary material for: Cell-Cycle Regulation of Dynamic Chromosome Association of the Condensin Complex
Source: Cell Rep. 2018 May 22;23(8):2308–17. doi: 10.1016/j.celrep.2018.04.082 (PMC5986713; doi:10.1016/j.celrep.2018.04.082)
Supplement: Document S2. Article plus Supplemental Information [file mmc2.pdf]

Cell Reports

Report

Cell-Cycle Regulation of Dynamic Chromosome Association of the Condensin Complex

Graphical Abstract

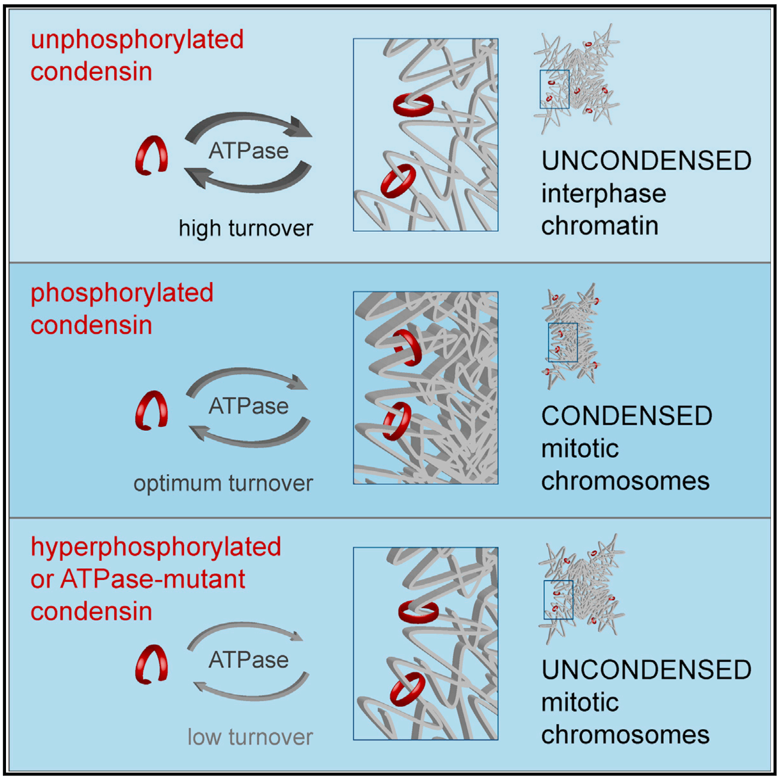

Authors

Rahul Thadani, Julia Kamenz,
Sebastian Heeger, Sofía Muñoz,
Frank Uhlmann

Correspondence

frank.uhlmann@crick.ac.uk

In Brief

The condensin complex is a key determinant of mitotic chromosome formation. Thadani et al. study the dynamic binding of condensin to chromosomes. They reveal how condensin turnover is regulated by its ATPase and by cell-cycle phosphorylation. Chromosome condensation in mitosis requires fine-tuning of this dynamic behavior.

Highlights

- The budding yeast condensin ATPase promotes chromosome condensation
- The ATPase controls the dynamic chromosome binding cycle of the complex
- Smc4 phosphorylation by cyclin-dependent kinase adjusts the turnover
- Chromosome condensation requires fine-tuning of condensin behavior

Thadani et al., 2018, Cell Reports *23*, 2308–2317
May 22, 2018 © 2018 The Francis Crick Institute.
https://doi.org/10.1016/j.celrep.2018.04.082

CellPress

OPEN ACCESS
CellPress

Cell Reports
Report

Cell-Cycle Regulation of Dynamic Chromosome Association of the Condensin Complex

Rahul Thadani,[1,2] Julia Kamenz,[1,3] Sebastian Heeger,[1] Sofía Muñoz,[1] and Frank Uhlmann[1,4,*]
[1]Chromosome Segregation Laboratory, The Francis Crick Institute, London NW1 1AT, UK
[2]Laboratory of Biochemistry and Molecular Biology, National Cancer Institute, NIH, Bethesda, MD 20892, USA
[3]Present address: Department of Chemical and Systems Biology, Stanford University School of Medicine, Stanford, CA 94305-5174, USA
[4]Lead Contact
*Correspondence: frank.uhlmann@crick.ac.uk
https://doi.org/10.1016/j.celrep.2018.04.082

SUMMARY

Eukaryotic cells inherit their genomes in the form of chromosomes, which are formed from the compaction of interphase chromatin by the condensin complex. Condensin is a member of the structural maintenance of chromosomes (SMC) family of ATPases, large ring-shaped protein assemblies that entrap DNA to establish chromosomal interactions. Here, we use the budding yeast *Saccharomyces cerevisiae* to dissect the role of the condensin ATPase and its relationship with cell-cycle-regulated chromosome binding dynamics. ATP hydrolysis-deficient condensin binds to chromosomes but is defective in chromosome condensation and segregation. By modulating the ATPase, we demonstrate that it controls condensin's dynamic turnover on chromosomes. Mitosis-specific phosphorylation of condensin's Smc4 subunit reduces the turnover rate. However, reducing turnover by itself is insufficient to compact chromosomes. We propose that condensation requires fine-tuned dynamic condensin interactions with more than one DNA. These results enhance our molecular understanding of condensin function during chromosome condensation.

INTRODUCTION

The condensin complex is a key structural component of mitotic chromosomes (Hirano, 2016; Uhlmann, 2016). It consists of two structural maintenance of chromosomes (SMC) subunits, Smc2 and Smc4, that constitute the circumference of the condensin ring. They dimerize via a hinge domain on one side of the ring and, on the other side, at a pair of ATPase head domains that form the catalytic core of the complex. A kleisin subunit, Brn1 in budding yeast, bridges both ATPase heads. The kleisin subunit also recruits two additional HEAT repeat subunits to the condensin complex (Ycg1 and Ycs4 in budding yeast) (Onn et al., 2007). The ATPase motifs of vertebrate condensin are essential for chromosome condensation, and continual ATP hydrolysis is required to maintain the condensed state (Hudson et al., 2008; Kinoshita et al., 2015). *In vitro*, ATP hydrolysis by condensin promotes DNA supercoiling, but how this relates to chromosome condensation is not yet understood (Kimura and Hirano, 1997). Human condensin binds to chromosomes dynamically. Of the two human condensin isoforms, condensin II appears to turn over rapidly on chromosomes in interphase and bind stably during mitosis. Condensin I in turn accesses chromosomes after nuclear envelope breakdown and maintains dynamic chromosome association throughout mitosis (Gerlich et al., 2006). Simulations of chromatin chain behavior suggest that stabilization of stochastic condensin-mediated DNA-DNA interactions provides a potent driving force for chromosome compaction. An alternatively proposed condensation mechanism, that of loop extrusion or loop expansion, could also be aided by an increased mitotic condensin residence time (Alipour and Marko, 2012; Cheng et al., 2015; Fudenberg et al., 2016; Thadani et al., 2012). How dynamic chromosome binding is linked to condensin's ATPase activity and how cell-cycle-dependent condensin modifications regulate this dynamic binding cycle are incompletely understood.

RESULTS

The Condensin ATPase Is Essential for Yeast Cell Proliferation

To study the condensin ATPase, we generated a series of mutations in conserved residues of budding yeast Smc2 and Smc4 that were designed to disrupt aspects of the ATPase cycle, based on previous studies of SMC ATPases (Arumugam et al., 2003; Hopfner et al., 2000; Lammens et al., 2004; Lengronne et al., 2006; Weitzer et al., 2003). These include mutations in the Walker A motif (Smc2 K38A and Smc4 K191A) to disrupt ATP binding, the signature motif to prevent SMC head engagement (Smc2 S1085R and Smc4 S1324R), the Walker B motif to slow or prevent ATP hydrolysis (Smc2 E1113Q or E1113D and Smc4 E1352Q or E1352D), and an arginine finger to reduce DNA stimulation of ATP hydrolysis (Smc2 R58A or R58K and Smc4 R210A or R210K). The mutant proteins were expressed with C-terminal 3HA epitope tags from an ectopic locus under control of their authentic promoters. In the same strains, the endogenously encoded Smc2 or Smc4 proteins could be conditionally depleted by taking advantage of auxin-inducible degrons (*aid*) (Kubota et al., 2013).

We first tested the ability of ATPase mutant Smc2 and Smc4 to support cell growth. Smc2 or Smc4 depletion leads to loss of

 © 2018 The Francis Crick Institute.
This is an open access article under the CC BY license (http://creativecommons.org/licenses/by/4.0/).

OPEN ACCESS
CellPress

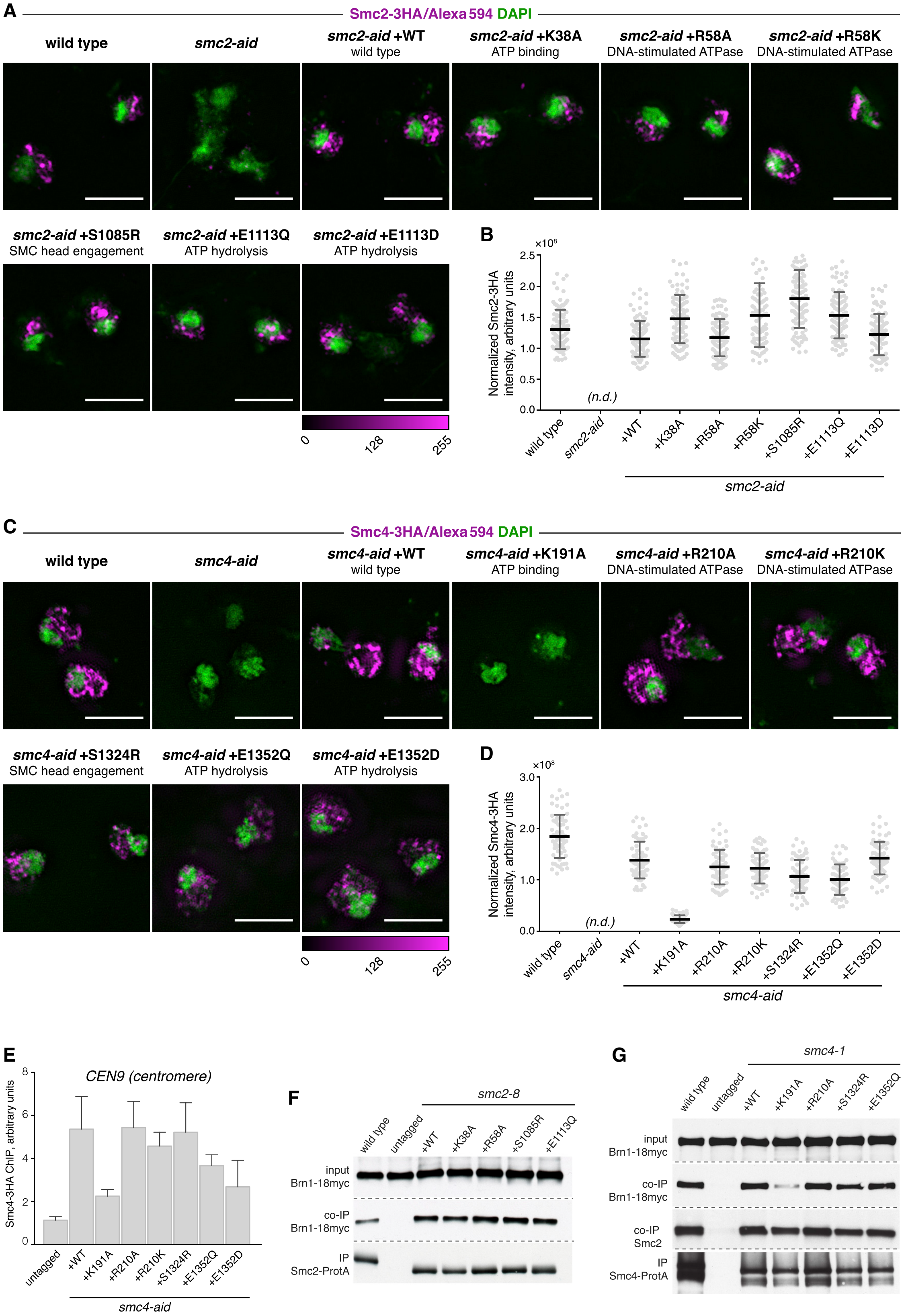

(legend on next page)

viability, which is restored by ectopic expression of wild-type Smc2 or Smc4 (Figure S1). In contrast, most ATPase mutants did not support cell growth, except Smc2 R58K, Smc4 R210K, and Smc4 R210A. This confirmed that like in vertebrates, the ATPase is essential for the function of budding yeast condensin but that certain alterations to the arginine fingers, especially of the Smc4 ATPase, are tolerated. Our findings are consistent with a previous report that ATP binding or head engagement mutants of the budding yeast Smc2 and Smc4 subunits fail to support cell growth (Stray and Lindsley, 2003). Quantitative western blotting confirmed that all ATPase mutant variants were expressed as full-length proteins, albeit at levels somewhat reduced compared to the wild-type control (Figures S2C and S2F).

Condensin Associates with Chromosomes Independently of Its ATPase

We next examined the relationship between condensin's ATPase and chromosome binding. Following endogenous Smc2 depletion and cell synchronization in mitosis (Figure S2A), we visualized chromosome association of ectopic Smc2 by spreading yeast chromosomes on glass slides, followed by antibody staining against its C-terminal 3HA epitope tag. Soluble nuclear content is lost during this procedure (Loidl et al., 1991), and only chromatin-bound Smc2 is expected to be detected. A strain in which endogenous Smc2 was tagged with an equivalent 3HA epitope tag (wild-type) and a strain in which Smc2 was depleted (*smc2-aid*) served as positive and negative controls, confirming staining specificity. The antibody signal was readily detected in wild-type condensin and appeared strongest in a crescent-shaped area of the chromatin mass that stained weakly with the DNA dye DAPI (Figure 1A). This is characteristic of the known enrichment of condensin at the budding yeast rDNA (Freeman et al., 2000). Quantification of the staining intensities, normalized to cellular expression levels, showed that not only ectopic wild-type Smc2 but also all ATPase mutant Smc2 variants associated with chromosomes. Their levels showed some variation but were largely comparable to endogenous Smc2 (Figures 1A and 1B). The same conclusion was reached when we analyzed the raw Smc2 staining intensities (Figure S2B). Thus, an active Smc2 ATPase appears to be dispensable for condensin binding to chromosomes.

We performed a similar analysis for the Smc4 ATPase (Figures S2D–S2F). This again revealed that most Smc4 ATPase mutant proteins bound to DNA at levels comparable to those of wild-type Smc4 (Figures 1C and 1D). As a striking exception, Smc4 K191A, carrying a Walker A motif mutation that is predicted to prevent ATP binding, was undetectable on chromosomes. Chromosome spreads report mainly on condensin that is bound to the rDNA. To extend the analysis to non-rDNA loci and to repeat it using a complementary technique, we examined the chromosomal binding of Smc4 ATPase mutants by chromatin immunoprecipitation, followed by qPCR. We chose the *CEN9* centromeric locus, which is known to be enriched for condensin (D'Ambrosio et al., 2008). This confirmed chromosome association of most Smc4 ATPase mutants, but not the Smc4 K191A mutant, which was detected at levels close to that of a negative control (Figure 1E). Binding of the Smc4 ATP hydrolysis mutants E1352Q and E1352D also appeared somewhat reduced, a possible consequence of their reduced expression levels (Figures S2E and S2F). We obtained similar results at three condensin binding sites along chromosome arms, a tRNA gene and two ribosomal protein gene promoters, although condensin was only weakly detected at these sites (Figure S3).

Loss of Smc4 K191A from chromosomes could result from either impaired chromosome binding or defective condensin complex assembly. To distinguish between these possibilities, we tested the integrity of condensin complexes containing ATPase mutant Smc2 and Smc4 subunits. We fused wild-type and ATPase mutant Smc subunits to a Protein A tag for pull-down and analyzed coprecipitation of the Brn1 subunit by western blotting. All ATPase mutant Smc2 subunits coprecipitated Brn1 at levels equal to those of wild-type Smc2 (Figure 1F). Most Smc4 ATPase mutants also efficiently coprecipitated Brn1 (Figure 1G), with the exception of Smc4 K191A, whose interaction with Brn1 was markedly reduced. This suggests that Smc4 ATP binding is required for stable condensin complex assembly. In the case of cohesin, a similar ATP requirement for Smc1 subunit interaction with the Scc1 kleisin was observed (Arumugam et al., 2003; Weitzer et al., 2003). While we do not yet know how the Smc4 (or Smc1) ATP binding site mutation weakens the kleisin interaction, the loss of Smc4 K191A from

Figure 1. ATPase-Independent Chromosome Binding of Condensin

(A) Chromosome spreads of wild-type or *smc2-aid* cells in metaphase expressing ectopic 3HA-tagged wild-type or ATPase mutant Smc2. Cells were synchronized in G1 with α factor and released into a nocodazole-induced metaphase arrest. Chromosome spreads were stained with DAPI and anti-HA/Alexa Fluor 594 antibodies. Scale bars represent 4 μm.

(B) Quantification of the Smc2-3HA staining intensities in (A), normalized by cellular expression levels assessed by immunoblotting. Error bars represent mean $\pm$ SD ($n \geq 92$).

(C) Chromosome spreads of wild-type or *smc4-aid* cells in metaphase expressing ectopic 3HA-tagged wild-type or ATPase mutants of Smc4, as in (A).

(D) Quantification of the Smc4-3HA staining intensities in (C), normalized by cellular expression levels assessed by immunoblotting. Error bars represent mean $\pm$ SD ($n \geq 63$).

(E) Chromatin immunoprecipitation (ChIP)-qPCR signal of Smc4-3HA at *CEN9*, normalized to a negative binding site, in *smc4-aid* cells in metaphase expressing ectopic wild-type or ATPase mutants of Smc4. Error bars represent mean $\pm$ SEM ($n = 3$).

(F) Coimmunoprecipitation of Brn1-18myc with ATPase mutants of Smc2, as assessed following their immunoprecipitation by means of a Protein A tag in a temperature-sensitive *smc2-8* background.

(G) Coimmunoprecipitation of Brn1-18myc with ATPase mutants of Smc4, as assessed following their immunoprecipitation by means of a Protein A tag in a temperature-sensitive *smc4-1* background.

See also Figure S1 for a viability assay of cells harboring condensin SMC ATPase mutations; Figure S2 for confirmation of cell-cycle synchrony, raw staining intensities, and protein expression levels used for normalization; and Figure S3 for ChIP-qPCR analyses at additional loci.

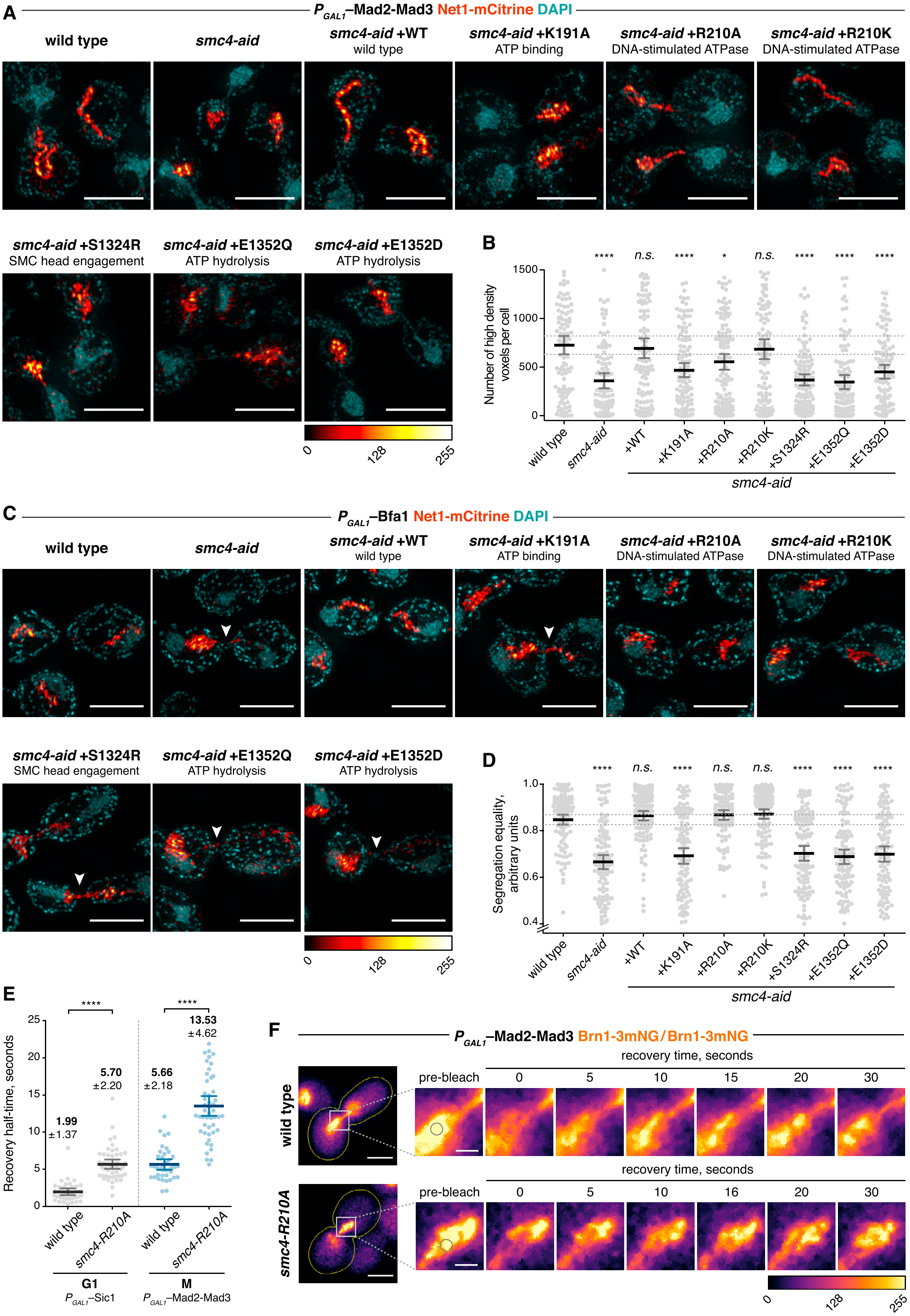

(legend on next page)

chromosomes is likely a consequence of its inability to form a stable condensin complex.

Our observation that budding yeast condensin associates with chromosomes independently of a functional ATPase is broadly consistent with results from vertebrate condensin (Hudson et al., 2008; Kinoshita et al., 2015). Our observations differ with respect to Walker A motif mutations. Vertebrate condensin containing both Smc4 and Smc2 Walker A motif mutations also fails to gain stable chromosome binding. However, in contrast to our observations, these mutant Smc subunits appear to be part of a stable condensin complex. The exact consequences of ATP binding on subunit interactions, complex stability, and chromosome recruitment thus remain to be explored. Condensin is thought to be targeted to chromosomes by interactions with transcription factors (Haeusler et al., 2008; Iwasaki et al., 2015). Physical interactions with such targeting components might provide ATPase-independent chromatin recruitment. Direct DNA contacts of condensin's HEAT repeat subunits could also contribute to its recruitment (Piazza et al., 2014). We expect that subsequent topological loading of condensin onto DNA requires ATP hydrolysis.

ATP Hydrolysis Controls rDNA Condensation and Segregation

We next assessed the effect of condensin ATPase mutations on chromosome condensation. The rDNA array on the right arm of budding yeast chromosome XII is a condensin-rich, approximately 1 Mb long, well-characterized model locus for chromosome condensation (Freeman et al., 2000; Lavoie et al., 2004; Sullivan et al., 2004). We visualized the locus by fusing the rDNA binding protein Net1 to the yellow fluorescent protein mCitrine (Griesbeck et al., 2001) and used three-dimensional structured illumination microscopy (SIM) to capture high-resolution images of a fluorescent-conjugated GFP nanobody bound to Net1-mCitrine (Ries et al., 2012). We adapted a previously reported method to extract the high-frequency, sub-diffraction information provided by SIM from these images and automatically determined an intensity threshold for each cell (Marbouty et al., 2015; see Supplemental Experimental Procedures for details). This allowed us to count the number of high-density volumetric pixels per cell as a high-resolution measure of chromosome condensation. An exploratory analysis revealed a measurable difference in condensation between G1 and mitotic cells, validating the approach (Figure S4A).

Following release from synchronization with pheromone α factor treatment in G1, we overexpressed Mad2-Mad3, a fusion protein of two mitotic checkpoint proteins, from the galactose-inducible *GAL1* promoter to achieve uniform mitotic arrest (Figure S4B) (Lau and Murray, 2012). Auxin was added at the time of G1 release to deplete endogenous Smc4. This allowed us to assess the ability of ectopic wild-type or ATPase mutant Smc4 to support chromosome condensation. Most Smc4 ATP binding and hydrolysis mutations were unable to support chromosome condensation over what is seen in the absence of Smc4, as evident by the failure to generate high-density Net1 signals in mitosis (Figures 2A and 2B). Only the mild arginine finger R210K mutation supported chromosome condensation and, to a smaller extent, the R210A variant. This confirms that ATP hydrolysis is instrumental for chromosome condensation. The intermediate condensation defect caused by a mild ATPase mutation suggests that an ATP hydrolysis-dependent step might be rate limiting for chromosome condensation.

Besides chromosome condensation, condensin is crucial for sister chromatid resolution. Anaphase bridges and consequent chromosome missegregation are characteristic features of mitosis with compromised condensin function. We therefore assessed the fidelity of rDNA segregation as a measure for condensin function. We synchronized cells in late anaphase by overexpression of the mitotic exit inhibitor Bfa1 (Figure S4C) (Li, 1999), and we recorded rDNA segregation equality as the ratio of Net1-mCitrine signals in the two cell halves (the lower signal divided by the higher). In wild-type cells, the rDNA equally segregated into the two daughter cells (Figures 2C and 2D). Following Smc4 depletion, rDNA bridges often remained visible and unequal segregation was evident. Segregation equality was reestablished by expression of wild-type Smc4 and by the two arginine finger mutants R210A and R210K, but not by any other ATPase mutants. These results confirm that condensin's ATPase is instrumental for its function in chromosome segregation. We note a subtle difference in the effect of individual ATPase mutations on chromosome condensation and sister chromatid segregation. Smc4 R210A was significantly impaired in promoting rDNA condensation but proficient in securing its equal segregation (compare Figures 2B and 2D), which may explain the viability

Figure 2. SMC ATPase Controls Chromosome Condensation, Segregation, and Condensin Turnover

(A) Metaphase rDNA morphology of Smc4 ATPase mutants visualized by structured illumination microscopy of the rDNA binding protein Net1. Cells were synchronized in G1 with α factor and released into a metaphase arrest due to overexpression of a Mad2-Mad3 fusion protein. Scale bars represent 4 μm.

(B) Quantification of rDNA condensation, measured by the number of high-density volumetric pixels in the high-frequency data of structured illumination images. Error bars represent mean $\pm$ 95% confidence interval ($n \geq 90$); asterisks denote p values with respect to the first column (NS, not significant; *$p < 0.05$, **$p < 0.01$, ***$p < 0.001$, ****$p < 0.0001$; ordinary one-way ANOVA, Dunnett's multiple comparison test).

(C) rDNA segregation in Smc4 ATPase mutants visualized by structured illumination microscopy of the rDNA binding protein Net1. Cells were synchronized in G1 and released into a late anaphase arrest due to overexpression of Bfa1. Arrowheads indicate chromatin bridges. Scale bars represent 4 μm.

(D) Quantification of rDNA segregation as the ratio of Net1 fluorescence intensity in the two cell halves. A ratio of 1 indicates equal segregation. Error bars represent mean $\pm$ 95% confidence interval ($n \geq 106$); asterisks denote p values as in (B).

(E) Fluorescence recovery half-times, following photobleaching, of the Brn1-3mNeonGreen signal in homozygous diploid wild-type and *smc4-R210A* cells, arrested in G1 by overexpression of Sic1 or metaphase by overexpression of Mad2-Mad3. Error bars represent mean $\pm$ SD ($n \geq 37$) (ordinary one-way ANOVA, Sidak's multiple comparison test).

(F) Examples of fluorescence recovery of the Brn1-3mNeonGreen signal in wild-type and *smc4-R210A* cells (outlined) in metaphase, with the area around the bleach spot (indicated by circles) magnified for clarity. Scale bars for whole-cell images represent 4 μm; those for the inset represent 1 μm.

See also Figure S4 for illustration of the high-density voxel chromosome condensation assay and confirmation of the cell-cycle arrests.

of this ATPase mutant (Figure S1B). This difference is compatible with the idea that chromosome condensation and sister chromatid resolution are separable activities of the condensin complex (D'Amours et al., 2004; Sullivan et al., 2004). We cannot, however, exclude the possibility that condensation is merely delayed in the Smc4 R210A mutant and reaches wild-type levels later in anaphase, allowing equal rDNA segregation.

Cell-Cycle-Regulated Condensin Dynamics, Controlled by Its ATPase

Having confirmed the importance of the budding yeast condensin ATPase, we asked whether ATP hydrolysis affects the dynamic chromosome binding behavior of condensin. We fused three tandem copies of mNeonGreen (Shaner et al., 2013) to the C terminus of the endogenous Brn1 subunit. This yielded a bright condensin signal in the yeast nucleus that allowed us to perform fluorescence recovery after photobleaching (FRAP) experiments. We used homozygous diploid strains for these experiments, whose larger nuclear area facilitated the fluorescence recordings.

To investigate whether the dynamic turnover of budding yeast condensin on chromosomes changes between interphase and mitosis, we arrested cells in late G1 by overexpression of the Cdk inhibitor Sic1 (Figure S4D) (Lopez-Serra et al., 2013) or in mitosis by overexpression of Mad2-Mad3. Fluorescence recovery of a bleached region in G1 was very fast, with a recovery half-time of 1.99 $\pm$ 1.37 s (mean $\pm$ SD). In mitosis, the recovery half-time almost tripled to 5.66 $\pm$ 2.18 s (Figures 2E and 2F), suggesting reduced turnover of chromosome-bound condensin in mitosis. A mitotic recovery half-time of 5.66 s in our experiments is compatible with a fluorescent decay constant derived from a fluorescence loss in photobleaching experiment of around 8 s (Robellet et al., 2015). Another study reported a markedly lower rate of condensin turnover in mitotic budding yeast cells (Lawrimore et al., 2015). We do not know the reason for this difference. While our measurements revealed an overall faster turnover of yeast condensin compared to human condensin (Gerlich et al., 2006), the relative stabilization of chromosome binding in mitosis appears to be a conserved feature that characterizes condensin function in both organisms.

We next addressed whether chromosome binding dynamics of the condensin complex are controlled by its ATPase. If this was the case, we would expect slower condensin turnover if the ATPase is compromised. We chose to investigate this using Smc4 R210A, which supports cell viability with reduced rDNA condensation. We created a yeast strain in which endogenous Smc4 was replaced with Smc4 R210A and repeated the FRAP experiments to determine condensin turnover. The condensin residence time on chromosomes increased roughly 3-fold, both in interphase and in mitosis (Figures 2E and 2F). This suggests that ATP hydrolysis regulates condensin's residence time on chromosomes and that an ATP hydrolysis-dependent step, possibly DNA entry or exit from the condensin ring, correlates with its turnover.

Cell-Cycle Regulation of Condensin Dynamics by Smc4 Phosphorylation

The budding yeast Smc4 subunit is a target for Cdk phosphorylation (Robellet et al., 2015). Mass spectrometric analysis of Smc4, immunopurified from mitotically arrested cells, confirmed phosphorylation of a cluster of Cdk consensus sites close to the Smc4 N terminus. These lie within an N-terminal extension that precedes the Smc4 ATPase head domain (Figures 3A and S5). We could not detect phosphorylation of additional Cdk consensus sites within Smc4 that have previously been invoked in condensin regulation (Robellet et al., 2015). Smc4 shows little electrophoretic mobility change during cell-cycle progression. To analyze the timing of Smc4 phosphorylation during the cell cycle, we therefore immunoprecipitated Smc4 from aliquots of a culture that passed through a synchronous cell cycle and probed Smc4 using a phospho-Cdk substrate antibody (Figures 3B and 3C). While Smc4 levels were constant throughout the cell cycle, reactivity of the phospho-Cdk substrate antibody increased following S phase and peaked during mitosis. This suggests that Smc4 undergoes mitosis-specific Cdk phosphorylation in budding yeast.

To test whether Cdk phosphorylation controls the stabilization of condensin on mitotic chromosomes, we initially employed a *smc4-7A* allele in which the 7 Cdk consensus recognition sites in the Smc4 N terminus have been replaced by alanines. As reported (Robellet et al., 2015), *smc4-7A* cells show defective chromosome condensation (Figure 3D). However, rDNA compaction was reduced in *smc4-7A* cells not only in mitosis but also in G1 arrested cells, when Smc4 is not expected to be phosphorylated (Figure 3E). This suggests that the Smc4-7A protein is defective in ways additional to being refractory to Cdk phosphorylation. This conclusion was corroborated when we measured chromosome binding dynamics of condensin. Non-phosphorylatable Smc4-7A would be expected to maintain fast interphase turnover kinetics even in mitosis. In contrast to this expectation, we found that Smc4-7A showed slower recovery times than wild-type condensin, both in interphase and in mitosis (Figure 3F). This suggests that the Smc4 N-terminal extension plays a role in condensin function but that the Smc4-7A phenotype goes beyond being non-phosphorylatable. In addition, Smc4-7A condensin turnover retained aspects of cell-cycle regulation, being more stable on chromosomes in mitosis than in interphase. Thus, levels of mitotic condensin regulation exist that go beyond Cdk phosphorylation of Smc4.

Given the difficulty with the interpretation of results obtained with the *smc4-7A* allele, we aimed to create a gain-of-function *SMC4* allele that is phosphorylated prematurely. We fused Smc4 to the mitotic cyclin Clb2 (Smc4-Clb2), with the expectation that this recruits Cdk activity to Smc4 and leads to its phosphorylation even in interphase. Clb2 was stripped of localization and degradation signals to avoid unwanted interference with Smc4 function. This approach was previously successful with achieving constitutive phosphorylation of several other mitotic regulators (Kuilman et al., 2015). As a control, we generated a fusion of Smc4 with a Clb2 variant that is defective in recruiting Cdk (Smc4-Clb2$^{\Delta Cdk}$) (Figure 4A). We arrested cells in G1 with the expectation that condensin turnover should be reduced in Smc4-Clb2 cells if Smc4 phosphorylation regulates condensin turnover. The recovery half-life of the Smc4-Clb2$^{\Delta Cdk}$ condensin fusion was close to that of wild-type condensin, confirming that the fusion construct did not interfere with normal condensin function. However, the increase in recovery half-time of the

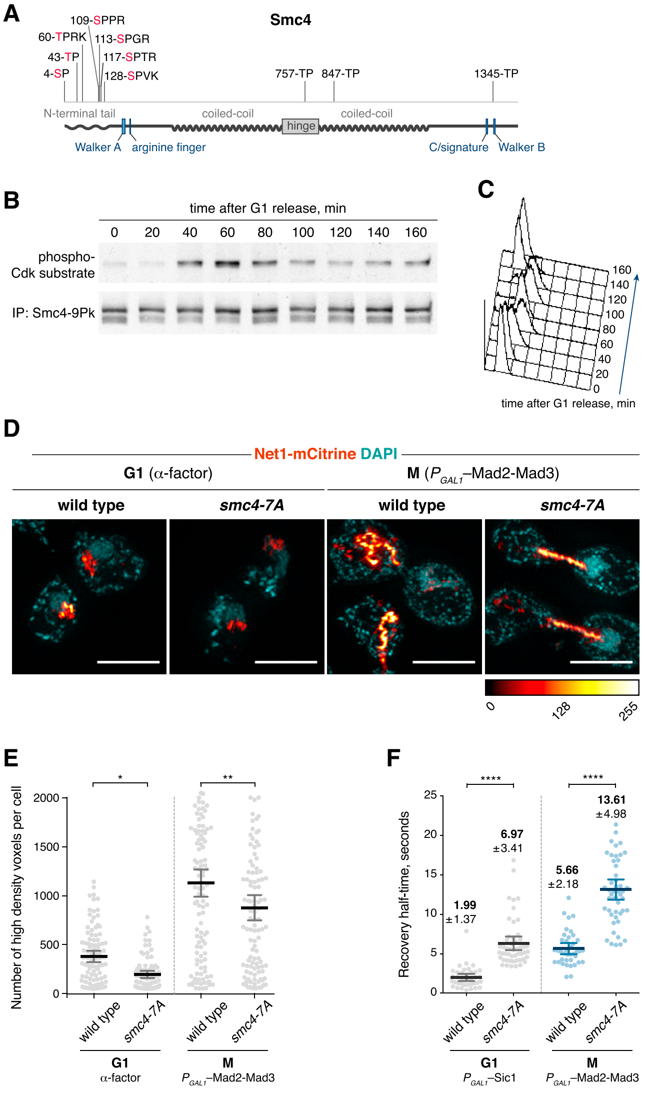

Figure 3. Phosphosite Mutant Smc4 Affects Condensin Dynamics and Chromosome Condensation

(A) Schematic representation of Smc4, showing the seven N-terminal Cdk phosphorylation sites.

(B) Time course of Smc4 phosphorylation after release from an α factor-induced G1 arrest, assessed by Smc4 immunoprecipitation and probing with an anti-phospho-Cdk substrate antibody.

(C) Flow cytometry profiles of DNA content in cells assessed in (B).

(D) rDNA morphology of wild-type and *smc4-7A* cells in G1 or metaphase, visualized by structured illumination microscopy of the rDNA binding protein Net1. Cells were synchronized in G1 with α factor and released into a metaphase arrest. Scale bars represent 4 μm.

(E) Quantification of rDNA condensation in *smc4-7A* cells compared to wild-type in both G1 and metaphase. The means and 95% confidence intervals are presented (n ≥ 80) (ordinary one-way ANOVA, Sidak's multiple comparison test).

(F) Fluorescence recovery half-times, following photobleaching, of the Brn1-3mNeonGreen signal in homozygous diploid wild-type and *smc4-7A* cells arrested in G1 by overexpression of Sic1 or metaphase by overexpression of Mad2-Mad3. Error bars represent mean ± SD (n ≥ 34) (ordinary one-way ANOVA, Sidak's multiple comparison test).

See also Figure S5 for the identification of Smc4 phosphorylation sites.

Smc4-Clb2 condensin fusion was only marginal and not significant (Figure 4B). We surmised that overexpression of the Cdk inhibitor Sic1, which we used to produce a G1 arrest in our diploid cells, might compromise the ability of the Clb2 fusion to phosphorylate Smc4. Alternatively, the G1 arrest state might be unconducive to chromosome condensation for additional reasons.

To overcome the need for a G1 arrest, we analyzed condensin dynamics in an asynchronously growing cell population. We selected interphase cells for experiments based on their small bud size (in a post-experiment analysis, the ratio of daughter-to-mother cell area was 0.15 ± 0.04, mean ± SD). This includes cells in late G1, S, and early G2. The recovery half-time of the Smc4-Clb2$^{\Delta Cdk}$ condensin fusion remained close to what we previously observed in G1 arrested cells, suggesting that little change to condensin dynamics occurs while cells progress through interphase. In contrast, the recovery half-time of the Smc4-Clb2 condensin fusion almost doubled, suggesting that Cdk phosphorylation contributes to a slowdown of condensin turnover on chromosomes (Figure 4B). The recovery half-time of Smc4-Clb2 in interphase was less than that observed in mitosis, consistent with the possibility that regulation, in addition to Smc4 phosphorylation, affects condensin in mitosis. This might, for example, include phosphorylation by Polo kinase (St-Pierre et al., 2009), which is not fully active in interphase. In mitotically arrested cells, Smc4-Clb2 also turned over more slowly than Smc4-Clb2$^{\Delta Cdk}$, which could be explained if Smc4 is incompletely phosphorylated during normal mitosis and additional phosphorylation due to the Smc4-Clb2 fusion is able to further dampen condensin turnover. As a control so that the effect of Clb2 fusion is mediated by Smc4 phosphorylation, we also fused Clb2 to Smc4-7A. While Smc4-7A condensin turns over more slowly, its behavior remained unchanged in response to Clb2 fusion (Figure S6A). This supports the idea that the Clb2 fusion exerts its effect by N-terminal Smc4 phosphorylation and therefore that Smc4 phosphorylation contributes to regulating condensin turnover on chromosomes.

We confirmed that Smc4-Clb2 fusion led to increased Smc4 phosphorylation by probing immunoprecipitated Smc4 and Smc4-Clb2 with a phospho-Cdk substrate antibody (Figure S6B). We observed increased reactivity with the antibody following Clb2 fusion, suggestive of increased phosphorylation. Clb2 fusion also led to a greater phosphorylation signal in G1 arrested cells and of the Smc4-7A protein, albeit to a lesser extent compared to wild-type Smc4 in mitosis. This suggests that Clb2 fusion leads to phosphorylation of additional sites on Smc4-7A, while functionally important sites lie within the Smc4 N terminus.

Chromosome Condensation Requires Tuned Condensin Turnover

Finally, we addressed whether reduced condensin turnover following Smc4-Clb2 fusion leads to premature chromosome condensation. To analyze this, we arrested haploid cells in G1 by α factor treatment. Smc4-Clb2 caused a marginal, but not statistically significant, increase in rDNA compaction (Figures S6C and S6D). When we arrested cells in mitosis, we found that Clb2 fusion compromised rDNA condensation. As a control, Clb2$^{\Delta Cdk}$ fusion did not compromise condensation, excluding a non-specific effect of the fusions. While somewhat

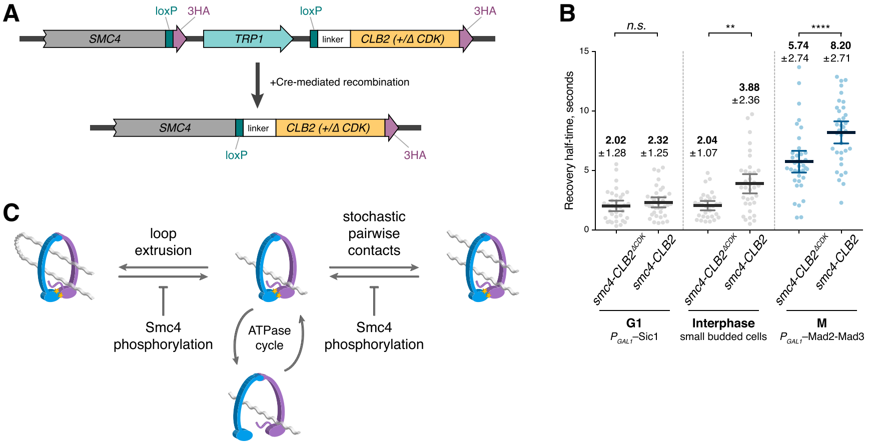

Figure 4. Smc4 Phosphorylation Regulates Condensin Dynamics and Chromosome Condensation

(A) Schematic representation of the gene loci expressing Smc4-Clb2 and Smc4-Clb2$^{\Delta Cdk}$ fusion proteins.

(B) Fluorescence recovery half-times, following photobleaching, of the Brn1-3mNeonGreen signal in homozygous diploid *SMC4-CLB2*$^{\Delta CDK}$ and *SMC4-CLB2* cells in interphase and metaphase. Error bars represent mean ± SD ($n \geq 31$) (ordinary one-way ANOVA, Sidak's multiple comparison test).

(C) Model of chromosome condensation driven by cell-cycle-regulated Smc4 phosphorylation, resulting in stabilization of condensin-DNA binding. This promotes chromosome compaction if the condensation reaction proceeds by either diffusion capture or loop extrusion.

See also Figure S6 for additional condensin dynamics, phosphorylation, and chromosome condensation assays.

counter-intuitive at first glance, this observation is reminiscent of Smc4 R210A cells in which reduced condensin turnover led to compromised chromosome condensation rather than hypercompaction. Thus, slowing condensin turnover beyond its normally observed rates impairs rather than increases chromosome condensation.

DISCUSSION

We have shown that dynamic condensin binding to chromosomes is controlled by the condensin ATPase and by cell-cycle-dependent phosphorylation. Mitotic chromosome condensation correlates with a slowdown of condensin turnover, mediated by mitotic phosphorylation. However, reduction of turnover by hyperphosphorylation or an ATPase mutation does not increase condensation. Rather, our observations suggest that chromosome compaction requires an optimum rate of condensin turnover. We imagine that at this rate, intra-chromosomal contacts are established at a sufficient rate and maintained for an adequate length of time.

The idea that chromosome condensation requires an optimum condensin turnover rate can apply to either of two proposed chromosome compaction models: diffusion capture or loop extrusion (Alipour and Marko, 2012; Cheng et al., 2015; Fudenberg et al., 2016). The first model proposes that condensin stabilizes stochastic encounters between its binding sites, either by trapping more than one DNA within one condensin ring or by interactions between more than one condensin. Here, the establishment of productive DNA-DNA interactions likely requires multiple rounds of ATP hydrolysis to entrap more than one DNA duplex in the condensin ring, followed by a sufficiently long retention period. If both DNA entry and DNA exit are ATP hydrolysis-dependent reactions, as is the case for the cohesin ring (Murayama and Uhlmann, 2015), a fine balance between DNA entry and retention must be achieved. The alternative model of loop extrusion proposes that compaction proceeds by threading a DNA loop through condensin and enlarging it. In this case, ATP hydrolysis may drive both DNA entry into the condensin ring and subsequent DNA translocation (Ganji et al., 2018). A balance between loop initiation and the processivity of extrusion must be found to reach compaction.

Cdk phosphorylation in both budding and fission yeast targets an N-terminal Smc4 extension. In fission yeast, this increases the nuclear import of condensin (Sutani et al., 1999). Whether it also changes the dynamic behavior of fission yeast condensin is not yet known. In vertebrates, mitotic Cdk phosphorylation of condensin I and II occurs on C-terminal parts of the CAP-D2 and CAP-D3 subunits, respectively (Abe et al., 2011; Kimura et al., 1998). If HEAT repeat subunit topology is similar between cohesin and condensin (Lee et al., 2016), these regions might lie close to the SMC4 N terminus. In this way, Cdk phosphorylation could in all cases add negative charge to a related part of the condensin complex, paving the way for a conserved mechanism of control. Phosphorylation could lead to electrostatic repulsion of DNA, potentially altering how DNA engages with the complex. Alternatively, phosphorylation could induce conformational changes that alter condensin behavior. Further biochemical investigations will be required to understand how condensin promotes DNA condensation and how control of its dynamic behavior by posttranslational modifications regulates this essential cell biological process.

EXPERIMENTAL PROCEDURES

Additional details are available in the Supplemental Experimental Procedures.

Chromosome Spreads

We prepared chromosome spreads as previously described (Loidl et al., 1991) but adapted the procedure for multiwell slides. We resuspended 3×10^7 cells in 1 mL of S1 (100 mM potassium phosphate buffer [pH 7.4], 0.5 mM $MgCl_2$, 1.2 M sorbitol). Cells were spheroplasted by incubation at 37°C for 20 min in S1 containing 20 mM DTT and 140 μg/mL of zymolase T-100. We halted cell wall digestion by addition of 1 mL of ice-cold S3 (0.1 M 2-(N-morpholino)ethanesulfonic acid, 1 mM EDTA, 0.5 mM $MgCl_2$, 1 M sorbitol [pH 6.4]) and resuspended washed spheroplasts in 200 μL of S3. For spreading, we rapidly pipetted onto each chamber of a multiwell slide 2 μL of fixative (4% paraformaldehyde, 3.4% sucrose, 0.2 mM NaOH), 2 μL of spheroplast suspension, 4 μL of 1% lipsol in water, and 4 μL of fixative. Slides were dried overnight

before immunostaining (rat anti-hemagglutinin [anti-HA] 3F10, 1:500; Alexa Fluor 594 anti-rat, 1:1,000), followed by DAPI staining and SIM.

Cell Fixation and Nanobody Staining

We fixed ~3 × 10^8 cells arrested in G1 (α factor), metaphase (P_{GAL1}-Mad2-Mad3), or anaphase (P_{GAL1}-Bfa1) by addition of 3.6% methanol-free, electron microscopy (EM)-grade formaldehyde to the culture medium. After incubation at room temperature for 15 min, we halted fixation by washing cells in TBS (150 mM NaCl, 50 mM Tris-HCl [pH 8.0]) containing 50 mM NH_4Cl and then 3× with TBS. Cells were stained with Atto 594-conjugated GFP booster nanobody at 4°C overnight and DAPI and then imaged by SIM using an API OMX v3 microscope.

FRAP

Brn1-3mNeonGreen diploid cells were grown in rich yeast peptone (YP) medium supplemented with 2% raffinose. Cell-cycle arrests were performed for 3–4.5 hr by addition of 2% galactose directly to asynchronous cultures. Cells were then harvested and resuspended in synthetic yeast nitrogen base (YNB) medium supplemented with 2% raffinose + 2% galactose. Cells were then applied to a 1.2% agarose-medium pad for imaging. FRAP experiments were performed on a Zeiss LSM 880 confocal microscope with 488 nm laser excitation and >505 nm longpass emission settings. We typically acquired 3 pre-bleach frames at 0.5%–1% power, bleached a circular spot of 9 pixel/0.59 μm diameter with 5 iterations at 50% power, and monitored recovery every 1–3 s at 0.5%–1% power. We used fluorescent regions from adjoining cells in the same field to correct for general photobleaching and used Zen software (Zeiss) to fit a single exponential recovery curve. Double exponential curves did not improve the fit.

Statistical Methods

Statistical analyses were performed in GraphPad Prism. We used an ordinary one-way ANOVA with Dunnett's test to compare multiple samples to a single control or the Sidak method to compare selected sets of means. For single comparisons, we used Student's unpaired t test with equal SD.

SUPPLEMENTAL INFORMATION

Supplemental Information includes Supplemental Experimental Procedures, six figures, and one table and can be found with this article online at https://doi.org/10.1016/j.celrep.2018.04.082.

ACKNOWLEDGMENTS

We acknowledge A. Vaahtokari and M. Renshaw for assistance with SIM; P. Jordan for assistance with confocal microscopy; H. Flynn and M. Skehel for mass spectrometry analysis; A. Donaldson, J. Vogel, and Y. Barral for reagents; J. Cooper for her support; and our laboratory members for discussions and critical reading of the manuscript. This work was supported by the European Research Council (grant agreement No 670412) and The Francis Crick Institute, which receives its core funding from Cancer Research UK (FC001198), the UK Medical Research Council (FC001198), and the Wellcome Trust (FC001198). R.T. was supported by a Boehringer Ingelheim Fonds PhD Fellowship. S.H. and S.M. were supported by EMBO Long-Term Fellowships.

AUTHOR CONTRIBUTIONS

R.T. and F.U. conceived the study; R.T. performed all experiments and data analysis, except J.K. performed an initial analysis of the condensin ATPase and its role in condensin complex formation; S.H. identified Smc4 phosphorylation sites and their cell-cycle regulation; and S.M. performed the Smc4 chromatin immunoprecipitation (ChIP)-qPCR experiments. R.T. and F.U. prepared the manuscript.

DECLARATION OF INTERESTS

The authors declare no competing interests.

Received: July 26, 2017
Revised: December 15, 2017
Accepted: April 18, 2018
Published: May 22, 2018

REFERENCES

Abe, S., Nagasaka, K., Hirayama, Y., Kozuka-Hata, H., Oyama, M., Aoyagi, Y., Obuse, C., and Hirota, T. (2011). The initial phase of chromosome condensation requires Cdk1-mediated phosphorylation of the CAP-D3 subunit of condensin II. Genes Dev. *25*, 863–874.

Alipour, E., and Marko, J.F. (2012). Self-organization of domain structures by DNA-loop-extruding enzymes. Nucleic Acids Res. *40*, 11202–11212.

Arumugam, P., Gruber, S., Tanaka, K., Haering, C.H., Mechtler, K., and Nasmyth, K. (2003). ATP hydrolysis is required for cohesin's association with chromosomes. Curr. Biol. *13*, 1941–1953.

Cheng, T.M.K., Heeger, S., Chaleil, R.A.G., Matthews, N., Stewart, A., Wright, J., Lim, C., Bates, P.A., and Uhlmann, F. (2015). A simple biophysical model emulates budding yeast chromosome condensation. eLife *4*, e05565.

D'Ambrosio, C., Schmidt, C.K., Katou, Y., Kelly, G., Itoh, T., Shirahige, K., and Uhlmann, F. (2008). Identification of cis-acting sites for condensin loading onto budding yeast chromosomes. Genes Dev. *22*, 2215–2227.

D'Amours, D., Stegmeier, F., and Amon, A. (2004). Cdc14 and condensin control the dissolution of cohesin-independent chromosome linkages at repeated DNA. Cell *117*, 455–469.

Freeman, L., Aragon-Alcaide, L., and Strunnikov, A. (2000). The condensin complex governs chromosome condensation and mitotic transmission of rDNA. J. Cell Biol. *149*, 811–824.

Fudenberg, G., Imakaev, M., Lu, C., Goloborodko, A., Abdennur, N., and Mirny, L.A. (2016). Formation of chromosomal domains by loop extrusion. Cell Rep. *15*, 2038–2049.

Ganji, M., Shaltiel, I.A., Bisht, S., Kim, E., Kalichava, A., Haering, C.H., and Dekker, C. (2018). Real-time imaging of DNA loop extrusion by condensin. Science *360*, 102–105.

Gerlich, D., Hirota, T., Koch, B., Peters, J.-M., and Ellenberg, J. (2006). Condensin I stabilizes chromosomes mechanically through a dynamic interaction in live cells. Curr. Biol. *16*, 333–344.

Griesbeck, O., Baird, G.S., Campbell, R.E., Zacharias, D.A., and Tsien, R.Y. (2001). Reducing the environmental sensitivity of yellow fluorescent protein. Mechanism and applications. J. Biol. Chem. *276*, 29188–29194.

Haeusler, R.A., Pratt-Hyatt, M., Good, P.D., Gipson, T.A., and Engelke, D.R. (2008). Clustering of yeast tRNA genes is mediated by specific association of condensin with tRNA gene transcription complexes. Genes Dev. *22*, 2204–2214.

Hirano, T. (2016). Condensin-based chromosome organization from bacteria to vertebrates. Cell *164*, 847–857.

Hopfner, K.-P., Karcher, A., Shin, D.S., Craig, L., Arthur, L.M., Carney, J.P., and Tainer, J.A. (2000). Structural biology of Rad50 ATPase: ATP-driven conformational control in DNA double-strand break repair and the ABC-ATPase superfamily. Cell *101*, 789–800.

Hudson, D.F., Ohta, S., Freisinger, T., Macisaac, F., Sennels, L., Alves, F., Lai, F., Kerr, A., Rappsilber, J., and Earnshaw, W.C. (2008). Molecular and genetic analysis of condensin function in vertebrate cells. Mol. Biol. Cell *19*, 3070–3079.

Iwasaki, O., Tanizawa, H., Kim, K.-D., Yokoyama, Y., Corcoran, C.J., Tanaka, A., Skordalakes, E., Showe, L.C., and Noma, K. (2015). Interaction between TBP and condensin drives the organization and faithful segregation of mitotic chromosomes. Mol. Cell *59*, 755–767.

Kimura, K., and Hirano, T. (1997). ATP-dependent positive supercoiling of DNA by 13S condensin: a biochemical implication for chromosome condensation. Cell *90*, 625–634.

Kimura, K., Hirano, M., Kobayashi, R., and Hirano, T. (1998). Phosphorylation and activation of 13S condensin by Cdc2 *in vitro*. Science *282*, 487–490.

Kinoshita, K., Kobayashi, T.J., and Hirano, T. (2015). Balancing acts of two HEAT subunits of condensin I support dynamic assembly of chromosome axes. Dev. Cell *33*, 94–106.

Kubota, T., Nishimura, K., Kanemaki, M.T., and Donaldson, A.D. (2013). The Elg1 replication factor C-like complex functions in PCNA unloading during DNA replication. Mol. Cell *50*, 273–280.

Kuilman, T., Maiolica, A., Godfrey, M., Scheidel, N., Aebersold, R., and Uhlmann, F. (2015). Identification of Cdk targets that control cytokinesis. EMBO J. *34*, 81–96.

Lammens, A., Schele, A., and Hopfner, K.-P. (2004). Structural biochemistry of ATP-driven dimerization and DNA-stimulated activation of SMC ATPases. Curr. Biol. *14*, 1778–1782.

Lau, D.T., and Murray, A.W. (2012). Mad2 and Mad3 cooperate to arrest budding yeast in mitosis. Curr. Biol. *22*, 180–190.

Lavoie, B.D., Hogan, E., and Koshland, D. (2004). *In vivo* requirements for rDNA chromosome condensation reveal two cell-cycle-regulated pathways for mitotic chromosome folding. Genes Dev. *18*, 76–87.

Lawrimore, J., Vasquez, P.A., Falvo, M.R., Taylor, R.M., 2nd, Vicci, L., Yeh, E., Forest, M.G., and Bloom, K. (2015). DNA loops generate intracentromere tension in mitosis. J. Cell Biol. *210*, 553–564.

Lee, B.-G., Roig, M.B., Jansma, M., Petela, N., Metson, J., Nasmyth, K., and Löwe, J. (2016). Crystal structure of the cohesin gatekeeper Pds5 and in complex with kleisin Scc1. Cell Rep. *14*, 2108–2115.

Lengronne, A., McIntyre, J., Katou, Y., Kanoh, Y., Hopfner, K.-P., Shirahige, K., and Uhlmann, F. (2006). Establishment of sister chromatid cohesion at the *S. cerevisiae* replication fork. Mol. Cell *23*, 787–799.

Li, R. (1999). Bifurcation of the mitotic checkpoint pathway in budding yeast. Proc. Natl. Acad. Sci. USA *96*, 4989–4994.

Loidl, J., Nairz, K., and Klein, F. (1991). Meiotic chromosome synapsis in a haploid yeast. Chromosoma *100*, 221–228.

Lopez-Serra, L., Lengronne, A., Borges, V., Kelly, G., and Uhlmann, F. (2013). Budding yeast Wapl controls sister chromatid cohesion maintenance and chromosome condensation. Curr. Biol. *23*, 64–69.

Marbouty, M., Le Gall, A., Cattoni, D.I., Cournac, A., Koh, A., Fiche, J.B., Mozziconacci, J., Murray, H., Koszul, R., and Nollmann, M. (2015). Condensin- and replication-mediated bacterial chromosome folding and origin condensation revealed by Hi-C and super-resolution imaging. Mol. Cell *59*, 588–602.

Murayama, Y., and Uhlmann, F. (2015). DNA entry into and exit out of the cohesin ring by an interlocking gate mechanism. Cell *163*, 1628–1640.

Onn, I., Aono, N., Hirano, M., and Hirano, T. (2007). Reconstitution and subunit geometry of human condensin complexes. EMBO J. *26*, 1024–1034.

Piazza, I., Rutkowska, A., Ori, A., Walczak, M., Metz, J., Pelechano, V., Beck, M., and Haering, C.H. (2014). Association of condensin with chromosomes depends on DNA binding by its HEAT-repeat subunits. Nat. Struct. Mol. Biol. *21*, 560–568.

Ries, J., Kaplan, C., Platonova, E., Eghlidi, H., and Ewers, H. (2012). A simple, versatile method for GFP-based super-resolution microscopy via nanobodies. Nat. Methods *9*, 582–584.

Robellet, X., Thattikota, Y., Wang, F., Wee, T.-L., Pascariu, M., Shankar, S., Bonneil, É., Brown, C.M., and D'Amours, D. (2015). A high-sensitivity phospho-switch triggered by Cdk1 governs chromosome morphogenesis during cell division. Genes Dev. *29*, 426–439.

Shaner, N.C., Lambert, G.G., Chammas, A., Ni, Y., Cranfill, P.J., Baird, M.A., Sell, B.R., Allen, J.R., Day, R.N., Israelsson, M., et al. (2013). A bright monomeric green fluorescent protein derived from *Branchiostoma lanceolatum*. Nat. Methods *10*, 407–409.

St-Pierre, J., Douziech, M., Bazile, F., Pascariu, M., Bonneil, E., Sauvé, V., Ratsima, H., and D'Amours, D. (2009). Polo kinase regulates mitotic chromosome condensation by hyperactivation of condensin DNA supercoiling activity. Mol. Cell *34*, 416–426.

Stray, J.E., and Lindsley, J.E. (2003). Biochemical analysis of the yeast condensin Smc2/4 complex: an ATPase that promotes knotting of circular DNA. J. Biol. Chem. *278*, 26238–26248.

Sullivan, M., Higuchi, T., Katis, V.L., and Uhlmann, F. (2004). Cdc14 phosphatase induces rDNA condensation and resolves cohesin-independent cohesion during budding yeast anaphase. Cell *117*, 471–482.

Sutani, T., Yuasa, T., Tomonaga, T., Dohmae, N., Takio, K., and Yanagida, M. (1999). Fission yeast condensin complex: essential roles of non-SMC subunits for condensation and Cdc2 phosphorylation of Cut3/SMC4. Genes Dev. *13*, 2271–2283.

Thadani, R., Uhlmann, F., and Heeger, S. (2012). Condensin, chromatin crossbarring and chromosome condensation. Curr. Biol. *22*, R1012–R1021.

Uhlmann, F. (2016). SMC complexes: from DNA to chromosomes. Nat. Rev. Mol. Cell Biol. *17*, 399–412.

Weitzer, S., Lehane, C., and Uhlmann, F. (2003). A model for ATP hydrolysis-dependent binding of cohesin to DNA. Curr. Biol. *13*, 1930–1940.

Cell Reports, Volume *23*

Supplemental Information

Cell-Cycle Regulation of Dynamic Chromosome Association of the Condensin Complex

Rahul Thadani, Julia Kamenz, Sebastian Heeger, Sofía Muñoz, and Frank Uhlmann

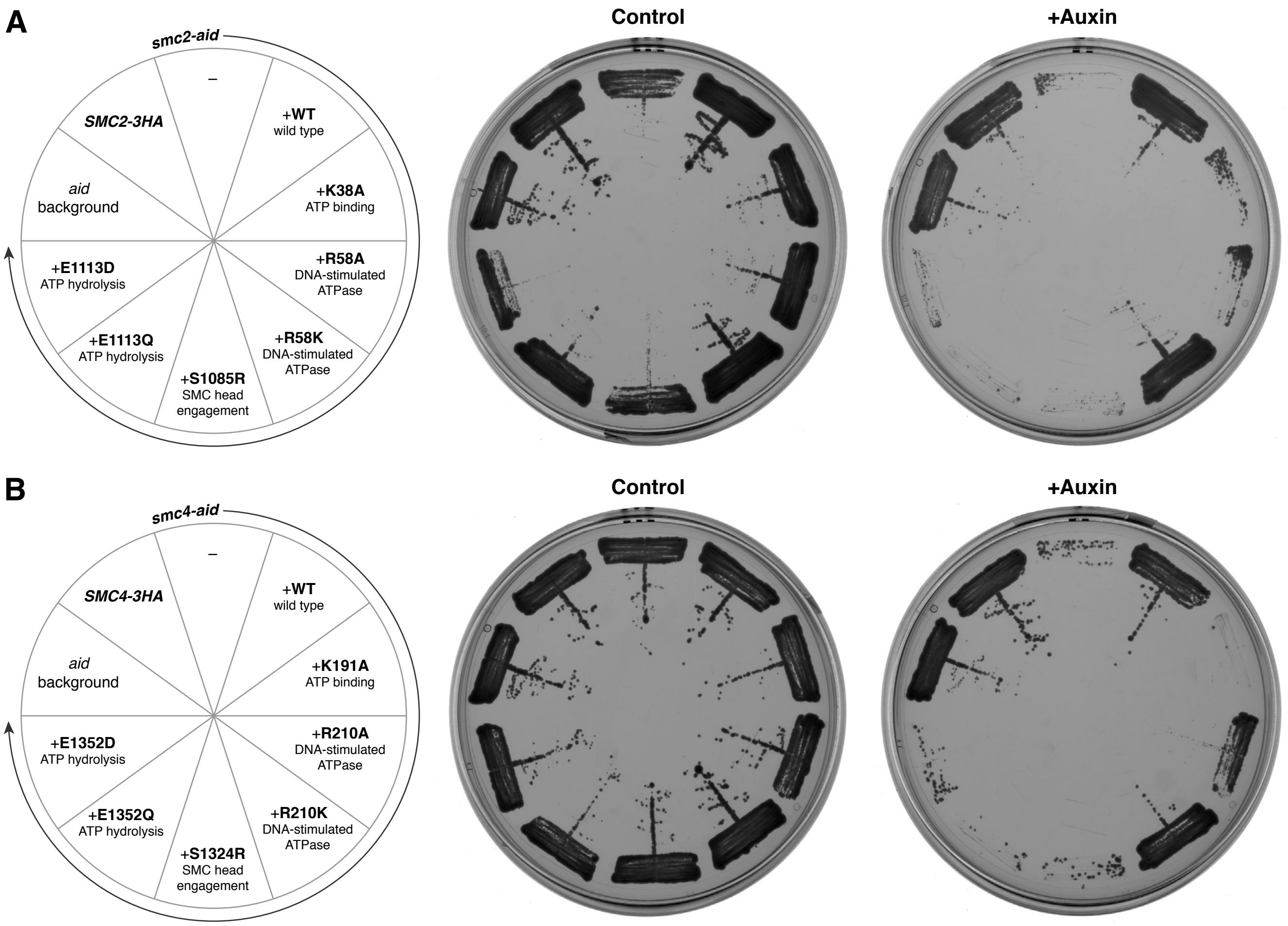

Figure S1. Viability of Condensin SMC ATPase Mutants, Related to Figure 1

(A) Strains expressing epitope-tagged ATPase mutants of Smc2 from an ectopic locus, in an *smc2-aid* background, were streaked onto rich medium containing either methanol as control, or auxin (1 mM IAA). A wild type strain expressing epitope-tagged endogenous Smc2 and the Tir1-expressing *aid* background strain without the *smc2-aid* fusion served as controls. Only wild type Smc2 and Smc2 R58K rescue the conditional lethality of *smc2-aid*.

(B) Strains expressing epitope-tagged ATPase mutants of Smc4 from an ectopic locus, in an *smc4-aid* background, were streaked onto rich medium containing either methanol as control, or auxin (1 mM IAA). A wild type strain expressing epitope-tagged endogenous Smc4 and the Tir1-expressing *aid* background strain without the *smc4-aid* fusion served as controls. Wild type Smc4, Smc4 R210A and Smc4 R210K rescue the conditional lethality of *smc4-aid*.

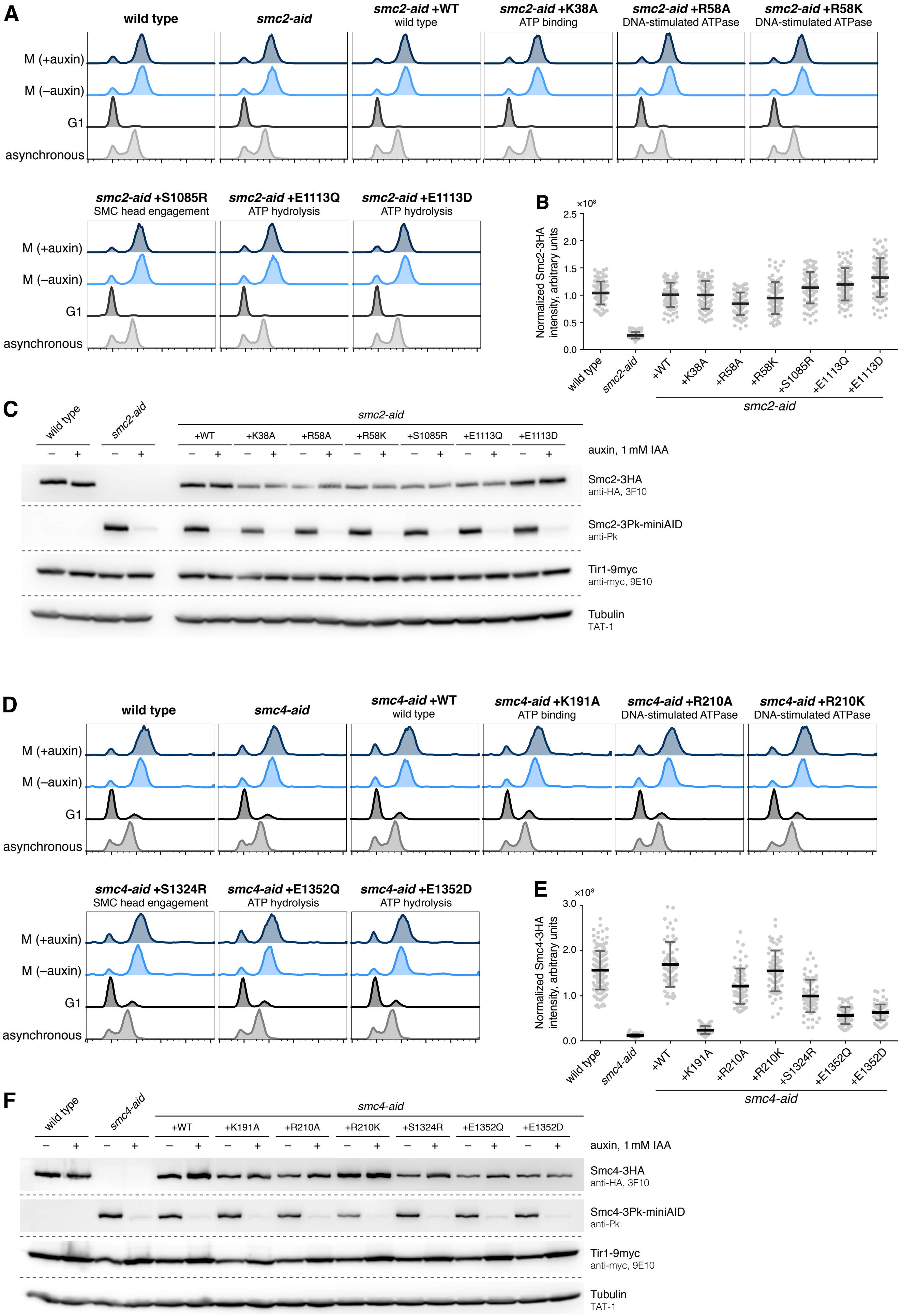
A
wild type
smc2-aid
smc2-aid +WT
wild type
smc2-aid +K38A
ATP binding
smc2-aid +R58A
DNA-stimulated ATPase
smc2-aid +R58K
DNA-stimulated ATPase
M (+auxin)
M (–auxin)
G1
asynchronous
smc2-aid +S1085R
SMC head engagement
smc2-aid +E1113Q
ATP hydrolysis
smc2-aid +E1113D
ATP hydrolysis
B
×10^8
Normalized Smc2-3HA intensity, arbitrary units
2.5
2.0
1.5
1.0
0.5
0.0
wild type
smc2-aid
+WT
+K38A
+R58A
+R58K
+S1085R
+E1113Q
+E1113D
smc2-aid
C
wild type
smc2-aid
smc2-aid
+WT
+K38A
+R58A
+R58K
+S1085R
+E1113Q
+E1113D
auxin, 1 mM IAA
Smc2-3HA
anti-HA, 3F10
Smc2-3Pk-miniAID
anti-Pk
Tir1-9myc
anti-myc, 9E10
Tubulin
TAT-1
D
wild type
smc4-aid
smc4-aid +WT
wild type
smc4-aid +K191A
ATP binding
smc4-aid +R210A
DNA-stimulated ATPase
smc4-aid +R210K
DNA-stimulated ATPase
M (+auxin)
M (–auxin)
G1
asynchronous
smc4-aid +S1324R
SMC head engagement
smc4-aid +E1352Q
ATP hydrolysis
smc4-aid +E1352D
ATP hydrolysis
E
×10^8
Normalized Smc4-3HA intensity, arbitrary units
3.0
2.0
1.0
0.0
wild type
smc4-aid
+WT
+K191A
+R210A
+R210K
+S1324R
+E1352Q
+E1352D
smc4-aid
F
wild type
smc4-aid
smc4-aid
+WT
+K191A
+R210A
+R210K
+S1324R
+E1352Q
+E1352D
auxin, 1 mM IAA
Smc4-3HA
anti-HA, 3F10
Smc4-3Pk-miniAID
anti-Pk
Tir1-9myc
anti-myc, 9E10
Tubulin
TAT-1

Figure S2. Cell Cycle Synchronization and Expression Levels of SMC ATPase Mutants, Related to Figure 1

(A) Flow cytometry profiles of control and Smc2 ATPase mutant strains synchronized in G1 with pheromone α-factor and released into a mitotic arrest (4 µg/ml nocodazole) in the absence (methanol) or presence (1 mM IAA) of auxin.

(B) Raw Smc2-3HA staining intensities from chromosome spreads shown in Figure 1A. Compare to normalized intensities in Figure 1B. Error bars represent mean ± s.d. ($n \geq 92$).

(C) Western blots from the experiment described in (A), showing auxin-induced depletion of Smc2-3Pk-miniAID and expression levels of the 3HA-tagged Smc2 ATPase mutants. Tir1 and tubulin served as loading controls.

(D) Flow cytometry profiles of control and Smc4 ATPase mutant strains treated as in (A).

(E) Raw Smc4-3HA staining intensities from chromosome spreads shown in Figure 1C. Compare to normalized intensities in Figure 1D. Error bars represent mean ± s.d. ($n \geq 63$).

(F) Western blots from the experiment in (D), showing auxin-induced depletion of Smc4-3Pk-miniAID and expression levels of the 3HA-tagged Smc4 ATPase mutants.

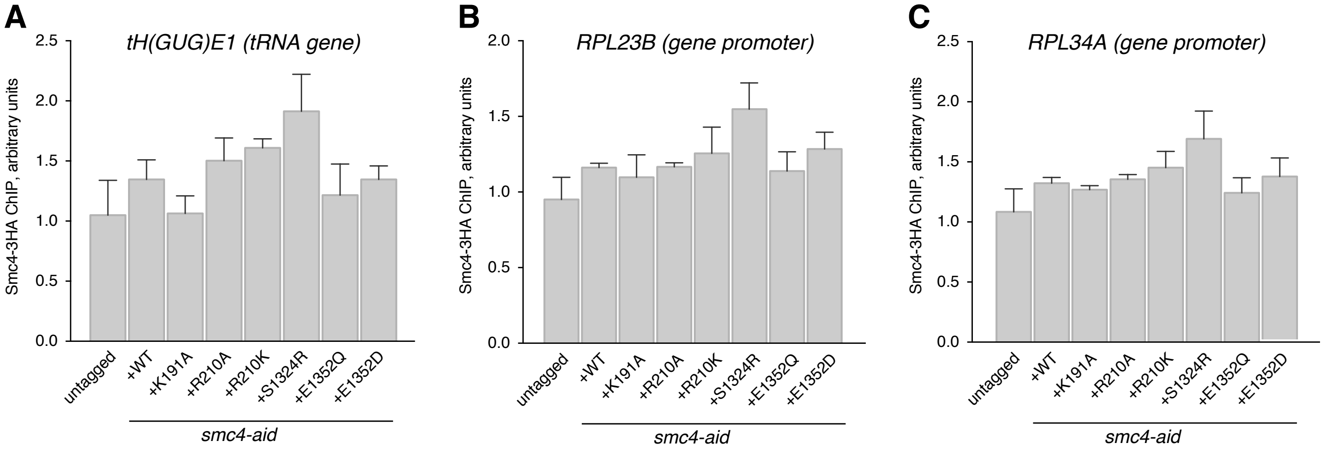

Figure S3. ChIP-qPCR of Chromosome Arm Loci, Related to Figure 1

(A) ChIP-qPCR signal of Smc4-3HA at the *tH(GUG)E1* tRNA gene locus, normalized to a negative binding site, in *smc4-aid* cells in metaphase expressing ectopic wild type or ATPase mutants of Smc4. Error bars represent mean ± s.e.m. (n = 3).

(B) ChIP-qPCR signal of Smc4-3HA at the *RPL23B* ribosomal protein gene promoter, as in (A).

(C) ChIP-qPCR signal of Smc4-3HA at the *RPL34A* ribosomal protein gene promoter, as in (A).

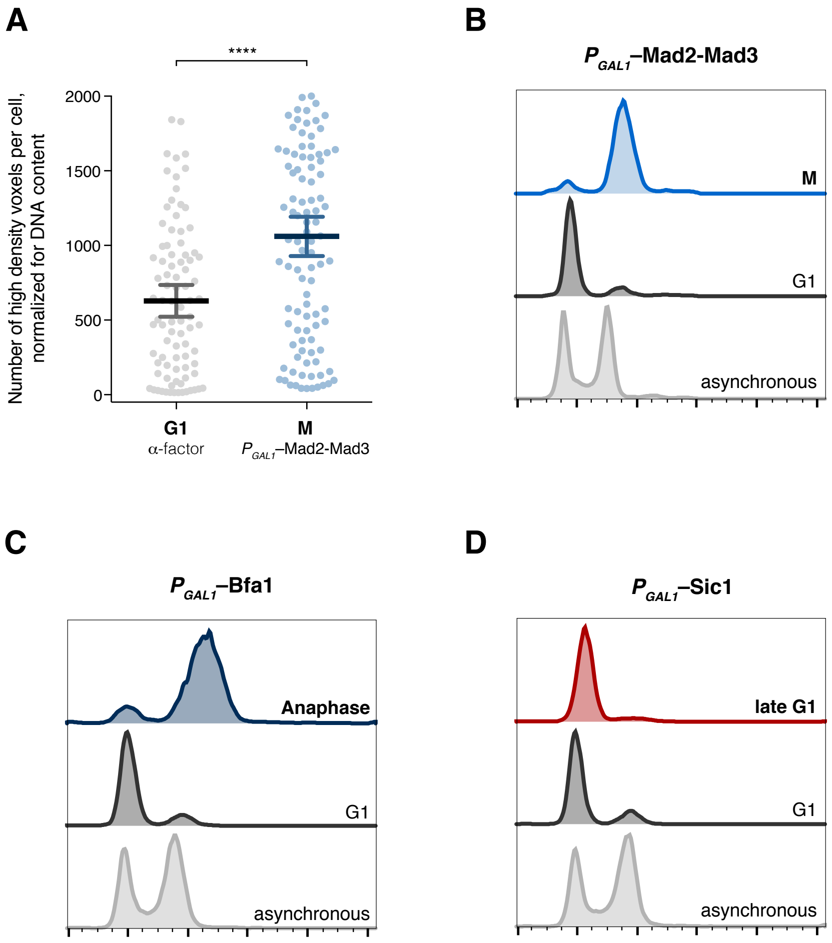

Figure S4. Chromosome Condensation Assay and Illustrative Cell Cycle Arrests, Related to Figure 2

(A) Quantification of the number of high density volumetric pixels (derived as described in the Supplemental Experimental Procedures) in cells arrested in G1 or metaphase (M). The significantly increased number in metaphase illustrates the measurable difference in chromosome compaction between the two cell cycle phases, even when normalized for DNA content. Error bars show the mean and 95% confidence interval ($n \geq 88$, **** $p < 0.0001$; unpaired t-test with equal s.d.).

(B) Overexpression of a Mad2-Mad3 fusion protein under control of the *GAL1* promoter results in a mitotic cell cycle arrest with 2C DNA content.

(C) Overexpression of Bfa1 under control of the *GAL1* promoter results in a late mitotic cell cycle arrest with 2C DNA content.

(D) Overexpression of Sic1 under the control of the *GAL1* promoter results in a cell cycle arrest in late G1 with 1C DNA content.

MSDSPLSKRQKR**KAAQEPELSLDQGDAEEDSQVENRVNLSENTPEPDLPALEASYSKSYTP**R**KLVLSSGENRYAFSQPTN**
STTTSLHVPNLQPPKTSSRGRDHK**SYSQSPPRSPGR**SPTR**RLELLQLSPVK**NSRVELQKIYDSHQSSSKQQSR**LFINELV**
LENFKSYAGK**QVVGPFHTSFSAVVGPNGSGKSNVIDSMLFVFGFR**ANKMRQDR**LSDLIHKSEAFPSLQSCSVAVHFQYVI**
DESSGTSRIDEEKPGLIITRKAFKNNSSKYYINEK**ESSYTEVTKLLKNEGIDLDHKRFLILQGEVENIAQMKPK**AEKESF
DDGLLEYLEDIIGTANYKPLIEERMGQIENLNEVCLEKENREIVDREK**NSLESGKETALEFLEK**EKQLTLLRSKLFQFKL
LQSNSK**LASTLEKISSSNKDLEDERMKFQ**ESLKKVDEIKAQRKEIKDRISSCSSKEK**TLVLER**RELEGTRVSLEERTK**NL**
VSKMEKAEKTLKSTK**HSISEAENMLEELRGQQTEHETEIKDLTQLLEK**ER**SILDDIKLSLK**DK**TKDISAEIIR**HEK**ELEP**
WDLQLQEKESQIQLAESELSLLEETQAKLKK**NVETLEEK**ILAKKTHK**QELQDLILDLKKK**LNSLKDERSQGEKNFTSAHL
KLKEMQKVLNAHRQRAMEARSSLSKAQNKSKVLTALSRLQKSGRINGFHGRLGDLGAIDDSF**DIAISTACPRLDDVVVDT**
VECAQHCIDYLRKNKLGYAR**FILLDR**LR**QFNLQPISTPENVPRLFDLVKPK**NPK**FSNAFYSVLRDTLVAQNLKQANNVAY**
GKKR**FRVVTVDGKLIDISGTMSGGGNHVAKGLMKLGTNQSDKVDDYTPEEVDKIER**ELSERENNFR**VASDTVHEMEEELK**
KLRDHEPDLESQISKAEMEADSLASELTLAEQQVKEAEMAYVKAVSDKAQLNVVMKNLER**LRGEYNDLQSETK**TKKEKIK
GLQDEIMKIGGIKLQMQNSKVESVCQK**LDILVAK**LKKVKSASKKSGGDVVKFQK**LLQNSERDVELSSNELK**VIEEQLKHT
K**LALAENDTNMNETLNLK**VELK**EQSEQLKEQMEDMEESINEFK**SIEIEMKNKLEK**LNSLLTYIKSEITQQEKGLNELSIR**
DVTHTLGMLDDNKMDSVKEDVKNNQELDQEYRSCETQDESEIKDDETSCDNYHPM**NVDETSDEVSR**GIPRLSEDELR**ELD**
VELIESKINELSYYVEETNVDIGVLEEYARRLAEFKRR**KLDLNNAVQKRDEVKEQLGILKKKRFDEFMAGFNIISMTLKE**
MYQMITMGGNAELELVDSLDPFSEGVTFSVMPPKKSWR**NITNLSGGEKTLSSLALVFALHKYKPTPLYVMDEIDAALDFR**
NVSIVANYIKERTK**NAQFIVISLRNNMFELAQQLVGVYK**R**DNRTKSTTIKNIDILNR**T

Figure S5. Mass Spectrometric Identification of Mitotic Smc4 Phosphorylation Sites, Related to Figure 3

Pk epitope-tagged Smc4 was immunoprecipitated from cells synchronized in mitosis by nocodazole treatment. The immunoprecipitate was resolved by SDS-PAGE and the portion of the gel corresponding to Coomassie-stained Smc4 was excised and processed for mass spectrometry. The experiment was repeated and gel slices were digested with trypsin and AspN proteases, respectively. Shown is the combined peptide coverage (bold) of Smc4, encompassing 986 of its 1418 amino acids (70%). Identified phosphorylation sites are highlighted in red. Cdk consensus recognition motifs (SP or TP) are underlined.

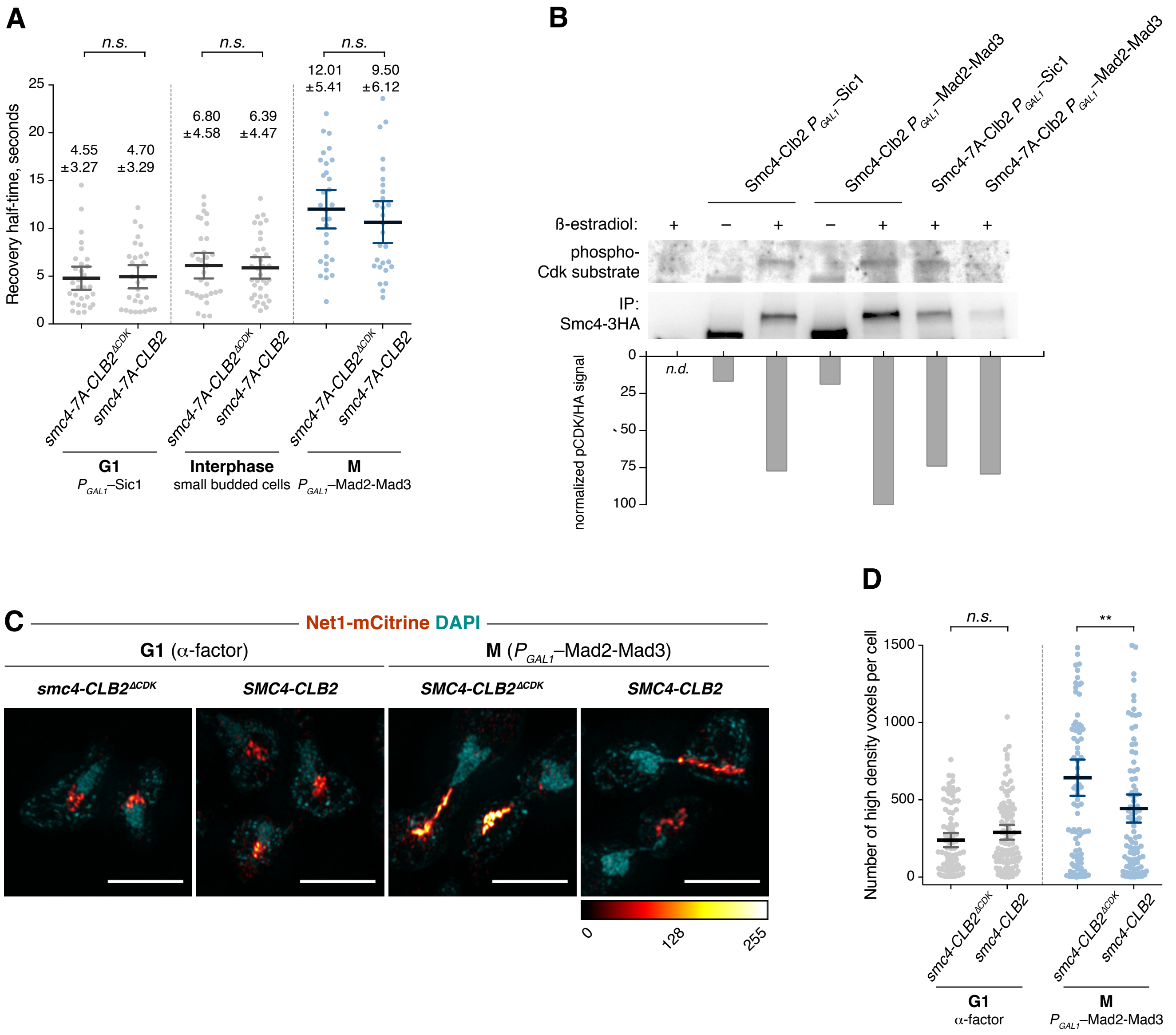

Figure S6. Additional Condensin Dynamics and Chromosome Condensation Assays, Related to Figure 4

(A) Fluorescence recovery half-times, following photobleaching, of the Brn1-3mNeonGreen signal in homozygous diploid *smc4*^{7A}*-CLB2*$^{\Delta CDK}$ and *smc4*^{7A}*-CLB2* cells in G1, interphase (asynchronous small budded cells) and metaphase. Error bars represent mean ± s.d. ($n \geq 30$, n.s. = not significant; ordinary one-way ANOVA, Sidak's multiple comparison test). The absence of statistically significant differences between the two strains indicates that Smc4^{7A} is refractory to Cdk regulation.

(B) Phosphorylation of Smc4-Clb2 fusion, under the indicated conditions and strain backgrounds, assessed by immunoprecipitation by means of the HA tag, followed by Western blotting with an anti phospho–Cdk substrate antibody. ß-estradiol was used to produce the Cre-recombinase mediated Smc4-Clb2 fusion.

(C) Examples of rDNA morphology and Net1-mCitrine intensities during the experiment described in (D).

(D) Chromosome condensation of *smc4-CLB2*$^{\Delta CDK}$ and *smc4-CLB2* strains in a G1 arrest produced with α-factor or a metaphase arrest due to overexpression of Mad2-Mad3. Shown are the means with 95% confidence intervals ($n \geq 86$, ** $p < 0.01$; ordinary one-way ANOVA, Sidak's multiple comparison test). There is no significant difference between the two strains in G1, but Smc4-Clb2 causes impaired chromosome condensation in metaphase.

SUPPLEMENTAL EXPERIMENTAL PROCEDURES

Resource Table

Reagent or Resource	Source	Identifier
**Antibodies**		
anti-HA, mouse	in-house	clone 12CA5
anti-HA, rat	Roche	clone 3F10
anti-HA, mouse	Santa Cruz Biotech	clone F-7, cat. sc-7392
anti-Pk, mouse	Bio-Rad/AbD Serotec	clone SV5-Pk1
anti-myc, mouse	in-house	clone 9E10
anti-tubulin, mouse	in-house	clone TAT-1
anti-Smc2 polyclonal serum	A. Strunnikov	
anti-phosphoCDK	Cell Signaling	cat. #9477
Atto 594 coupled GFP booster nanobody	Chromotek	cat. gba594
AlexaFluor 594 goat anti-rat secondary	Molecular Probes	cat. A-11007
**Chemicals and Peptides**		
α-factor	in-house	
DAPI	Sigma-Aldrich	D9542
Nocodazole	Sigma-Aldrich	M1404
Trypsin Gold, Mass Spectrometry Grade	Promega	V5280
Indole acetic acid (IAA)	Sigma-Aldrich	I3750
**Critical Commercial Assays**		
–		
**Experimental Models: Organisms/Strains**		
A list of the budding yeast strains used in this study, together with their genotypes, can be found in Table S1.		
**Software and Algorithms**		
ImageJ/Fiji	Open-source	http://fiji.sc
Zen (Black edition)	Zeiss	https://www.zeiss.com
Prism	Prism	https://www.graphpad.com

Yeast Strains and Techniques

Genes were tagged at their endogenous loci (*NET1, BRN1, SMC2 and SMC4*) by gene targeting using polymerase chain reaction (PCR) products (Knop et al., 1999; Sheff and Thorn, 2004). All vectors were constructed using sequence-independent cloning using InFusion HD reagents (Clontech/Takara Bio). *SMC2* and *SMC4*, along with their promoters and a 3HA epitope tag, were amplified from a wild type *W303* strain and cloned into the yeast integration vectors YIplac211 and YIplac204 respectively (Gietz and Sugino, 1988). ATPase mutants were constructed from the wild type vectors using the Q5 site-directed mutagenesis kit (New England Biolabs) following the manufacturer's instructions. Overexpression of Sic1, Mad2-Mad3 and Bfa1 was achieved by cloning the genes under the control of the *GAL1* promoter into YIplac128. Unless otherwise stated, yeast cultures were grown in rich YP medium supplemented with 2% glucose, with 2% raffinose, or with 2% raffinose + 2% galactose (Rose et al., 1990). Cell synchronization using α-factor block and release was performed as described (O'Reilly et al., 2012). For depletion of endogenous condensin SMC subunits, we used C-terminal mini auxin-induced degron tags with an additional epitope tag to aid detection (3Pk-miniAID; Kubota et al., 2013), and treated the cultures with 1 mM indole-3-acetic acid. For induction of Smc4-Clb2 fusions, we activated Cre recombinase fused to an estrogen binding domain EBD78 as previously described (Kuilman et al., 2014) by addition 1 μM β-estradiol to the growth medium overnight.

Western Blotting and Immunoprecipitation

Protein extracts for western blotting were prepared following cell fixation using trichloroacetic acid, as previously described (Foiani et al., 1994), and analyzed by SDS-polyacrylamide gel electrophoresis. Antibodies used for detection are listed in the Resource Table. Chemiluminescent detection was performed using ECL Prime reagents

(GE Life Sciences); membranes were scanned as a time series using an ImageQuant LAS4000 imager in the linear detection range, and unsaturated images were selected for quantification.

For immunoprecipitation of Protein A-tagged condensin complexes, 3×10^8 cells were harvested, washed with distilled water and resuspended in 400 µl extraction buffer (50 mM HEPES/KOH pH 8.0, 0.1 M KCl, 2.5 mM $MgCl_2$, 10 % glycerol, 0.1 % SDS, 0.25 % Triton, 1 mM DTT, 1 mM PMSF, protease inhibitors). Cells were broken with glass beads, clarified by centrifugation and pre-cleared by incubation with protein A sepharose beads (GE Healthcare) at 4 °C for 1-2 hours. Protein A fusion proteins were immunoprecipitated from the pre-cleared extracts using IgG sepharose beads (GE Healthcare) at 4 °C for 1-2 hours. The beads were washed extensively with extraction buffer and the proteins eluted either with elution buffer (50 mM Tris-HCl pH 8.0, 10 mM EDTA, 1 % SDS) at 65 °C or by directly boiling the beads in SDS sample buffer. Elutions were analyzed for their protein content by SDS-polyacrylamide gel electrophoresis and immunoblotting.

For immunoprecipitation of HA-tagged Smc4-Clb2 fusion proteins, 3×10^8 cells were harvested, washed with distilled water and resuspended in 400 µl cold extraction buffer (50 mM HEPES/KOH pH 8.0, 0.1 M KCl, 2.5 mM $MgCl_2$, 10 % glycerol, 0.1 % SDS, 0.25 % Triton, 1 mM DTT, 1 mM PMSF, protease inhibitors). Cells were broken with glass beads, clarified by centrifugation and pre-cleared by incubation with Protein G magnetic beads (GE Healthcare) at 4 °C for 30-60 mins. Pre-cleared extracts were incubated with ~3 µg of anti-HA antibody (clone 12CA5) at 4 °C overnight. HA-tagged proteins were then immunoprecipitated with Protein G magnetic beads at 4 °C for 1 hour. The beads were washed extensively, and proteins were eluted by boiling in SDS sample buffer and analyzed by SDS-PAGE and immunoblotting. Due to low signal and high background, blots probed with phospho-CDK substrate antibodies were exposed to chemiluminescent film overnight, scanned and subjected to 100-pixel rolling ball background subtraction in ImageJ/Fiji prior to quantification.

Flow Cytometry

For preparation of samples for flow cytometry, cells from 1 ml of culture ($OD_{600} > 0.15$) were spun down, fixed in 1 ml ice-cold 70% ethanol, and incubated at 4 °C overnight. Cells were pelleted and resuspended in 1 ml of 50 mM Tris-HCl, pH 7.5 containing 0.1 mg/ml RNase A (1:100 from 10 mg/ml stock, DNase free after boiling). The cells were incubated at 37 °C for 2–16 hours, pelleted and resuspended in 0.4 ml of FACS buffer (200 mM Tris-HCl, pH 7.5; 211 mM NaCl; 78 mM $MgCl_2$) containing 50 µg/ml propidium iodide (1:20 from 1 mg/ml propidium iodide stock in water). Samples were sonicated for 5 seconds and stored in the dark at 4 °C until analysis. For analysis, 50–100 µl of stained cells were diluted in 600 µl of 50 mM Tris-HCl, pH 7.5. Immediately after dilution, 10000 events per sample were counted on a Beckton-Dickinson FACScan with settings in linear mode (FSC threshold: 52; FSC: detector: E01, amplifier: 1.4; SSC: detector: 400, amplifier: 1; FL1, FL3 off; FL2: detector: 750, amplifier: 7). The resulting cell counts were analyzed using FlowJo.

Chromosome Spreads

Cells from 2 ml of culture were harvested, resuspended in 1 ml S1 (100 mM potassium phosphate buffer, pH 7.4; 0.5 mM $MgCl_2$; 1.2 M sorbitol), and kept on ice until all samples from the course of the experiment were harvested. For spheroplastation, cells were resuspended in 200 µl S2 (S1 + per ml, 20 µl 1 M DTT and 14 µl 10 mg/ml zymolase T-100). The suspension was incubated in a 37 °C water bath for 20 min. 1 ml of ice-cold S3 (0.1 M 2-(N-morpholino)ethanesulfonic acid; 1 mM EDTA; 0.5 mM $MgCl_2$; 1 M sorbitol, pH 6.4) was added. Spheroplasts were washed and resuspended in 200 µl S3 and kept on ice until spreading. We used acid-washed 12 well multitest slides (MP Biomedicals, #6041205E) for spreading. The following were pipetted in rapid succession onto each well used: 1 µl cell suspension; 2 µl fixative (4% paraformaldehyde, dissolved in water at 60–80 °C; 3.4% sucrose; per ml, 1 µl 0.2 M NaOH); 2 µl spheroplast suspension; 4 µl 1% lipsol in water; 4 µl fixative. The drop was gently distributed onto the well using a pipette tip, avoiding contact with the glass. Slides were dried overnight at room temperature in a fume hood. For immunostaining, slides were placed in phosphate-buffered saline (PBS) for 10 min at room temperature. 50 µl of blocking buffer (1% BSA in PBS) was pipetted onto each well, and placed in a humid chamber for 1 hour at 25 °C. The blocking buffer was aspirated and 50 µl of primary antibody in blocking buffer was added to each well (e.g. rat anti-HA 3F10, 1:500), and incubated in a humid chamber for 2 hours at 25 °C. The wells were rinsed 3 times with PBS before adding 50 µl secondary antibody diluted in blocking buffer (e.g. AlexaFluor 594 anti-rat, 1:1000) to each well. The slide was incubated in a darkened humid chamber for 2 hours at 25 °C. Wells were again rinsed three times with PBS and then three times with water. DNA was then stained with 50 µl of a 100 µg/ml solution of DAPI in water pipetted onto each well, and incubated in the darkened humid chamber, 30 min, 25 °C. Wells were rinsed 3 times with water and aspirated. 5 µl of antifade (SlowFade Diamond, Life Technologies)

was pipetted onto each well. An acid washed coverslip (24 x 60 mm high precision, no. 1.5H, CellPath Ltd, SAN-2460-03A) was lowered onto the antifade mounting medium, and sealed with Valap (equal parts vaseline, lanolin & paraffin). The slide was stored in the dark at –20 °C until imaging.

For quantification of Smc staining intensity, images were typically acquired as detailed in 'Structured Illumination Microscopy' below. We created sum-intensity projections of the acquired image stacks, and used circles of 4.5 μm diameter drawn with the aid of DAPI-stained masses in the 405 nm channel, to measure the absolute staining intensity in the 592 nm channel. To exclude effects of variable ectopic protein expression, the absolute staining intensities were normalized to cellular expression levels of the HA-tagged Smc proteins as determined by loading-controlled Western blots in the same experiment. To minimize the effect of noise from dust and cellular debris, we excluded the extreme 5th percentile at either end from the analysis.

ChIP-qPCR

Cells from the indicated strains growing in YPD were arrested in G1 by adding α-factor. After 2 hours, cells were released from the G1 block into YPD containing 88 μg/ml IAA and 5 μg/ml nocodazole. 2 hours after release, when the cultures were uniformly arrested in mitosis, cells were fixed with formaldehyde and harvested. Protein extracts were prepared and disrupted by sonication. The DNA fragments cross-linked to HA-tagged Smc4 were enriched by immuno-precipitation with anti HA-probe F7 antibody (Santa Cruz Biotechnology). After reversal of the cross-links, DNA both from immunoprecipitates and from total cell extracts was cleaned up and quantified using PowerUP SYBR® Green Master Mix (Applied Biosystems, Life Technologies) and a Quant Studio 12 Real-Time PCR System (Thermo Fisher Scientific). The following primers were used (CEN9 – centromere 9, tH(GUG)E1 – tRNA gene, RPL23B – ribosomal protein gene promoter, RPL34A – ribosomal protein gene promoter, N3 (CIN8) – negative binding site for normalization):

CEN9 F	5' TGTCACCTGGCTGTTTTGAG
CEN9 R	5' TGGGTAATGTCAGCTGTGGA
tH(GUG)E1 F	5' GAAACCCTGGTTCGATTCTAGGAG
tH(GUG)E1 R	5' GCTCTCATGATCACCACATCTGAC
RPL23B F	5' CCGTCAAGCTAAGTCTTGGAGAAG
RPL23B R	5' CCTTAGGATTAGCGATGACACCAG
RPL34A F	5' GTCTTGTGGGTCTTGGAAACAG
RPL34A R	5' CAAAGTGTGGTGACTGTGGTAG
N3 (CIN8) F	5' AGGGCACAACTAGATAAACAGCA
N3 (CIN8) R	5' GGGCCATTTGCATTACCTCAGTCA

Cell Fixation and Nanobody Staining

Cells were fixed and stained using an adapted published method (Ries et al., 2012). Briefly, we fixed cells by addition of 3.6% formaldehyde directly to the liquid culture (1:10 by volume from 36% stock solution of EM grade formaldehyde, TAAB, #F003). After incubation on a roller for 15 min at room temperature, the cells were washed once with TBS (150 mM NaCl, 50 mM Tris-Cl pH 8.0) containing 50 mM NH_4Cl and then 3x with TBS. Cells were permeabilized in blocking buffer (TBS + 5% BSA, 0.25% TritonX-100, 0.005% NaN_3) for 1 hour at room temperature. Nanobody staining was then performed at 4 °C overnight in staining buffer (TBS + 1% BSA, 0.05% TritonX-100, 0.001% NaN_3) with 1/100 Atto 594–conjugated GFP booster nanobody (Chromotek, gba594). Cells were then washed 3x in TBS, stained with 0.1 μg/ml DAPI in TBS, washed 3x, mounted on acid-washed high precision (170 μm ± 5 μm thick) coverslips, and sealed with Valap.

Structured Illumination Microscopy

Structured illumination microscopy was performed on an API OMX v3 microscope with a 100x objective and a 'live' filter set: DAPI – excitation 405 nm, emission 465-500 nm; YFP – excitation 514 nm, emission 525-575 nm; mCherry/Alexa 594 – excitation 592.5 nm, emission 602-656 nm. We typically acquired images of 512 x 512 pixels at 1 x 1 binning and, per channel, 33 slices at 0.125 μm spacing for a total sample thickness of 4 μm. For images not acquired in structured illumination mode (YFP – 60 ms exposure, 1%T), deconvolution was applied in softWoRx using the conservative ratio method with medium noise filtering and an experimentally determined point spread function. For images acquired in structured illumination mode (DAPI – 60 ms exposure, 50%T and Alexa 594 – 100

ms exposure, 33.3%T), default reconstruction parameters were used. For multichannel images, registration parameters for aligning the different channels were determined empirically using a 10 nm–spacing grid slide and Tetraspeck beads.

Chromosome Condensation Assay

In order to extract the high frequency sub-diffraction information provided by SIM over conventional imaging, we adapted a published method (Marbouty et al., 2015) that applied a 3D Butterworth bandpass filter with spatial cutoffs at 100 nm and 400 nm. Using ImageJ, we applied an analogous Fourier bandpass frequency filter with cutoffs at 3 and 10 pixels (5% tolerance with no stripe suppression), which corresponded to structures between 118.5 and 395 pixels (39.5 nm/pixel). We then automatically selected the brightest pixels in each cell volume by using ImageJ MaxEntropy thresholding with a stack histogram for each cell (Kapur et al., 1985). Finally, we counted the number of outlined pixels per slice (voxels); their sum served as a high resolution measure of chromosome condensation. Since SIM reconstructions of interphase cells were noisier due to lower signal intensity, we excluded the extreme 5th percentile of values in experiments that included G1 cells.

Table S1. Yeast Strains Used in this Study

Strain	Genotype	Background	Source	Fig
Y4267	*MAT*a *ade2-1::OsTIR1-9myc:ADE2*	W303	Lab collection	S1A, S1B, S6B
Y4279/ RT157	*MAT*a *ade2-1::OsTIR1-9myc:ADE2 SMC2-3HA:URA3*	W303	This study	1A-B, S1A, S2A-C
Y4276/ RT146	*MAT*a *ade2-1::OsTIR1-9myc:ADE2 SMC2-3Pk-miniAID:kanR*	W303	This study	1A-B, S1A, S2A-C
Y4973/ RT427	*MAT*a *ade2-1::OsTIR1-9myc:ADE2 SMC2-3Pk-miniAID:kanR ura3-1::*P_{SMC2}*–SMC2*^{WT}*-3HA:URA3*	W303	This study	1A-B, S1A, S2A-C
Y4974/ RT429	*MAT*a *ade2-1::OsTIR1-9myc:ADE2 SMC2-3Pk-miniAID:kanR ura3-1::*P_{SMC2}*–SMC2*^{K38A}*-3HA:URA3*	W303	This study	1A-B, S1A, S2A-C
Y4975/ RT431	*MAT*a *ade2-1::OsTIR1-9myc:ADE2 SMC2-3Pk-miniAID:kanR ura3-1::*P_{SMC2}*–SMC2*^{R58A}*-3HA:URA3*	W303	This study	1A-B, S1A, S2A-C
Y4976/ RT433	*MAT*a *ade2-1::OsTIR1-9myc:ADE2 SMC2-3Pk-miniAID:kanR ura3-1::*P_{SMC2}*–SMC2*^{R58K}*-3HA:URA3*	W303	This study	1A-B, S1A, S2A-C
Y4977/ RT435	*MAT*a *ade2-1::OsTIR1-9myc:ADE2 SMC2-3Pk-miniAID:kanR ura3-1::*P_{SMC2}*–SMC2*^{S1085R}*-3HA:URA3*	W303	This study	1A-B, S1A, S2A-C
Y4978 RT437	*MAT*a *ade2-1::OsTIR1-9myc:ADE2 SMC2-3Pk-miniAID:kanR ura3-1::*P_{SMC2}*–SMC2*^{E1113Q}*-3HA:URA3*	W303	This study	1A-B, S1A, S2A-C
Y4979/ RT439	*MAT*a *ade2-1::OsTIR1-9myc:ADE2 SMC2-3Pk-miniAID:kanR ura3-1::*P_{SMC2}*–SMC2*^{E1113D}*-3HA:URA3*	W303	This study	1A-B, S1A, S2A-C
Y4280/ RT159	*MAT*a *ade2-1::OsTIR1-9myc:ADE2 SMC4-3HA:TRP1*	W303	This study	1C-D, S1B, S2D-F
Y4278/ RT149	*MAT*a *ade2-1::OsTIR1-9myc:ADE2 SMC4-3Pk-miniAID:kanR*	W303	This study	1C-E, S1B, S2D-F
Y4966/ RT242	*MAT*a *ade2-1::OsTIR1-9myc:ADE2 SMC4-3Pk-miniAID:kanR trp1-1::*P_{SMC4}*–SMC4*^{WT}*-3HA:TRP1*	W303	This study	1C-E, S1B, S2D-F
Y4967/ RT244	*MAT*a *ade2-1::OsTIR1-9myc:ADE2 SMC4-3Pk-miniAID:kanR trp1-1::*P_{SMC4}*–SMC4*^{K191A}*-3HA:TRP1*	W303	This study	1C-E, S1B, S2D-F
Y4968/ RT246	*MAT*a *ade2-1::OsTIR1-9myc:ADE2 SMC4-3Pk-miniAID:kanR trp1-1::*P_{SMC4}*–SMC4*^{R210A}*-3HA:TRP1*	W303	This study	1C-E, S1B, S2D-F
Y4969/ RT248	*MAT*a *ade2-1::OsTIR1-9myc:ADE2 SMC4-3Pk-miniAID:kanR trp1-1::*P_{SMC4}*–SMC4*^{R210K}*-3HA:TRP1*	W303	This study	1C-E, S1B, S2D-F
Y4970/ RT250	*MAT*a *ade2-1::OsTIR1-9myc:ADE2 SMC4-3Pk-miniAID:kanR trp1-1::*P_{SMC4}*–SMC4*^{S1324R}*-3HA:TRP1*	W303	This study	1C-E, S1B, S2D-F
Y4971/ RT252	*MAT*a *ade2-1::OsTIR1-9myc:ADE2 SMC4-3Pk-miniAID:kanR trp1-1::*P_{SMC4}*–SMC4*^{E1352Q}*-3HA:TRP1*	W303	This study	1C-E, S1B, S2D-F
Y4972/ RT254	*MAT*a *ade2-1::OsTIR1-9myc:ADE2 SMC4-3Pk-miniAID:kanR trp1-1::*P_{SMC4}*–SMC4*^{E1352D}*-3HA:TRP1*	W303	This study	1C-E, S1B, S2D-F
Y3654	*MAT*a *BRN1-18myc::TRP1 SMC2-2ProtA:SpHIS5*	S288C	This study	1F
Y3733	*MAT*a *BRN1-18myc:TRP1*	S288C	This study	1F-G
Y3600	*MAT*a *smc2-8 BRN1-18myc::LEU2 pRS306-SMC2-6His-HA-2ProtA:URA3*	S288C	This study	1F
Y3736	*MAT*a *smc2-8 BRN1-18myc:LEU2 pRS306-smc2K38A-6His-HA-2ProtA:URA3*	S288C	This study	1F
Y3735	*MAT*a *smc2-8 BRN1-18myc:LEU2 pRS306-smc2R58A-6His-HA-2ProtA:URA3*	S288C	This study	1F
Y3657	*MAT*a *smc2-8 BRN1-18myc:LEU2 pRS306-smc2S1085R-6His-HA-2ProtA:URA3*	S288C	This study	1F
Y3656	*MAT*a *smc2-8 BRN1-18myc:LEU2 pRS306-smc2E1131Q-6His-HA-2ProtA:URA3*	S288C	This study	1F
Y3652	*MAT*a *BRN1-18myc::KlTrp1 SMC4-2protA::SpHIS5*	S288C	This study	1G
Y3601	*MAT*a *smc4-1 BRN1-18myc::SpHIS5 pRS306-SMC4-6His-HA-2ProtA:URA3*	S288C	This study	1G
Y3738	*MAT*a *smc4-1 BRN1-18myc::SpHIS5 pRS306-smcK191A-6His-HA-2ProtA:URA3*	S288C	This study	1G
Y3739	*MAT*a *smc4-1 BRN1-18myc::SpHIS5 pRS306-smc4R210A-6His-HA-2ProtA:URA3*	S288C	This study	1G

Y3644	MATa smc4-1 BRN1-18myc::SpHIS5 pRS306-smc4S1324R-6His-HA-2ProtA:URA3	S288C	This study	1G
Y3658	MATa smc4-1 BRN1-18myc::SpHIS5 pRS306-smc4E1352Q-6His-HA-2ProtA:URA3	S288C	This study	1G
Y4998/ RT346a	MATa ade2-1::OsTIR1-9myc:ADE2 SMC4-3HA:TRP1 NET1-yEmCitrine:HIS3 leu2-2,112::P_{GAL1}–MAD2-MAD3:LEU2	W303	This study	2A-B, S4A-B, 3D-E
Y4997/ RT315	MATa ade2-1::OsTIR1-9myc:ADE2 SMC4-3Pk-miniAID:kanR NET1-yEmCitrine:HIS3 leu2-2,112::P_{GAL1}–MAD2-MAD3:LEU2	W303	This study	2A-B
Y4980/ RT401	MATa ade2-1::OsTIR1-9myc:ADE2 SMC4-3Pk-miniAID:kanR trp1-1::P_{SMC4}–$SMC4^{WT}$-3HA:TRP1 NET1-yEmCitrine:HIS3 leu2-2,112::P_{GAL1}–MAD2-MAD3:LEU2	W303	This study	2A-B
Y4981/ RT402	MATa ade2-1::OsTIR1-9myc:ADE2 SMC4-3Pk-miniAID:kanR trp1-1::P_{SMC4}–$SMC4^{K191A}$-3HA:TRP1 NET1-yEmCitrine:HIS3 leu2-2,112::P_{GAL1}–MAD2-MAD3:LEU2	W303	This study	2A-B
Y4982/ RT403	MATa ade2-1::OsTIR1-9myc:ADE2 SMC4-3Pk-miniAID:kanR trp1-1::P_{SMC4}–$SMC4^{R210A}$-3HA:TRP1 NET1-yEmCitrine:HIS3 leu2-2,112::P_{GAL1}–MAD2-MAD3:LEU2	W303	This study	2A-B
Y4983/ RT404	MATa ade2-1::OsTIR1-9myc:ADE2 SMC4-3Pk-miniAID:kanR trp1-1::P_{SMC4}–$SMC4^{R210K}$-3HA:TRP1 NET1-yEmCitrine:HIS3 leu2-2,112::P_{GAL1}–MAD2-MAD3:LEU2	W303	This study	2A-B
Y4984/ RT405	MATa ade2-1::OsTIR1-9myc:ADE2 SMC4-3Pk-miniAID:kanR trp1-1::P_{SMC4}–$SMC4^{S1324R}$-3HA:TRP1 NET1-yEmCitrine:HIS3 leu2-2,112::P_{GAL1}–MAD2-MAD3:LEU2	W303	This study	2A-B
Y4985/ RT406a	MATa ade2-1::OsTIR1-9myc:ADE2 SMC4-3Pk-miniAID:kanR trp1-1::P_{SMC4}–$SMC4^{E1352Q}$-3HA:TRP1 NET1-yEmCitrine:HIS3 leu2-2,112::P_{GAL1}–MAD2-MAD3:LEU2	W303	This study	2A-B
Y4986/ RT407a	MATa ade2-1::OsTIR1-9myc:ADE2 SMC4-3Pk-miniAID:kanR trp1-1::P_{SMC4}–$SMC4^{E1352D}$-3HA:TRP1 NET1-yEmCitrine:HIS3 leu2-2,112::P_{GAL1}–MAD2-MAD3:LEU2	W303	This study	2A-B
Y5001/ RT347	MATa ade2-1::OsTIR1-9myc:ADE2 SMC4-3HA:TRP1 NET1-yEmCitrine:HIS3 leu2-2,112::P_{GAL1}–BFA1:LEU2	W303	This study	2C-D, S4C
Y5000/ RT276	MATa ade2-1::OsTIR1-9myc:ADE2 SMC4-3Pk-miniAID:kanR NET1-yEmCitrine:HIS3 leu2-2,112::P_{GAL1}–BFA1:LEU2	W303	This study	2C-D
Y4987/ RT408	MATa ade2-1::OsTIR1-9myc:ADE2 SMC4-3Pk-miniAID:kanR trp1-1::P_{SMC4}–$SMC4^{WT}$-3HA:TRP1 NET1-yEmCitrine:HIS3 leu2-2,112::P_{GAL1}–BFA1:LEU2	W303	This study	2C-D
Y4988/ RT409	MATa ade2-1::OsTIR1-9myc:ADE2 SMC4-3Pk-miniAID:kanR trp1-1::P_{SMC4}–$SMC4^{K191A}$-3HA:TRP1 NET1-yEmCitrine:HIS3 leu2-2,112::P_{GAL1}–BFA1:LEU2	W303	This study	2C-D
Y4989/ RT410	MATa ade2-1::OsTIR1-9myc:ADE2 SMC4-3Pk-miniAID:kanR trp1-1::P_{SMC4}–$SMC4^{R210A}$-3HA:TRP1 NET1-yEmCitrine:HIS3 leu2-2,112::P_{GAL1}–BFA1:LEU2	W303	This study	2C-D
Y4990/ RT411	MATa ade2-1::OsTIR1-9myc:ADE2 SMC4-3Pk-miniAID:kanR trp1-1::P_{SMC4}–$SMC4^{R210K}$-3HA:TRP1 NET1-yEmCitrine:HIS3 leu2-2,112::P_{GAL1}–BFA1:LEU2	W303	This study	2C-D
Y4991/ RT412	MATa ade2-1::OsTIR1-9myc:ADE2 SMC4-3Pk-miniAID:kanR trp1-1::P_{SMC4}–$SMC4^{S1324R}$-3HA:TRP1 NET1-yEmCitrine:HIS3 leu2-2,112::P_{GAL1}–BFA1:LEU2	W303	This study	2C-D
Y4992/ RT413a	MATa ade2-1::OsTIR1-9myc:ADE2 SMC4-3Pk-miniAID:kanR trp1-1::P_{SMC4}–$SMC4^{E1352Q}$-3HA:TRP1 NET1-yEmCitrine:HIS3 leu2-2,112::P_{GAL1}–BFA1:LEU2	W303	This study	2C-D
Y4993/ RT414a	MATa ade2-1::OsTIR1-9myc:ADE2 SMC4-3Pk-miniAID:kanR trp1-1::P_{SMC4}–$SMC4^{E1352D}$-3HA:TRP1 NET1-yEmCitrine:HIS3 leu2-2,112::P_{GAL1}–BFA1:LEU2	W303	This study	2C-D
Y5004/ RT379a	MATa/MATα ade2-1/ade2-1::OsTIR1-9myc:ADE2 BRN1-3mNeonGreen:HIS3/BRN1-3mNeonGreen:NAT trp1-1/trp1-1::P_{GAL1}–$SIC1^{V5.V33.A76}$-HA:TRP1	W303	This study	2E, 3F
Y5005/ RT380a	MATa/MATα ade2-1/ade2-1::OsTIR1-9myc:ADE2 BRN1-3mNeonGreen:HIS3/BRN1-3mNeonGreen:NAT leu2-2,112/leu2-2,112::P_{GAL1}–MAD2-MAD3:LEU2	W303	This study	2E-F, 3F

Y5007/RT394	*MAT*a/*MAT*α *ade2-1/ade2-1::OsTIR1-9myc:ADE2 BRN1-3mNeonGreen:HIS3/BRN1-3mNeonGreen:NAT SMC4*^{R210A}*::TRP/SMC4*^{R210A}*::TRP ura3-1/ura3-1::P*$_{GAL1}$*–SIC1*$^{V5.V33.A76}$*-HA:URA3*	W303	This study	2E
Y5008/RT395	*MAT*a/*MAT*α *ade2-1/ade2-1::OsTIR1-9myc:ADE2 BRN1-3mNeonGreen:HIS3/BRN1-3mNeonGreen:NAT SMC4*^{R210A}*::TRP/SMC4*^{R210A}*::TRP leu2-2,112/leu2-2,112::P*$_{GAL1}$*–MAD2-MAD3:LEU2*	W303	This study	2E-F
Y4426	*SMC4-9Pk:URA3*	W303	This study	3B-C
Y4999/RT351	*MAT*a *ade2-1::OsTIR1-9myc:ADE2 SMC4*^{7A}*-3HA:URA3:TRP1 NET1-yEmCitrine:HIS3 leu2-2,112::P*$_{GAL1}$*–MAD2-MAD3:LEU2*	W303	This study	3D-E
Y5010/RT399	*MAT*a/*MAT*α *ade2-1/ade2-1::OsTIR1-9myc:ADE2 BRN1-3mNeonGreen:HIS3/BRN1-3mNeonGreen:NAT SMC4*^{7A}*:URA3/SMC4*^{7A}*:URA3 trp1-1/trp1-1::P*$_{GAL1}$*–SIC1*$^{V5.V33.A76}$*-HA:TRP1*	W303	This study	3F
Y5011/RT400	*MAT*a/*MAT*α *ade2-1/ade2-1::OsTIR1-9myc:ADE2 BRN1-3mNeonGreen:HIS3/BRN1-3mNeonGreen:NAT SMC4*^{7A}*:URA3/SMC4*^{7A}*:URA3 leu2-2,112/leu2-2,112::P*$_{GAL1}$*–MAD2-MAD3:LEU2*	W303	This study	3F
Y5016/RT441	*MAT*a/*MAT*α *ade2-1/ade2-1::OsTIR1-9myc:ADE2 BRN1-3mNeonGreen::HIS3/BRN1-3mNeonGreen::NAT SMC4-CLB2-3HA:kanR/SMC4-CLB2-3HA:TRP1 leu2-2,112/leu2-2,112::P*$_{GAL1}$*–SIC1*$^{V5.V33.A76}$*–Cre-EBD78*	W303	This study	4B, S6B
Y5017/RT443	*MAT*a/*MAT*α *ade2-1/ade2-1::OsTIR1-9myc:ADE2 BRN1-3mNeonGreen::HIS3/BRN1-3mNeonGreen::NAT SMC4-CLB2-3HA:kanR/SMC4-CLB2-3HA:TRP1 leu2-2,112/leu2-2,112::P*$_{GAL1}$*–MAD2-MAD3–Cre-EBD78*	W303	This study	4B, S6B
Y5018/RT445	*MAT*a/*MAT*α *ade2-1/ade2-1::OsTIR1-9myc:ADE2 BRN1-3mNeonGreen::HIS3/BRN1-3mNeonGreen::NAT SMC4-CLB2*$^{\Delta CDK}$*-3HA:kanR/SMC4-CLB2*$^{\Delta CDK}$*-3HA:TRP1 leu2-2,112/leu2-2,112::P*$_{GAL1}$*–SIC1*$^{V5.V33.A76}$*–Cre-EBD78*	W303	This study	4B
Y5019/RT447	*MAT*a/*MAT*α *ade2-1/ade2-1::OsTIR1-9myc:ADE2 BRN1-3mNeonGreen::HIS3/BRN1-3mNeonGreen::NAT SMC4-CLB2*$^{\Delta CDK}$*-3HA:kanR/SMC4-CLB2*$^{\Delta CDK}$*-3HA:TRP1 leu2-2,112/leu2-2,112::P*$_{GAL1}$*–MAD2-MAD3–Cre-EBD78*	W303	This study	4B
Y5020/RT449	*MAT*a/*MAT*α *ade2-1/ade2-1::OsTIR1-9myc:ADE2 BRN1-3mNeonGreen::HIS3/BRN1-3mNeonGreen::NAT SMC4*^{7A}*-CLB2-3HA:kanR:URA3/SMC4*^{7A}*-CLB2-3HA:TRP1:URA3 leu2-2,112/leu2-2,112::P*$_{GAL1}$*–SIC1*$^{V5.V33.A76}$*–Cre-EBD78*	W303	This study	S6A, S6B
Y5021/RT451	*MAT*a/*MAT*α *ade2-1/ade2-1::OsTIR1-9myc:ADE2 BRN1-3mNeonGreen::HIS3/BRN1-3mNeonGreen::NAT SMC4*^{7A}*-CLB2-3HA:kanR:URA3/SMC4*^{7A}*-CLB2-3HA:TRP1:URA3 leu2-2,112/leu2-2,112::P*$_{GAL1}$*–MAD2-MAD3–Cre-EBD78*	W303	This study	S6A, S6B
Y5022/RT453	*MAT*a/*MAT*α *ade2-1/ade2-1::OsTIR1-9myc:ADE2 BRN1-3mNeonGreen::HIS3/BRN1-3mNeonGreen::NAT SMC4*^{7A}*-CLB2*$^{\Delta CDK}$*-3HA:kanR:URA3/SMC4*^{7A}*-CLB2*$^{\Delta CDK}$*-3HA:TRP1:URA3 leu2-2,112/leu2-2,112::P*$_{GAL1}$*–SIC1*$^{V5.V33.A76}$*–Cre-EBD78*	W303	This study	S6A
Y5023/RT455	*MAT*a/*MAT*α *ade2-1/ade2-1::OsTIR1-9myc:ADE2 BRN1-3mNeonGreen::HIS3/BRN1-3mNeonGreen::NAT SMC4*^{7A}*-CLB2*$^{\Delta CDK}$*-3HA:kanR:URA3/SMC4*^{7A}*-CLB2*$^{\Delta CDK}$*-3HA:TRP1:URA3 leu2-2,112/leu2-2,112::P*$_{GAL1}$*–MAD2-MAD3–Cre-EBD78*	W303	This study	S6A
Y5027/RT463	*MAT*a *ade2-1::OsTIR1-9myc:ADE2 NET1-yEmCitrine::HIS3 SMC4-CLB2:TRP1 leu2-3,112::P*$_{GAL1}$*–MAD2-MAD3–Cre-EBD78*	W303	This study	S6C-D
Y5029/RT467	*MAT*a *ade2-1::OsTIR1-9myc:ADE2 NET1-yEmCitrine::HIS3 SMC4-CLB2*$^{\Delta CDK}$*:TRP1 leu2-3,112::P*$_{GAL1}$*–MAD2-MAD3–Cre-EBD78*	W303	This study	S6C-D

SUPPLEMENTAL REFERENCES

Foiani, M., Marini, F., Gamba, D., Lucchini, G., and Plevani, P. (1994). The B subunit of the DNA polymerase a-primase complex in *Saccharomyces cerevisiae* executes an essential function at the initial stage of DNA replication. Mol Cell Biol *14*, 923-933.

Gietz, R.D., and Sugino, A. (1988). New yeast-*Escherichia coli* shuttle vectors constructed with in vitro mutagenized yeast genes lacking six-base restriction sites. Gene *74*, 527-534.

Kapur, J.N., Sahoo, P.K., and Wong, A.C.K. (1985). A new method for gray-level picture thresholding using the entropy of the histogram. Graph Models Image Process *29*, 273-285.

Knop, M., Siegers, K., Pereira, G., Zachariae, W., Winsor, B., Nasmyth, K., and Schiebel, E. (1999). Epitope tagging of yeast genes using a PCR-based strategy: more tags and improved practical routines. Yeast *15*, 963-972.

Kubota, T., Nishimura, K., Kanemaki, M.T., and Donaldson, A.D. (2013). The Elg1 replication factor C-like complex functions in PCNA unloading during DNA replication. Mol Cell *50*, 273-280.

Kuilman, T., Maiolica, A., Godfrey, M., Scheidel, N., Aebersold, R., and Uhlmann, F. (2014). Identification of Cdk targets that control cytokinesis. EMBO J *34*, 81-96.

Marbouty, M., Le Gall, A., Cattoni, D.I., Cournac, A., Koh, A., Fiche, J.B., Mozziconacci, J., Murray, H., Koszul, R., and Nollmann, M. (2015). Condensin- and replication-mediated bacterial chromosome folding and origin condensation revealed by Hi-C and super-resolution imaging. Mol Cell *59*, 588-602.

O'Reilly, N., Charbin, A., Lopez-Serra, L., and Uhlmann, F. (2012). Facile synthesis of budding yeast **a**-factor and its use to synchronize cells of a mating type. Yeast *29*, 233-240.

Ries, J., Kaplan, C., Platonova, E., Eghlidi, H., and Ewers, H. (2012). A simple, versatile method for GFP-based super-resolution microscopy via nanobodies. Nat Methods *9*, 582-584.

Rose, M.D., Winston, F., and Hieter, P. (1990). Laboratory course manual for methods in yeast genetics (Cold Spring Harbor, NY: Cold Spring Harbor Laboratory Press).

Sheff, M.A., and Thorn, K.S. (2004). Optimized cassettes for fluorescent protein tagging in *Saccharomyces cerevisiae*. Yeast *21*, 661-670.
